# Bidirectional Mendelian Randomization and Multi-Omics Uncover Causal Serum Metabolites and Neuro-Related Mechanistic Pathways in Acute Myeloid Leukemia

**DOI:** 10.3390/ijms262311307

**Published:** 2025-11-22

**Authors:** Haohan Ye, Yuanheng Liu, Jun Tang, Xiaoli Li

**Affiliations:** 1 School of Basic Medical Sciences, Chongqing Medical University, Chongqing 400016, China; 2 Center for Experimental Teaching Management, Chongqing Medical University, Chongqing 400016, China; 3 Laboratory of Developmental Biology, Department of Cell Biology and Genetics, School of Basic Medical Sciences, Chongqing Medical University, Chongqing 400016, China

**Keywords:** serum metabolites, acute myeloid leukemia, mendelian randomization, transcriptome, metabolic pathways

## Abstract

Acute myeloid leukemia (AML) is a lethal clonal hematopoietic malignancy. Several reports have shown that serum metabolite alterations have been implicated in AML, but the causal relationship and underlying biological mechanisms remain unclear. We performed bidirectional Mendelian randomization (MR) to evaluate the association between 486 serum metabolites and AML. The analytical approaches used to minimize research bias included the inverse variance weighting (IVW), MR-Egger and weighted median (WM) methods. Sensitivity analyses were performed using Cochran’s Q Test, MR-Egger, MR pleiotropy residual sum and outlier (MR-PRESSO), and Leave-one-out (LOO) analysis. Metabolic pathway analysis was conducted using the MetaboAnalyst 6.0 platform. We utilized RNA-seq data to explore the potential genes and mechanisms underlying the regulation of AML occurrence by serum metabolites. We identified 23 serum metabolites (13 known and 10 unknown) significantly associated with AML. Sensitivity analyses further validated the robustness of these associations. No evidence of reverse causality was detected by reverse MR analysis. The core pathways were histidine metabolism and fructose/mannose metabolism. Transcriptomic integration revealed 39 overlapping genes (differentially expressed genes vs. metabolite-associated genes) as key mediators, enriched in neuroactive ligand signaling, synaptic vesicle cycle, and GABAergic synapse (KEGG), plus synapse assembly and calmodulin binding and neuron-to-neuron synapse (GO). This study establishes causal links between specific serum metabolites and AML, revealing neuro-related mechanistic pathways. These findings provide novel biomarkers and therapeutic targets for AML precision medicine.

## 1. Introduction

Acute myeloid leukemia (AML) is a biologically heterogeneous malignancy originating from clonally expanding hematopoietic stem cells. Sequential accumulation of genetic mutations drives the expansion of clonal myeloid progenitor cells while impairing their differentiation capacity, ultimately leading to fulminant AML [1]. It is the most prevalent subtype of acute leukemia in adults, with a median age of diagnosis of 68 years [2]. Its defining feature is excessive proliferation of abnormal primitive or immature cells in the bone marrow, which suppresses normal hematopoiesis and leads to clinical manifestations such as anemia, infections, bleeding, and organ infiltration [3,4]. According to global cancer statistics, the incidence rate of AML is approximately 1.2 to 4.2 per 100,000 individuals [5]. AML has demonstrated a sustained increase in incidence over recent years. Current therapeutic approaches for AML include chemotherapy, hematopoietic stem cell transplantation and targeted therapy; however, treatment outcomes remain suboptimal due to the inherent complexity and heterogeneity of the disease [6,7]. Therefore, elucidating the molecular mechanisms underlying the pathogenesis of AML and developing novel therapeutic strategies are crucial for improving clinical outcomes.

While the exact molecular underpinnings of AML pathogenesis remain poorly defined, mounting evidence points to a multifactorial interplay of inherited susceptibility, epigenetic dysregulation, and exogenous exposures. Notably, recent advancements in metabolomics have unveiled intricate perturbations in serum metabolite profiles, particularly those linked to oncogenic signaling pathways, indicating a critical role of metabolic dysregulation in the development of leukemia [7]. Metabolomic profiling of serum samples from patients with AML revealed significant dysregulation in glycogenic amino acid levels associated with the glycolytic pathway, along with elevated levels of products derived from protein degradation pathways. Notably, distinct biomarker panels were observed in each malignant tumor cohort compared to the control group. These findings suggest the presence of common metabolic adaptation patterns prevalent in AML [8]. Comprehensive metabolic profiling via untargeted metabolomics has systematically identified dysregulated pathways and candidate biomarkers that differentiate patient and control cohorts [9]. These molecular signatures not only enhance diagnostic accuracy but also demonstrate prognostic utility in predicting treatment responses and survival outcomes [10]. Fms-like tyrosine kinase 3 inhibitors targeting the most prevalent mutation in AML and Bcl-2 inhibitors directed against conserved oncogenic signaling pathways have emerged as the first-generation molecularly targeted agents in precision medicine for AML therapy [11,12]. Other oncogenic drivers, such as MYC and RAS mutations, have been demonstrated to orchestrate metabolic reprogramming characterized by enhanced glycolysis, glutaminolysis, lipogenesis, and mitochondrial biogenesis, which are critical for AML cell proliferation and survival [13]. Furthermore, AML cells induce insulin desensitization through elevated serum insulin-like growth factor binding protein 1 levels, thereby impairing glucose uptake in normal tissues [14]. The aforementioned evidence demonstrates a robust association between serum metabolites and AML [15]. However, the preponderance of extant evidence stems from basic research, which are inherently susceptible to confounding biases and reverse causation. Hence, a systematic interrogation of the causal relationship between serum metabolites and AML is imperative.

Mendelian Randomization (MR) harnesses genome-wide association study (GWAS)-derived single-nucleotide polymorphisms (SNPs) as instrumental variables to dissect the directional causality between serum metabolites (exposures) and AML (outcomes), overcoming confounding biases inherent in traditional observational analyses [16]. By leveraging Mendelian principles of random assortment during gamete formation, this analytical strategy diminishes the influence of unmeasured errors and confounding factors through adhering to genetic principles, while mitigating the bias caused by reverse causality [17]. MR has emerged as a pivotal tool in cancer research, enabling systematic dissection of causal pathways in malignancies. Here, we integrate bidirectional MR with multi-omics (metabolomics + transcriptomics) to identify causal serum metabolites for AML, map their associated metabolic pathways, and uncover downstream transcriptional mechanisms linking metabolites to AML. Our findings bridge the gap between causal inference and functional mechanism, providing actionable insights for AML precision oncology.

## 2. Results

### 2.1. Strength of the Instrumental Variables

To assess the causal effects of serum metabolites on AML using GWAS summary datasets, We conducted a two-sample MR analysis. For the 486 metabolites examined, each was associated with 3 to 307 SNPs serving as IVs; fructose and ergothioneine had the fewest IVs (3 SNPs each), while 2-methoxyacetaminophen sulfate* exhibited the most (307 SNPs). Critically, the minimum F-statistic across all IVs was 17.45, which exceeds the conventional threshold of 10 for ensuring the robustness of IVs in MR analyses.

### 2.2. Mendelian Randomization Analysis Results

**Figure 1:**
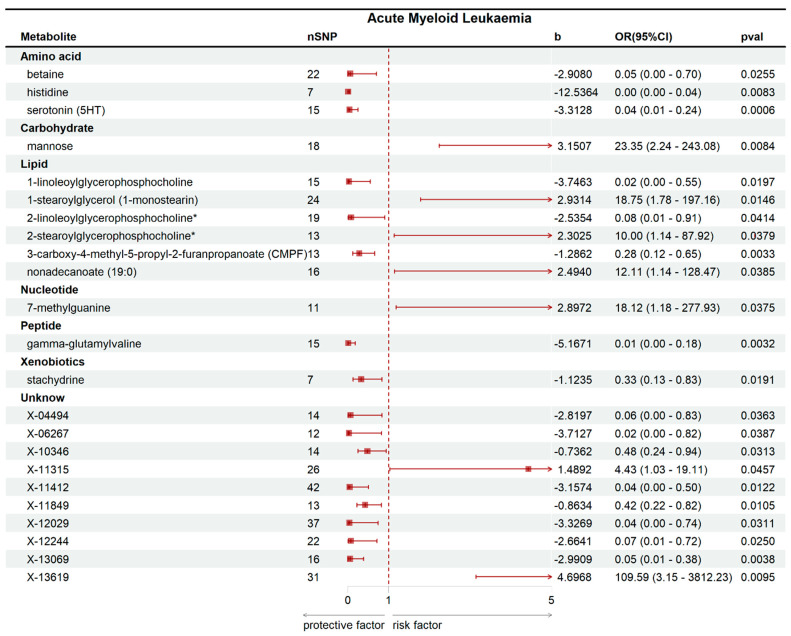
Forest plots showing causal estimates between serum metabolites and AML in forward MR. * Indicates metabolites for which reference spectra of the pure substances were not directly measured on the Metabolon platform.

**Figure 2:**
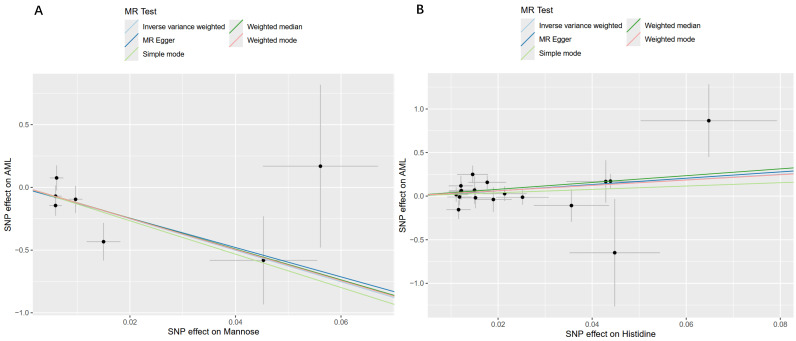
Scatter plot of the genetic association between top-risk and top-protective metabolites and AML risk. (**A**) Genetic association between top-risk metabolites and AML risk. (**B**) Genetic association between top-protective metabolites and AML risk.

Using the IVW method within a MR framework, we analyzed associations between 486 serum metabolites and AML. The analysis identified 23 metabolites with significant associations (13 identified metabolites and 10 unidentified metabolites), as visualized in Figure 1 and Table 1. Five identified metabolites showed positive associations with AML onset: 2-stearoylglycerophosphocholine* (OR: 9.9996, 95%CI: 1.1373–87.9194, *p* = 0.0379), nonadecanoate (19:0) (OR: 12.1093, 95%CI: 1.1414–128.4657, *p* = 0.0385), 7-methylguanine (OR: 18.1241, 95%CI: 1.1819–277.9256, *p* = 0.0375), 1-stearoylglycerol (1-monostearin) (OR: 18.7536, 95%CI: 1.7838–197.1577, *p* = 0.0146), and mannose (OR: 23.3522, 95%CI: 2.2434–243.0828, *p* = 0.0084). The unidentified metabolites also exhibited positive associations with AML, including X-11315 and X-13619. Eight identified metabolites were inversely associated with AML risk, indicating potential protective effects: histidine (OR: 0.0000, 95%CI: 0.0000–0.0394, *p* = 0.0083), gamma-glutamylvaline (OR: 0.0057, 95%CI: 0.0002–0.1778, *p* = 0.0032), 1-linoleoylglycerophosphocholine (OR: 0.0236, 95%CI: 0.0010–0.5498, *p* = 0.0197), serotonin (5HT) (OR: 0.0364, 95%CI: 0.0054–0.2443, *p* = 0.0007), betaine(OR: 0.0546, 95%CI: 0.0043–0.6999, *p* = 0.0255), 2-linoleoylglycerophosphocholine* (OR: 0.0792, 95%CI: 0.0069–0.9060, *p* = 0.0414), 3-carboxy-4-methyl-5-propyl-2-furanpropanoate (CMPF) (OR: 0.2763, 95%CI: 0.1171–0.6519, *p* = 0.0033), and stachydrine (OR: 0.3251, 95%CI: 0.1271–0.8319, *p* = 0.0191). Additionally, 8 unidentified metabolites showed negative associations with AML, including X-06267, X-12029, X-11412, X-13069, X-04494, X-12244, X-11849 andX-10346. Notably, mannose—a carbohydrate metabolite—demonstrated the strongest positive causal effect on AML development, while histidine—an amino acid metabolite—exhibited the most pronounced protective effect against AML (Figure 2 and Figure S1).

### 2.3. Sensitivity and Reverse Causality Analysis Results

To assess the consistency of genetic effects across the 23 metabolites significantly associated with AML, we employed Cochran’s Q test within both IVW and MR-Egger models. No significant heterogeneity was detected in either analysis (all *p* > 0.05), indicating consistent causal estimates across the selected SNPs. Comprehensive results of these tests are summarized in Table 1. We further evaluated horizontal pleiotropy using three complementary approaches. First, MR-Egger regression revealed no statistically significant intercept (*p* > 0.05), suggesting no systematic directional pleiotropy between the genetic instruments and AML risk. Second, MR-PRESSO analysis, which identifies and adjusts for pleiotropic outliers, yielded a non-significant global test (*p* > 0.05) and no individual SNPs were flagged as influential outliers (Figure S2). These findings collectively indicate that pleiotropy did not confound the causal relationship between the metabolites and AML. Third, the LOO sensitivity analysis confirmed the robustness of our results: removing any single SNP did not substantially alter the causal estimates for any metabolite, ruling out dominance by a single genetic variant (Figure S3). To address potential reverse causality, we conducted a reverse MR analysis with AML as the exposure and the 23 metabolites as outcomes. No significant causal associations were observed in this analysis (Table S1), further strengthening the validity of our forward MR findings.

### 2.4. Results of Metabolic Pathway Enrichment Analysis

**Figure 3:**
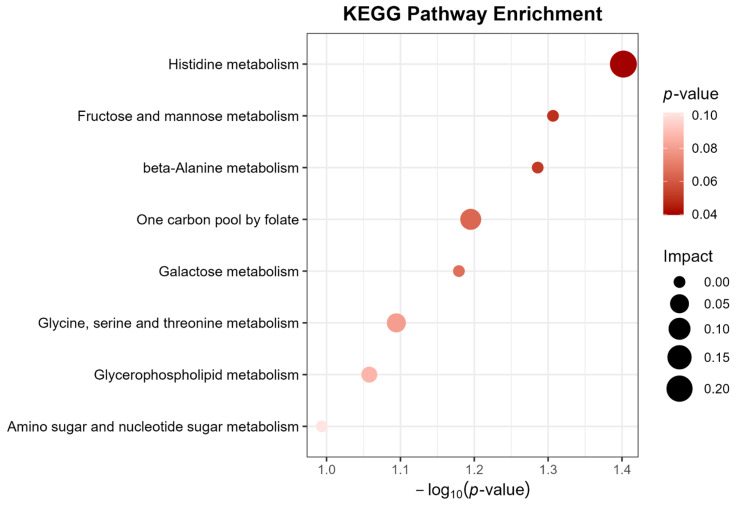
Displays the enrichment pathways of metabolites in KEGG.

Through metabolic pathway analysis, two key pathways predominantly implicated in AML pathogenesis were elucidated, as visualized in Figure 3 and Table S2. Enrichment analysis further corroborated the biological relevance of these pathways, highlighting the histidine metabolism pathway and the fructose/mannose metabolism pathways as central to AML-associated metabolic dysregulation.

### 2.5. Mapping SNPs to Genes and DEGs Identification of AML

**Figure 4:**
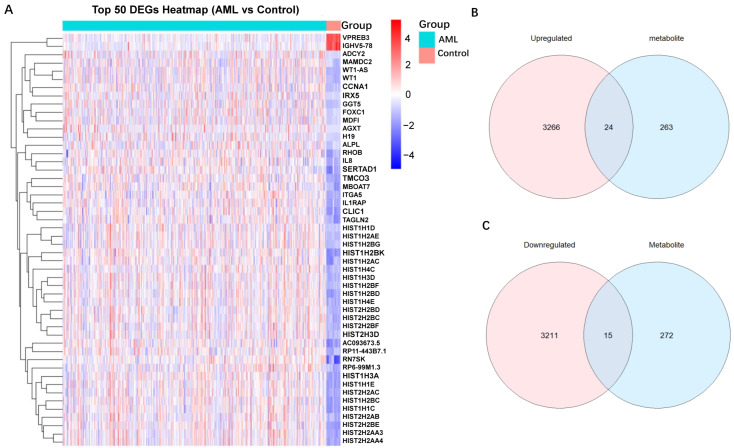
Identification of overlapping genes between serum metabolite-associated genes and DEGs in AML. (**A**) A heatmap of the top 50 DEGs in AML. (**B**) Venn diagram showing 24 overlapping genes between upregulated DEGs and metabolite-associated genes. (**C**) Venn diagram showing 15 overlapping genes between downregulated DEGs and metabolite-associated genes. *AML* acute myeloid leukemia, *DEGs* Differentially Expressed Genes.

By querying the online database SNPnexus, we obtained 287 metabolite-associated genes. By comparing gene expression profiles between the normal group and the AML group, we identified 6516 DEGs, comprising 3290 upregulated and 3226 downregulated genes. A heatmap was generated to visualize the differential expression patterns of these DEGs across samples (Figure 4A).

### 2.6. Identification and Functional Enrichment Analysis of Overlapping Genes

**Figure 5:**
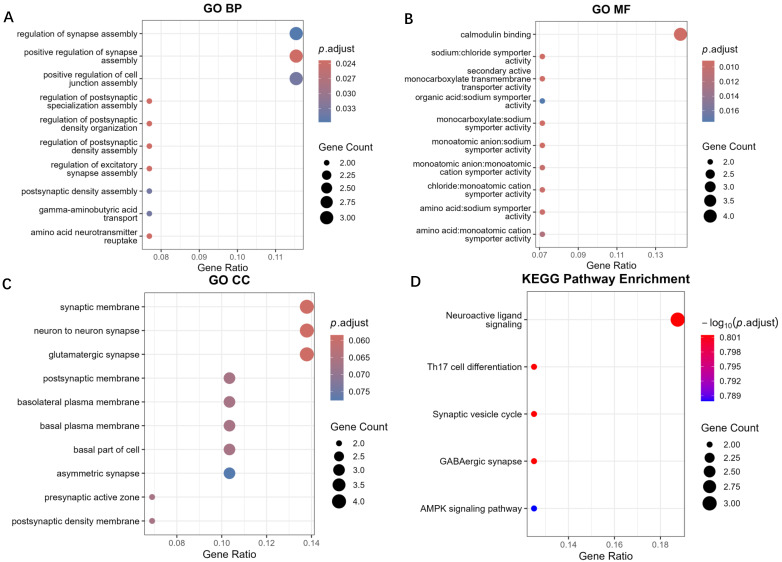
Functional analysis of overlapping genes between serum metabolite-associated genes and DEGs in AML. (**A**) GO enrichment analysis for overlapping genes in the category of Biological Processes. (**B**) GO enrichment analysis for overlapping genes in the category of Molecular Functions. (**C**) GO enrichment analysis for overlapping genes in the category of Cellular Components. (**D**) KEGG pathway enrichment analysis for overlapping genes. *AML* acute myeloid leukemia, *GO* Gene Ontology, *KEGG* Kyoto Encyclopedia of Genes and Genomes.

To identify overlapping genes between metabolite-associated genes and DEGs, we intersected the two gene sets and generated two Venn diagrams, which showed 24 overlapping genes between upregulated DEGs and metabolite-associated genes, as well as 15 overlapping genes between downregulated DEGs and metabolite-associated genes (Figure 4B,C). To deeply explore the biological processes associated with overlapping genes between serum metabolites and AML, we performed GO and KEGG pathway analysis. GO analysis results revealed that these genes were predominantly enriched in positive regulation of synapse assembly, calmodulin binding, and neuron to neuron synapse (Figure 5A–C and Table S3). Furthermore, KEGG pathway enrichment analysis highlighted their significant involvement in key cellular functions, including neuroactive ligand signaling, synaptic vesicle cycle, and GABAergic synapse (Figure 5D).

## 3. Discussion

To the best of our knowledge, no prior studies have investigated the causal relationships between serum metabolites and AML using MR analysis. Our pioneering systematic study employed MR analysis with pathway enrichment and transcriptomic data to dissect the role of serum metabolites in AML pathogenesis. Using GWAS data, we identified 23 serum metabolites (13 with known and 10 uncharacterized) significantly associated with AML risk among 486 candidate metabolites. Of these, 5 metabolites exhibited a positive causal relationship with increased AML susceptibility, while 8 metabolites showed a negative association—suggesting potential protective effects against AML development. Pathway enrichment analysis of the identified metabolites revealed two canonical metabolic pathways as key drivers: histidine metabolism and the fructose and mannose metabolism. Further integration of transcriptomic data revealed that 39 overlapping genes influenced by these metabolites were significantly enriched in pathways such as neuroactive ligand signaling and synaptic vesicle cycle. These findings suggest that metabolites may participate in the development and progression of AML by regulating neuro-related genes, providing novel biomarkers and therapeutic targets for the precise diagnosis and treatment of AML.

Our study identified the carbohydrate metabolite mannose levels as risk factors for the onset of AML. Mannose is a bioactive monosaccharide that plays a critical regulatory role in glycosylation processes, energy metabolism pathways, and cancer immunotherapy. A study had revealed that circulating mannose levels exhibit a positive correlation with obesity-independent insulin resistance, a relationship mediated by mannose-induced impairment of insulin receptor function or its participation in advanced glycation end product formation [18]. A study further demonstrated that cell-intrinsic mannose metabolism acts as a critical physiological regulator of CD8+ T cell fate, thereby decoupling proliferation/expansion programs from differentiation, and further highlights the therapeutic potential of mannose modulation metabolic in cancer immunotherapy [19]. Our findings further reveal that mannose emerges as the most potent risk factor for AML, likely mediating its effects through pro-inflammatory mechanisms and glycosylation processes, thereby underscoring its promise as a biomarker and therapeutic target for AML.

Our study also identified that elevated levels of amino acid metabolite histidine exert the strongest protective effect for AML. Histidine can generate histamine via an enzymatic decarboxylation catalyzed by histidine decarboxylase (HDC), which is subsequently oxidized to form imidazoleacetic acid and imidazoleacetaldehyde. Histamine is recognized as the quintessential inflammatory mediator. The release of histamine from mast cells represents a response triggered by the binding of IgE to mast cell membrane receptors, serves as part of the allergic response. As a key mediator in allergic pathologies, histidine exerts complex effects on inflammatory cascades. Its association with allergic disorders such as atopic dermatitis is characterized by modulation of the Th1/Th2 immune balance [20]. Emerging evidence from recent studies suggests that histidine could represent a key therapeutic target for allergic diseases, particularly allergic rhinitis and asthma [21]. The investigations in breast carcinoma and prostate cancer have implicated the His variant of CASP8, D302H (rs1045485) as a protective risk allele [22]. In our study, we identified histidine as the most potent protective factor against AML, suggesting that histidine may exert an anti-inflammatory role during AML pathogenesis and could serve as a key biomarker for AML. Nonetheless, to gain a deeper understanding of the role of metabolites in AML, additional clinical investigations and experimental validation are warranted.

Notably, the metabolites identified in our study are predominantly enriched in the histidine metabolism pathway, as well as the fructose and mannose metabolism pathways. The amino acid metabolite histidine has been discussed above. Multiple studies have shown that fructose and mannose metabolism is closely linked to glycolysis and may serve as a critical source of substrates for sugar nucleotide biosynthesis [23]. Fructose and mannose metabolism has also been implicated in cancer progression. For instance, one study revealed that fructose and mannose metabolism represent a key metabolic hallmark of protumor and prometastasis macrophage subsets, potentially serving as an actionable target for reprogramming macrophage phenotypes and the tumor microenvironment in head and neck squamous cell carcinoma [24]. The fructose and mannose metabolism signaling pathways are also implicated in the pathogenesis of septic shock among pediatric patients [25]. Altogether, these findings indicate that fructose and mannose biosynthesis may play a critical role in the pathobiological mechanisms of AML, potentially through regulating energy metabolism and immune response of AML cells.

To elucidate the association between metabolites and DEGs, we identified 39 overlapping genes between metabolite-associated genes and DEGs. These genes were key mediators in the link between metabolites and AML risk, potentially playing a critical intermediary role in connecting the two. Among those genes, *TTN* has been identified as being associated with AML [26]. Therefore, *TTN* may serve as a key gene in the pathogenesis of AML, and its expression could be regulated by metabolites to promote AML progression. Monitoring its expression levels in AML patients might facilitate the prediction of secondary AML risk. The enrichment of neuroactive pathways (e.g., synaptic vesicle cycle, GABAergic synapse) in our analysis aligns with emerging evidence linking metabolic reprogramming to neuronal signaling in cancer. AML cells are known to exhibit metabolic plasticity, and recent studies highlight crosstalk between altered metabolism and neuro-related signaling [27]. For instance, GABA signaling—typically associated with neuronal function—has been shown to inhibit GSK-3β activity via GABAB receptor activation, thereby enhancing β-catenin signaling-mediated tumor growth and immune suppression [28]. These findings suggest that metabolites may engage neuro-related genes to facilitate AML progression, highlighting a potential therapeutic axis for targeting this interplay.

This study had several limitations. First, the validity of MR analysis is primarily determined by the explanatory power of IVs regarding exposure. Thus, expanding the sample size is essential to enable a more precise evaluation of the genetic influence on metabolites. Second, although MR has been demonstrated to be a robust approach for inferring causal relationships between serum metabolites and AML, its findings necessitate validation through additional experimental validation. Future studies will validate these associations using PCR for key metabolite-associated genes in primary AML samples to directly confirm the mechanistic links identified by MR. Third, functional validation is lacking for both unknown metabolites and the identified neuro-related pathways; in vitro experiments (e.g., CRISPR knockout of GABAB receptors) could be performed to confirm the involvement of these neuro pathways. Fourth, our focus on cis-regulatory mechanisms using SNPnexus limited the exploration of trans-regulatory effects, which may play critical roles in metabolite-associated gene expression. Future work integrating TWAS with eQTL data could refine the functional interpretation of metabolite-associated loci [29].

## 4. Materials and Methods

### 4.1. Study Design

**Figure 6:**
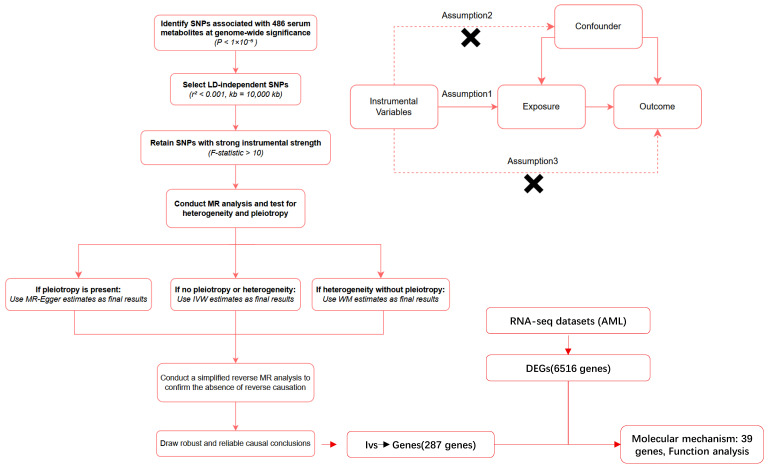
The overall designation of this study.

To investigate the causal relationship between serum metabolites and AML susceptibility, we conducted this MR study. Initially, we systematically screened 486 serum metabolites for causal associations with AML using a two-sample MR framework (Figure 6). MR utilizes genetic variants as instrumental variables (IVs) to infer causal relationships between risk factors and outcomes, thereby reducing confounding bias while maintaining methodological robustness. Valid IVs in MR must satisfy three core assumptions: (1) the genetic variants must be robustly correlated with the exposure of interest; (2) the instruments must not share any common causes with the outcome that could bias the association; (3) the only pathway through which the genetic instruments affect the outcome is via their influence on the exposure. Causal inference was performed using IVW, MR-Egger, and weighted median methods, with sensitivity analyses (Cochran’s Q test, MR-PRESSO, leave-one-out validation) to assess robustness. Considering the secondary use of publicly available data and previously published aggregated statistical data, ethical approval was granted. Evaluate metabolic pathway enrichment using the MetaboAnalyst 6.0 platform. To explore potential mechanisms, we integrated RNA seq data from AML patients, identified overlapping genes between differentially expressed genes and metabolite related genes, and identified the functions of these key genes through gene set enrichment analysis. This multi omics approach ensures comprehensive validation of the causal relationships and functional explanations of metabolites in AML. The study employed R version 4.3.3, supplemented with the TwoSampleMR package (version 0.5.11).

### 4.2. Data Source

The GWAS dataset for serum metabolites was obtained from the large-scale study by Shin et al. [30], which stands as the most extensive characterization of serum metabolite genetic associations to date. Utilizing high-throughput metabolic profiling, this research identified 2.1 million SNPs linked to 486 distinct metabolites. The study included 7824 subjects, comprising 1768 participants from Germany and 6056 from the United Kingdom. All participants voluntarily provided signed consent documents, and the research was ethically sanctioned by independent ethics committees affiliated with the participating institutions. Among the 486 metabolites, 177 were classified as unknown, while 309 were classified into known categories. The AML GWAS data were accessed via the FinnGen R12 release “https://www.finngen.fi/en (accessed on 5 June 2025)” [31]. Specifically, we utilized the curated AML subset (data identifier: C3_AML_EXALLC; “https://storage.googleapis.com/finngen-public-data-r12/summary_stats/release/finngen_R12_C3_AML_EXALLC.gz (accessed on 5 June 2025)”), encompassing 322 adult AML cases and 378,747 controls.

### 4.3. Selection of Instrumental Variables (IVs)

To identify IVs, we adopted a systematic three-step approach: (1) initial variant selection involved mining GWAS data for genetic variants robustly associated with the 486 serum metabolites (*p* < 1 × 10^−5^), a threshold validated across major consortia to ensure minimal false-positive signals [32]. (2) Aggregated SNPs were evaluated for linkage disequilibrium (LD) independence, with IVs selected from non-linked metabolic loci (parameters: 10,00kb distance, r^2^ < 0.001) [33]. (3) finally, variants underwent F-statistic filtering (F > 10) in two-stage regression models to confirm sufficient strength as instruments, aligning with MR methodological standards to avoid weak-instrument bias [34]. The F-statistic was derived through the formula F = R^2^ × (n – k − 1)/[k × (1 − R^2^)]. Here, R^2^ denotes the proportion of variance in the exposure variable (e.g., serum metabolites) explained by the IVs, calculated as R^2^ = 2 × (1 − MAF) × MAF × β^2^. In this formula, n represents the exposure-specific sample size, k denotes the instrumental variable count, MAF corresponds to the minor allele frequency, and β indicates the genetic effect size [34].

### 4.4. Mendelian Randomization Analyses

This study primarily employed the IVW method to assess causal relationships between serum metabolites and AML, assuming valid and independent genetic instruments. IVW constrains the intercept to zero and weights variants by the inverse of outcome variance, yielding precise effect estimates when assumptions hold [35,36]. In this study, the IVW-derived *p*-value was employed as the primary indicator to assess the causal relationship between exposure and outcomes. To enhance robustness, we integrated MR-Egger regression (testing for horizontal pleiotropy via an unconstrained intercept) and WM analysis (estimating effects via robust medians, tolerant to up to 50% invalid instruments) [37,38]. Consistency checks across methods (IVW/MR-Egger/WM) and reverse MR (testing metabolite-to-AML effects) validated causal directionality. This pipeline ensured rigorous, bias-minimized causal inference while balancing statistical power and methodological transparency.

### 4.5. Sensitivity Analysis

To assess the consistency of genetic variant effects on the exposure, we employed Cochran’s Q test, where a *p*-value < 0.05 indicates substantial heterogeneity among the selected SNPs [39]. For evaluating horizontal pleiotropy, we integrated two complementary approaches: MR-Egger regression, which uses the intercept term to detect systematic pleiotropy (a *p*-value < 0.05 for the intercept suggests its presence), and MR-PRESSO test, which identifies and adjusts for outlying SNPs that may bias causal estimates (a global *p*-value < 0.05 indicates significant pleiotropy) [40,41,42]. To further ensure the robustness of our causal inferences, we performed a LOO sensitivity analysis, where each SNP was sequentially excluded to examine whether the overall effect estimates remained stable (i.e., no single SNP drove the results) [41]. Together, these methods provided a comprehensive framework for validating the reliability of our MR findings.

### 4.6. Metabolic Pathway Analysis

To explore the metabolic pathways associated with AML pathogenesis, we performed metabolic pathway analysis using the MetaboAnalyst 6.0 platform “https://www.metaboanalyst.ca/ (accessed on 17 June 2025)” [43]. The analysis focused on identifying pathways linked to the biological processes driving AML, leveraging KEGG as the primary pathway library. For the enrichment analysis, we employed the hypergeometric test, a widely used statistical method for evaluating the overrepresentation of specific metabolites or genes in predefined pathways. A significance threshold of *p* < 0.05 was set to filter for biologically relevant pathways, ensuring that the identified associations were statistically robust and not due to random chance. This approach allowed us to prioritize pathways with strong evidence of involvement in AML, providing a foundation for further investigation into the metabolic mechanisms underlying the disease.

### 4.7. Mapping SNPs to Genes and Identification of Differentially Expressed Genes

Our study prioritized cis-regulatory mechanisms linking serum metabolites to AML risk. Using the web-based variant annotation tool SNPnexus “https://www.snp-nexus.org/v4/ (accessed on 17 July 2025)”, we annotated each queried variant to its nearest gene, encompassing overlapping, downstream, or upstream genes [44]. We downloaded the Beat 2.0 AML dataset “http://www.vizome.org (accessed on 17 July 2025)”, which includes 671 AML samples and 36 non-AML samples. This dataset includes 671 AML samples and 36 control samples. The data was generated using a high-throughput sequencing platform. Using the limma package (version 3.58.1), we identified DEGs between the AML and healthy control groups. The criteria for identifying DEGs were set as a stringent threshold of Log_2_ Fold Change (FC) > 1 and a significance level of adjusted *p*-value < 0.05. 

### 4.8. Identification and Functional Enrichment Analysis of Overlapping Genes

To investigate the correlation between metabolites and DEGs, we identified overlapping genes between metabolite-associated genes and DEGs. These overlapping genes were key mediators in the association between metabolites and AML risk. Subsequently, we performed GO enrichment analysis and KEGG pathway enrichment analysis using the ‘ClusterProfiler’ R package (version 4.10.1) to elucidate the biological mechanisms underlying these overlapping genes [45].

## 5. Conclusions

In conclusion, this study establishes causal links between 23 serum metabolites (13 known, 10 unknown) and AML via bidirectional MR, with histidine and fructose/mannose metabolism as core drivers. Transcriptomic integration further reveals that these metabolites may act through neuroactive pathways (e.g., synaptic signaling) to promote AML. These findings provide novel biomarkers (known/unknown metabolites) and therapeutic targets (neuro-related pathways) for AML precision medicine, bridging causal inference with mechanistic insight.

## Figures and Tables

**Table 1:** MR model estimated causal relationship between 23 metabolites and the risk of AML and tested for heterogeneity and horizontal pleiotropy.

Metabolite	nSNP	Cochran’s Q Test		MR-Egger Intercept		MR-Presso	
		IVW	MR Egger	Egger Intercept	*p*	Global Test RSSobs	*p*
1-linoleoylglycerophosphocholine	15	0.8486	0.8081	−0.0343	0.6631	9.6823	0.8850
1-stearoylglycerol (1-monostearin)	24	0.4880	0.4975	0.0561	0.2931	25.0529	0.4780
2-linoleoylglycerophosphocholine*	19	0.4243	0.4240	0.0812	0.3312	20.5221	0.4520
2-stearoylglycerophosphocholine*	13	0.7440	0.7082	0.0852	0.5142	9.7489	0.7800
3-carboxy-4-methyl-5-propyl-2-furanpropanoate (CMPF)	13	0.5016	0.4208	−0.0152	0.8307	13.2670	0.5190
7-methylguanine	11	0.1743	0.1569	−0.0680	0.4662	17.6698	0.2050
betaine	22	0.8676	0.8863	−0.0540	0.2752	15.0725	0.8750
gamma-glutamylvaline	15	0.6318	0.5545	0.0087	0.9317	12.9875	0.6810
histidine	7	0.2744	0.1854	−0.0108	0.9069	9.9380	0.3410
mannose	18	0.4115	0.3479	−0.0116	0.8398	19.3056	0.4870
nonadecanoate (19:0)	16	0.6602	0.6815	−0.0546	0.2937	13.9014	0.6900
serotonin (5HT)	15	0.3151	0.2509	−0.0027	0.9552	19.0807	0.3560
stachydrine	7	0.8902	0.8169	0.0421	0.7988	2.9697	0.9170
X-04494	14	0.6025	0.6016	−0.0435	0.3526	12.3367	0.6670
X-06267	12	0.3642	0.2871	0.0100	0.8938	14.0074	0.4160
X-10346	14	0.4221	0.3450	0.0012	0.9842	14.4879	0.5270
X-11315	26	0.6334	0.5944	−0.0169	0.5934	2.9697	0.9170
X-11412	42	0.3686	0.4152	0.0494	0.1560	45.1492	0.3860
X-11849	13	0.6487	0.5765	−0.0223	0.7216	11.6005	0.6570
X-12029	37	0.4456	0.4488	0.0466	0.3075	38.5584	0.5010
X-12244	22	0.3725	0.3544	−0.0410	0.4223	24.2208	0.4590
X-13069	16	0.8026	0.7424	0.0021	0.9835	11.5354	0.8040
X-13619	31	0.5595	0.5816	−0.0624	0.2479	30.2791	0.5500

nSNP: The number of single-nucleotide polymorphisms (SNPs). Cochran’s Q test: Tests if the variation in results across studies is greater than expected by chance. A *p*-value > 0.05 indicates no significant heterogeneity between studies. IVW: Giving more weight to studies with more precise estimates. An IVW estimate of 1.5 suggests a positive effect with a 1.5 times increase in the outcome for each unit increase in the exposure. MR-Egger Intercept: Measures horizontal pleiotropy (bias from confounding). An intercept close to 0 indicates no significant bias. Egger intercept: Similar to the MR-Egger intercept. A non-zero intercept suggests possible pleiotropic effects. MR-PRESSO: Detects and corrects for outliers in Mendelian randomization. A corrected *p*-value < 0.05 suggests the results are robust after outlier correction. Global Test RSSobs: Measures how well the model fits the data. A lower RSSobs indicates a better model fit. *p*: Indicates statistical significance. A *p*-value < 0.05 suggests significant result. * Indicates metabolites for which reference spectra of the pure substances were not directly measured on the Metabolon platform.

## Data Availability

The original contributions presented in this study are included in the article/Supplementary Materials. Further inquiries can be directed to the corresponding author(s). The data supporting the findings of this study are publicly available from several sources. Publicly accessible datasets used in this study include those from the R12 version of the FinnGen database “https://www.finngen.fi/en (accessed on 5 June 2025)”, the AML dataset IDs were C3_AML_EXALLC “https://storage.googleapis.com/finngen-public-data-r12/summary_stats/release/finngen_R12_C3_AML_EXALLC.gz (accessed on 5 June 2025)”, the Beat 2.0 AML dataset “http://www.vizome.org (accessed on 17 July 2025)”.

## Supplementary Materials

The supporting information can be downloaded at: https://www.mdpi.com/article/10.3390/ijms262311307/s1.

## Abbreviations

AML: Acute myeloid leukemia; MR: Mendelian randomization; IVW: inverse variance weighted; WM: weighted median; MR-PRESSO: MR pleiotropy residual sum and outlier; GWAS: Genome wide association study; SNPs: Single-nucleotide polymorphisms; IVs: Instrumental variables; LD: linkage disequilibrium; DEGs: differentially expressed genes; KEGG: Kyoto encyclopedia of genes and genomes; GO: Gene Ontology; LOO: leave-one-out; FC: Fold Change; CMPF: 3-carboxy-4-methyl-5-propyl-2-furanpropanoate.
