# Supplementary material for: Bidirectional Mendelian Randomization and Multi-Omics Uncover Causal Serum Metabolites and Neuro-Related Mechanistic Pathways in Acute Myeloid Leukemia
Source: Int J Mol Sci. 2025 Nov 22;26(23):11307. doi: 10.3390/ijms262311307 (PMC12692008; doi:10.3390/ijms262311307)
Supplement: Supplementary file 1 [file ijms-26-11307-s001.zip › Figure S1. Scatter plots of the genetic association of remaining 21 metabolites on the risk of AML..pdf]

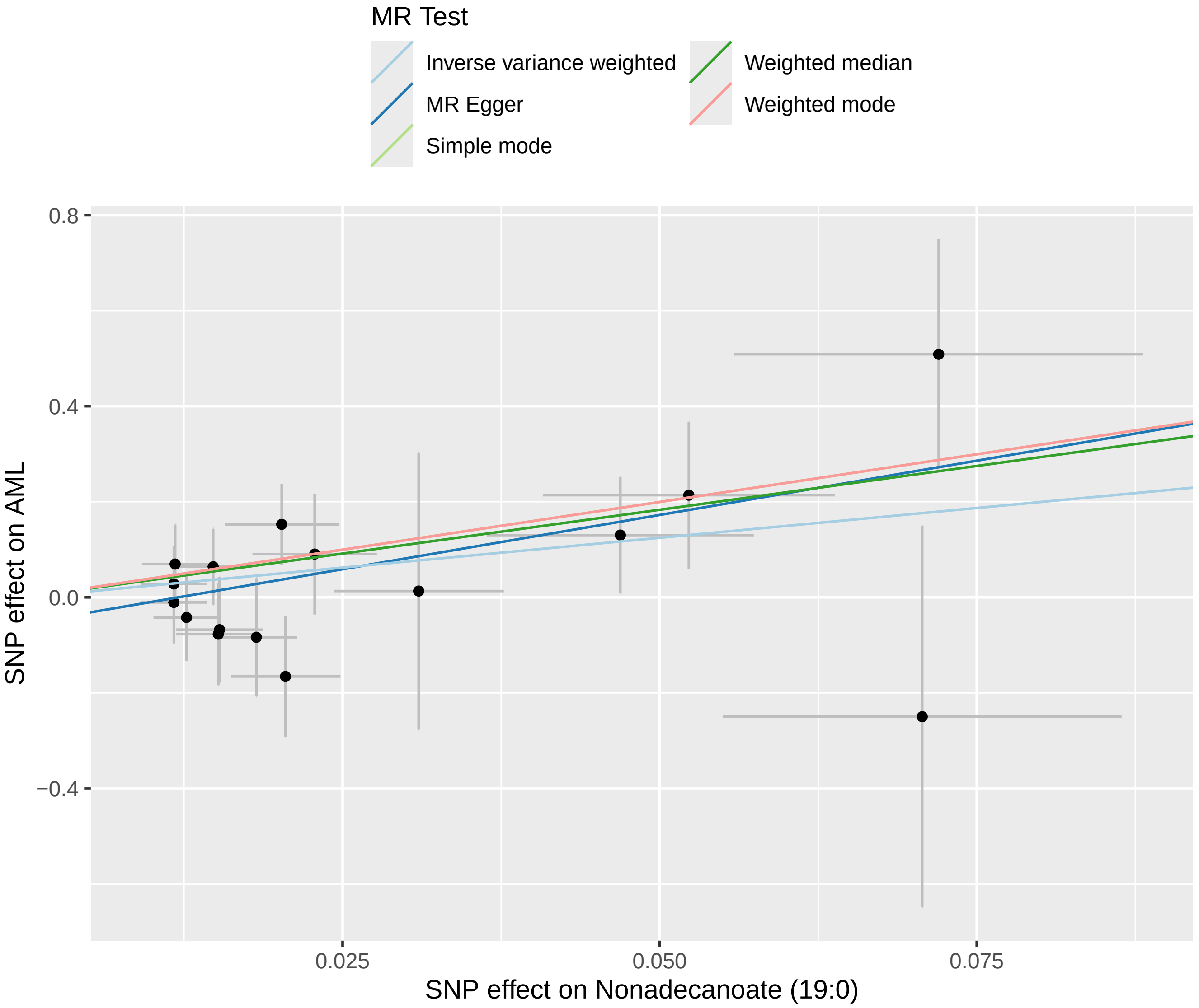
MR Test
Inverse variance weighted
MR Egger
Simple mode
Weighted median
Weighted mode
0.8
0.4
0.0
−0.4
SNP effect on AML
0.025
0.050
0.075
SNP effect on Nonadecanoate (19:0)

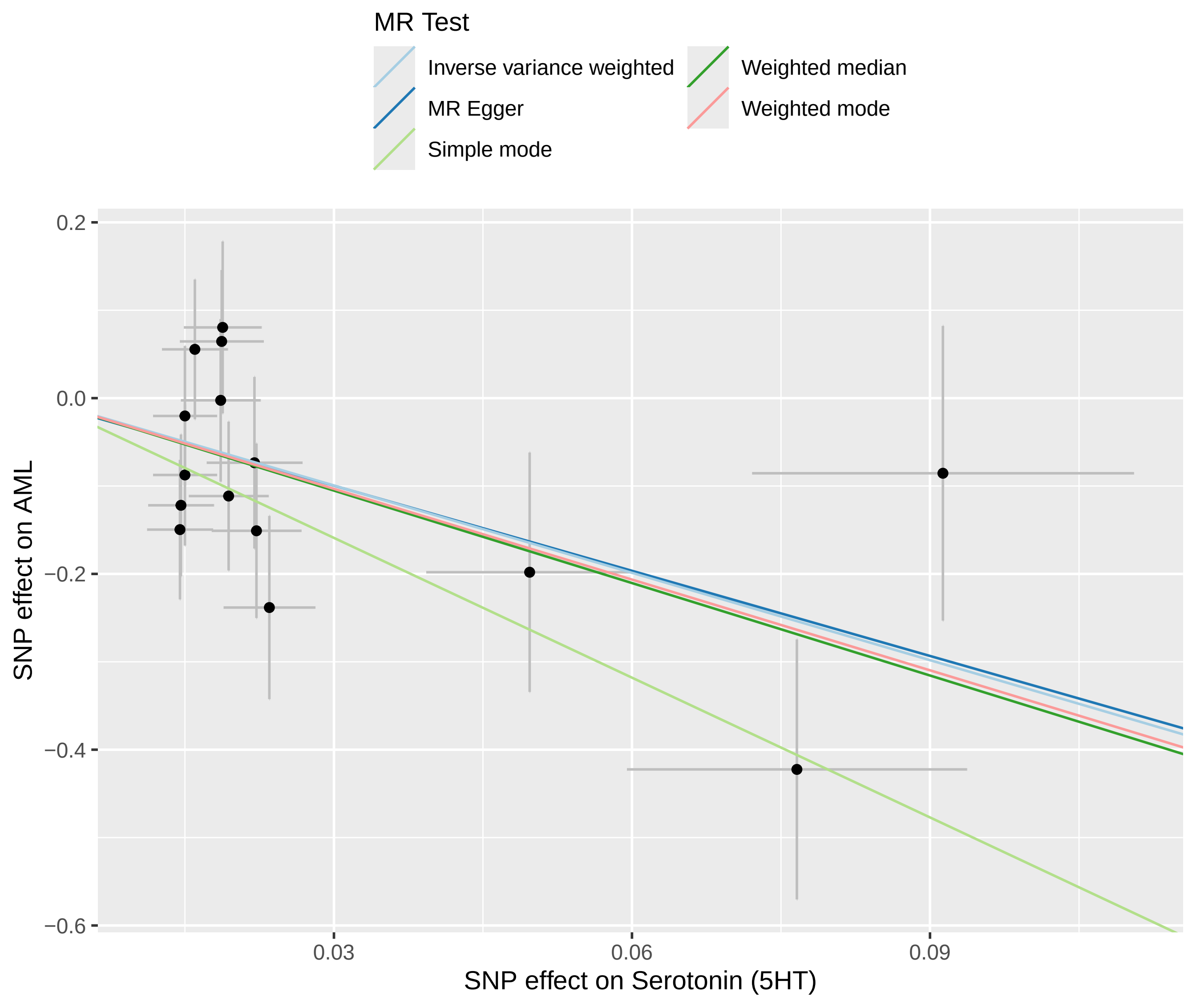

MR Test
Inverse variance weighted
MR Egger
Simple mode
Weighted median
Weighted mode
0.2
0.0
−0.2
−0.4
−0.6
0.03
0.06
0.09
SNP effect on AML
SNP effect on Serotonin (5HT)

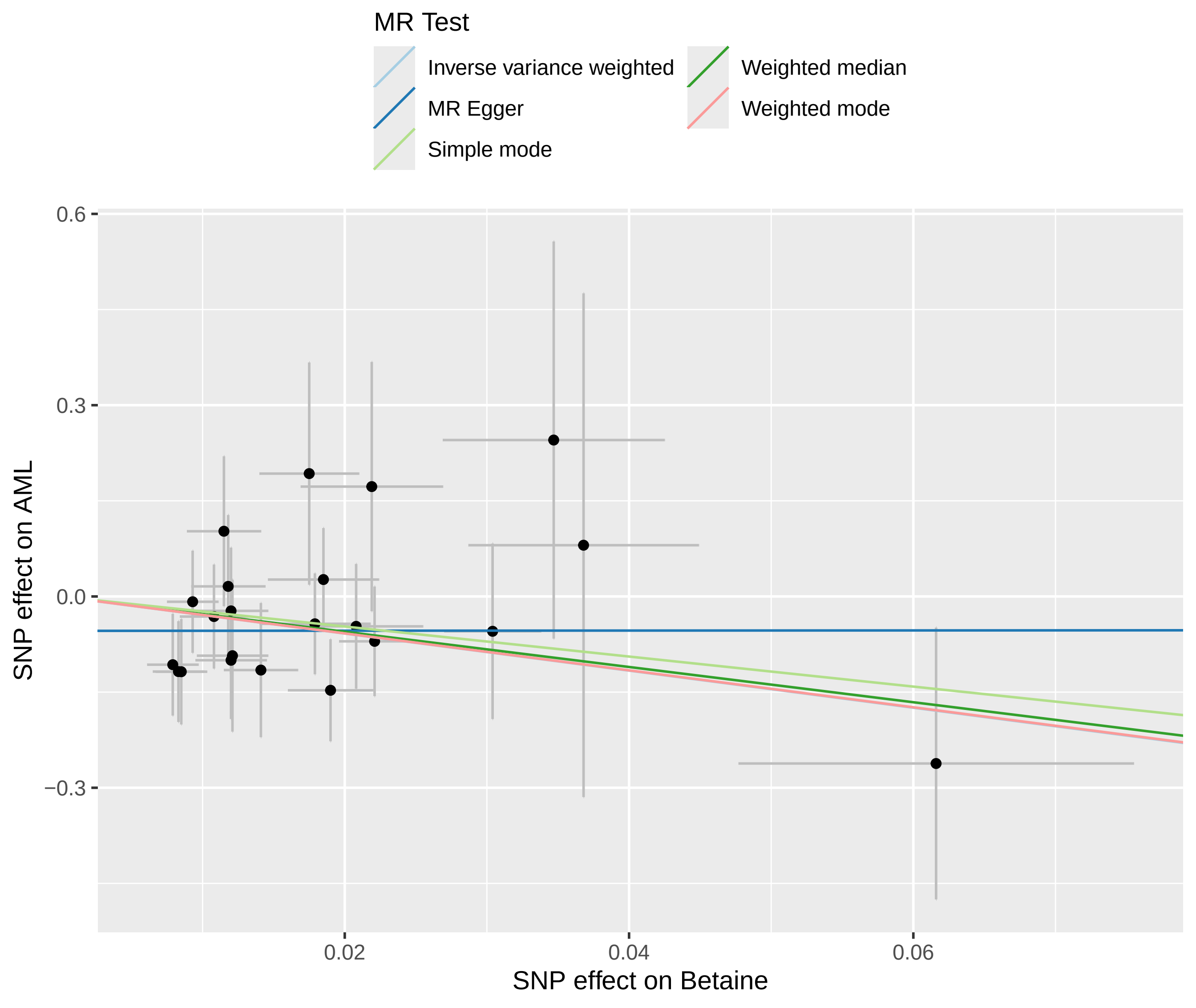
MR Test
Inverse variance weighted
MR Egger
Simple mode
Weighted median
Weighted mode
0.6
0.3
0.0
−0.3
SNP effect on AML
0.02
0.04
0.06
SNP effect on Betaine

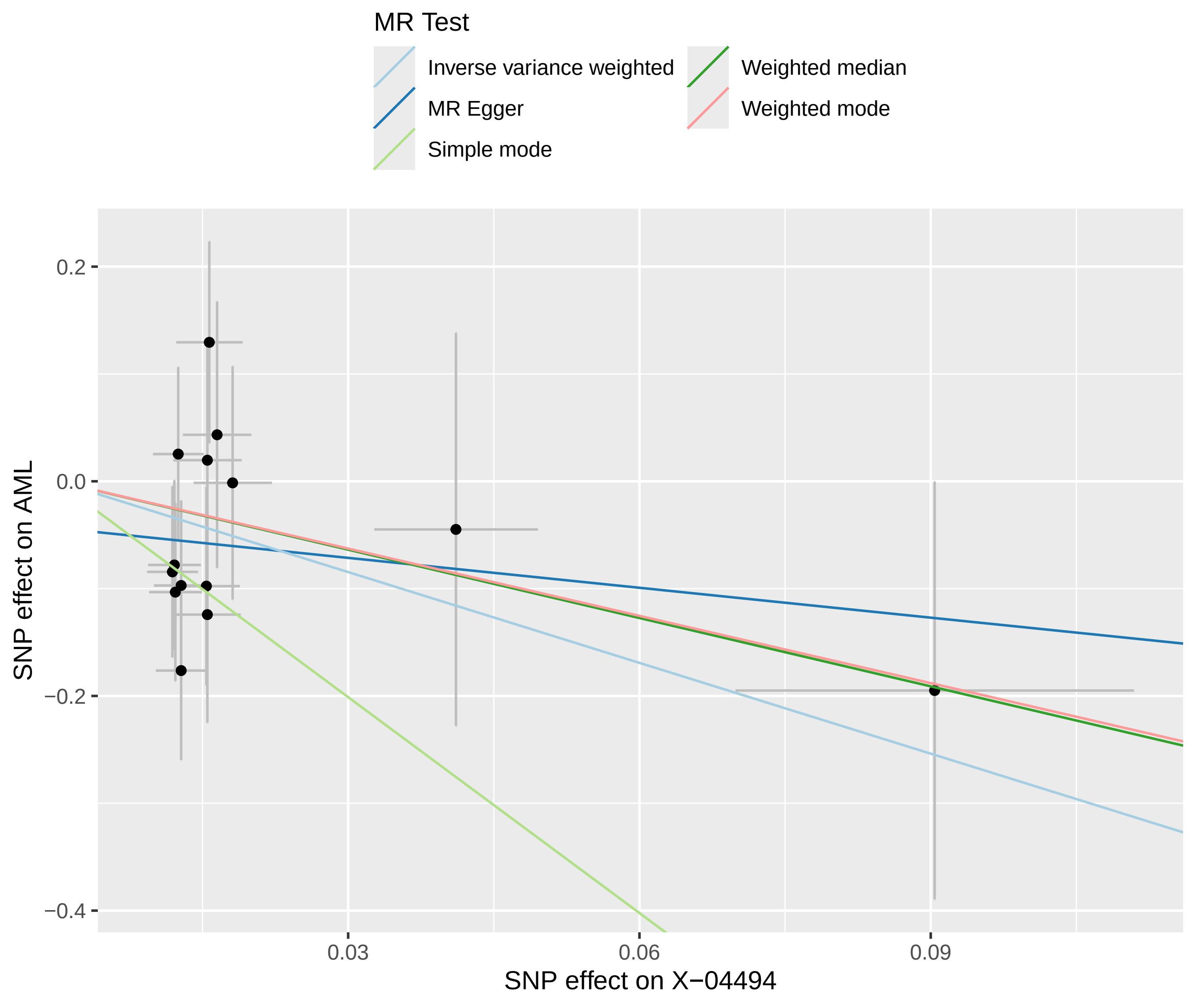
MR Test
Inverse variance weighted
MR Egger
Simple mode
Weighted median
Weighted mode
0.2
0.0
−0.2
−0.4
SNP effect on AML
0.03
0.06
0.09
SNP effect on X−04494

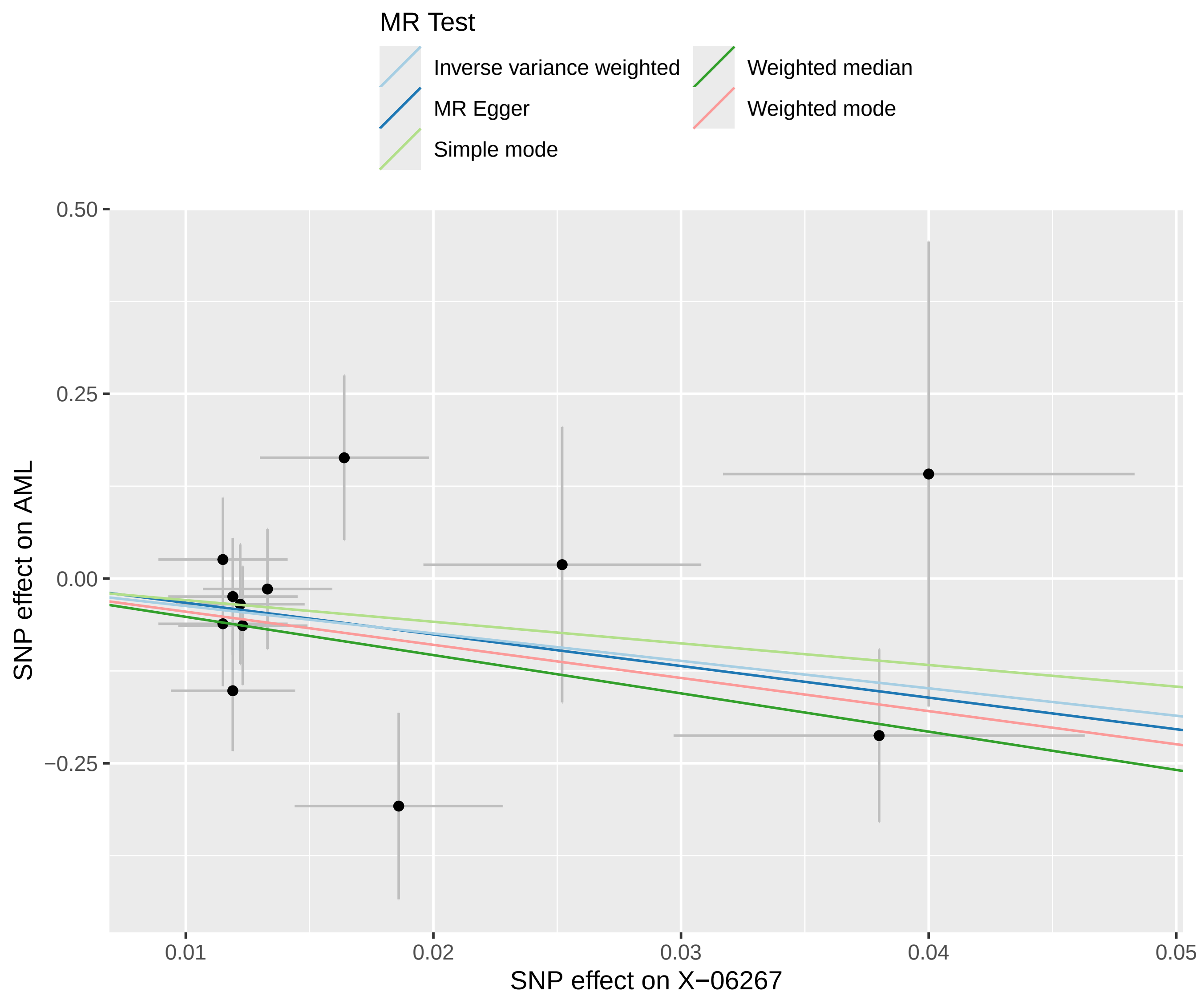
MR Test
Inverse variance weighted
MR Egger
Simple mode
Weighted median
Weighted mode
0.50
0.25
0.00
−0.25
SNP effect on AML
0.01
0.02
0.03
0.04
0.05
SNP effect on X−06267

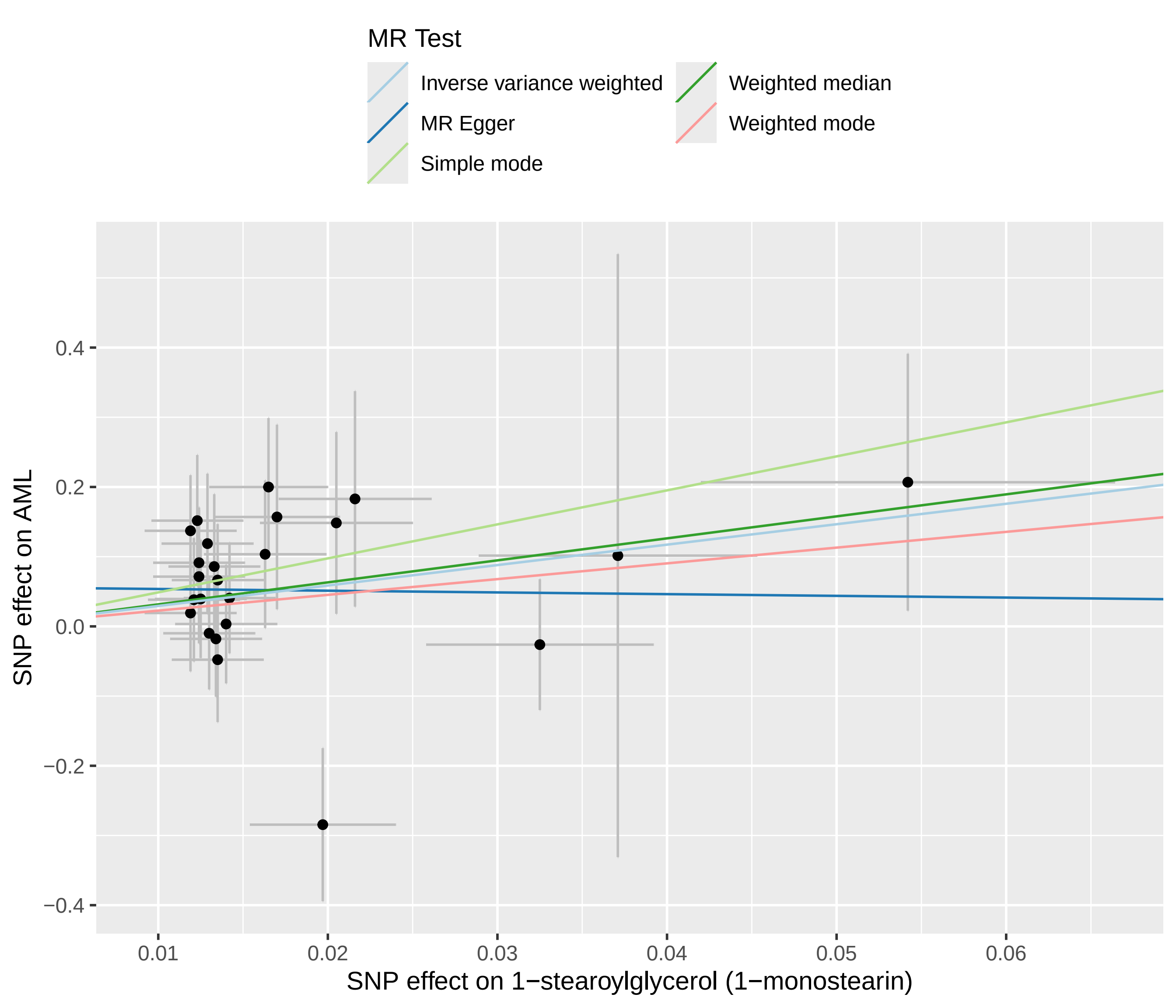
MR Test
Inverse variance weighted
MR Egger
Simple mode
Weighted median
Weighted mode
SNP effect on AML
0.4
0.2
0.0
−0.2
−0.4
0.01
0.02
0.03
0.04
0.05
0.06
SNP effect on 1−stearoylglycerol (1−monostearin)

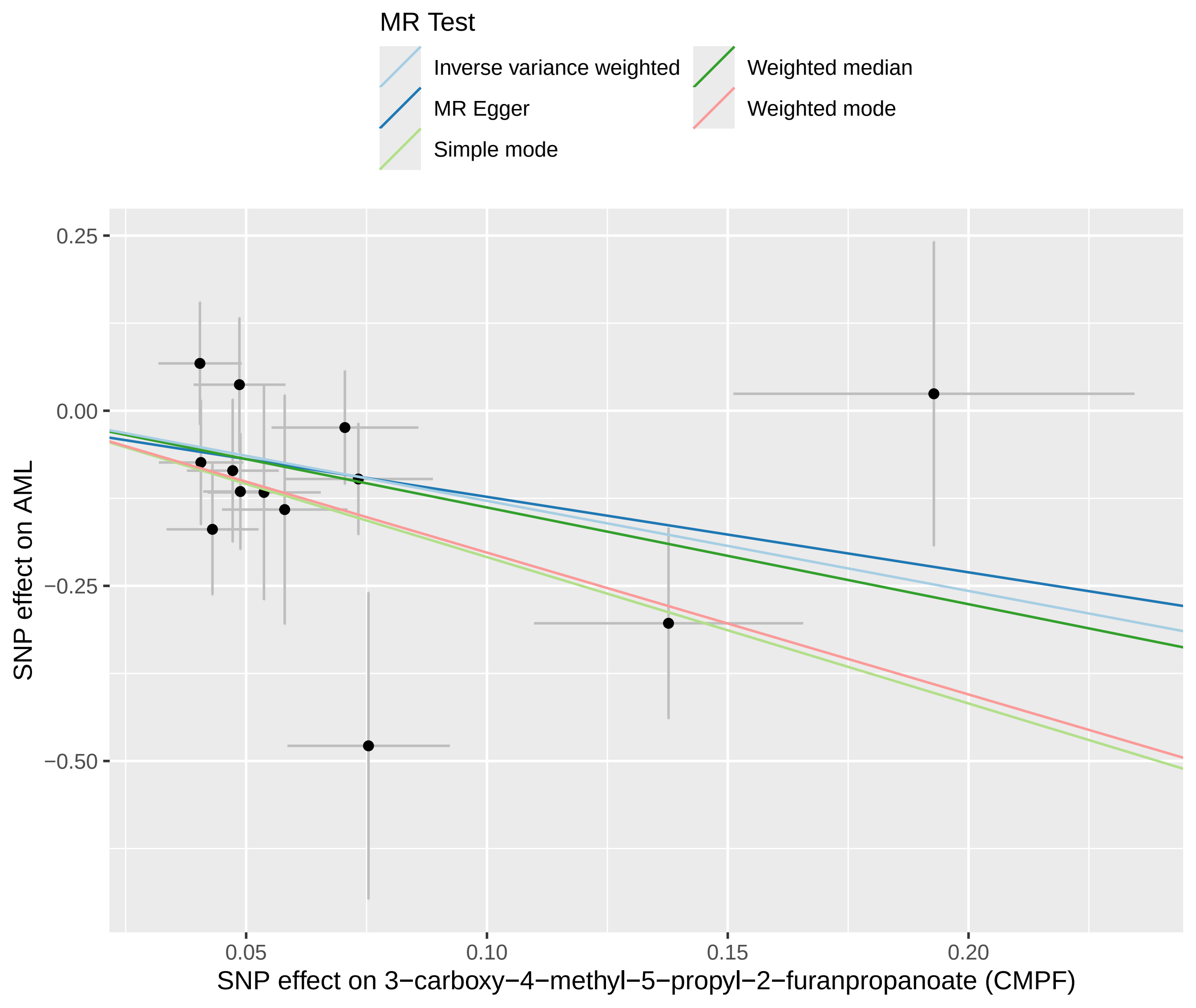

MR Test
Inverse variance weighted
MR Egger
Simple mode
Weighted median
Weighted mode
0.25
0.00
−0.25
−0.50
SNP effect on AML
0.05
0.10
0.15
0.20
SNP effect on 3−carboxy−4−methyl−5−propyl−2−furanpropanoate (CMPF)

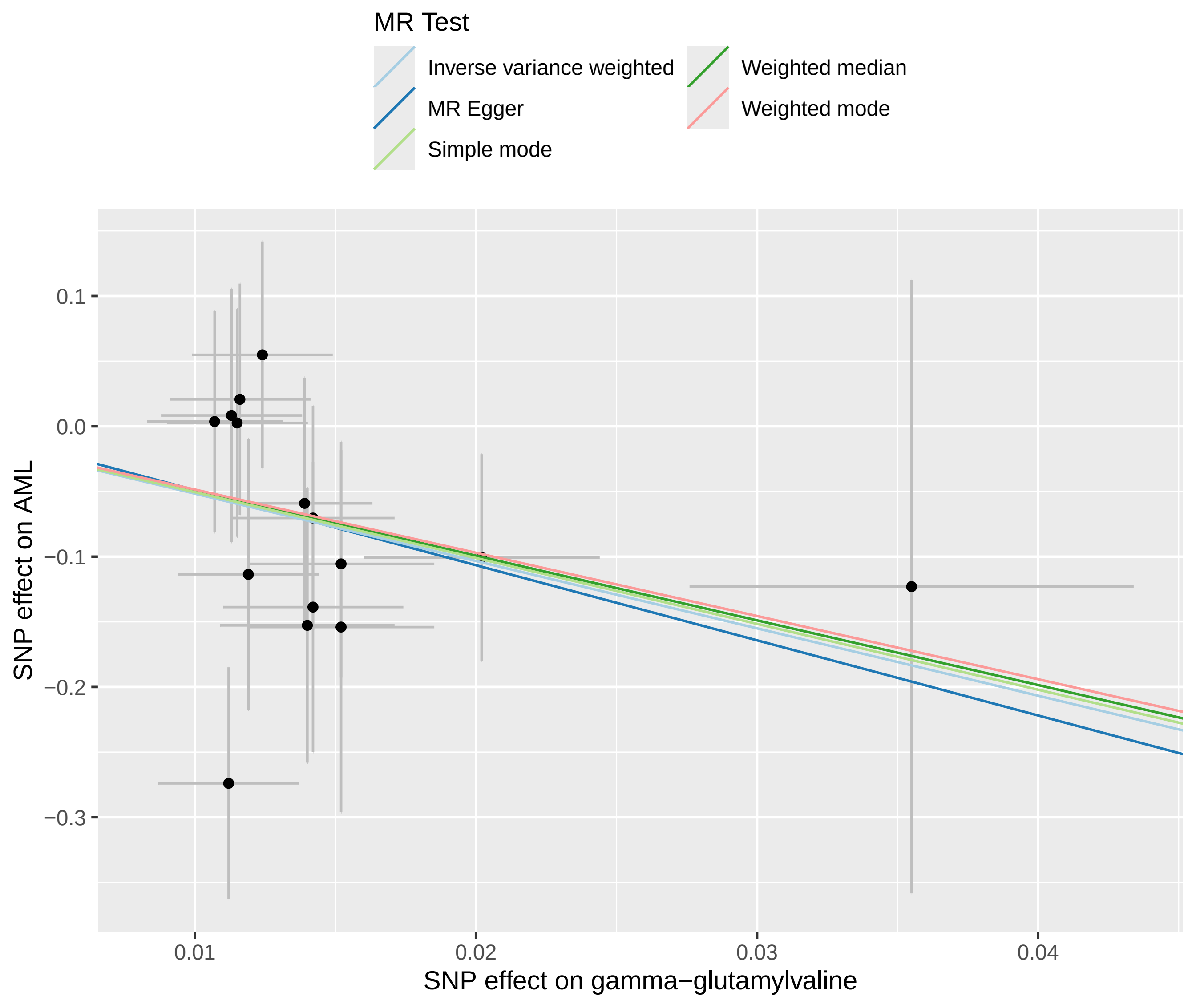
MR Test
Inverse variance weighted
MR Egger
Simple mode
Weighted median
Weighted mode
0.1
0.0
−0.1
−0.2
−0.3
SNP effect on AML
0.01
0.02
0.03
0.04
SNP effect on gamma−glutamylvaline

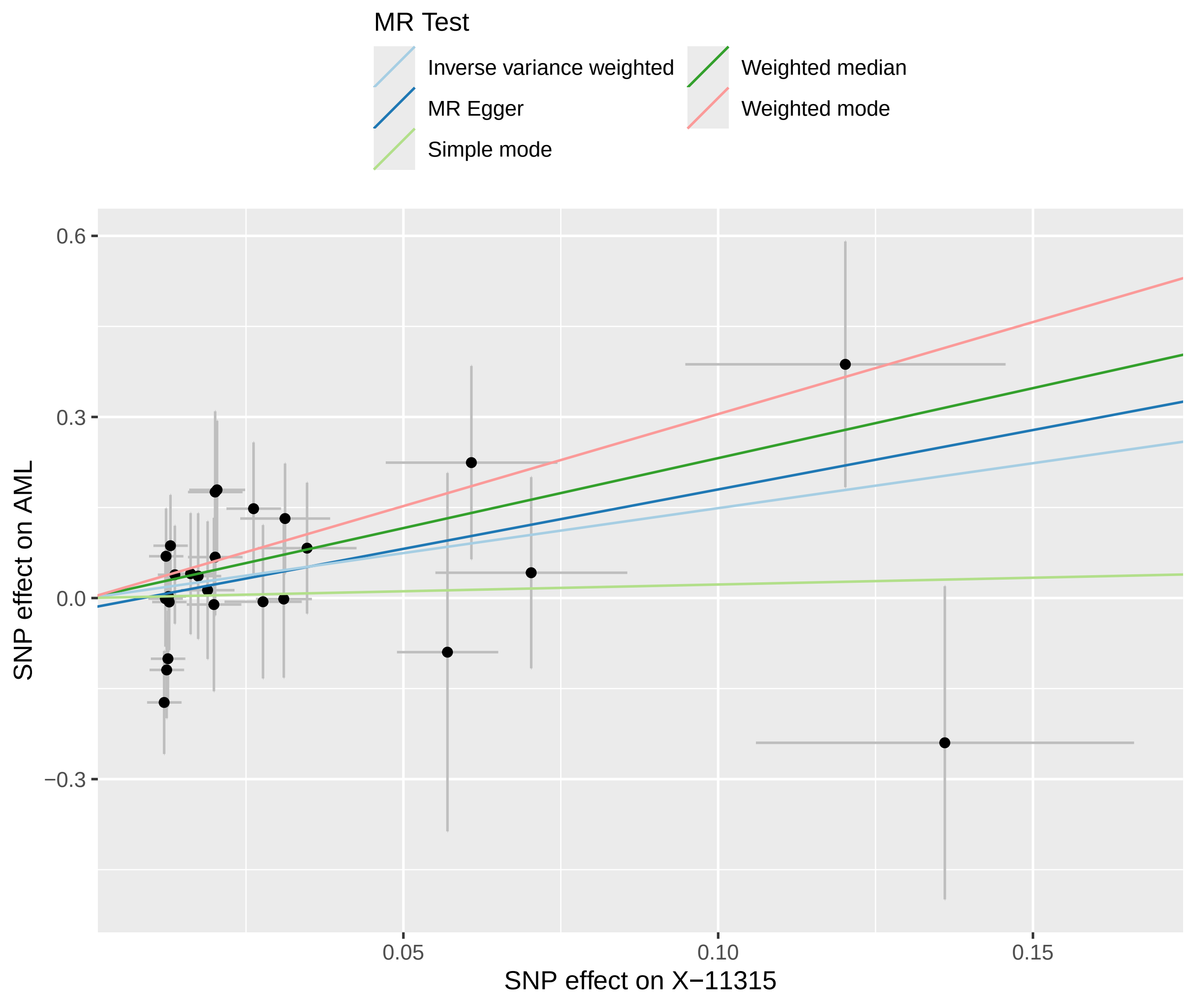
MR Test
Inverse variance weighted
MR Egger
Simple mode
Weighted median
Weighted mode
0.6
0.3
0.0
−0.3
SNP effect on AML
0.05
0.10
0.15
SNP effect on X−11315

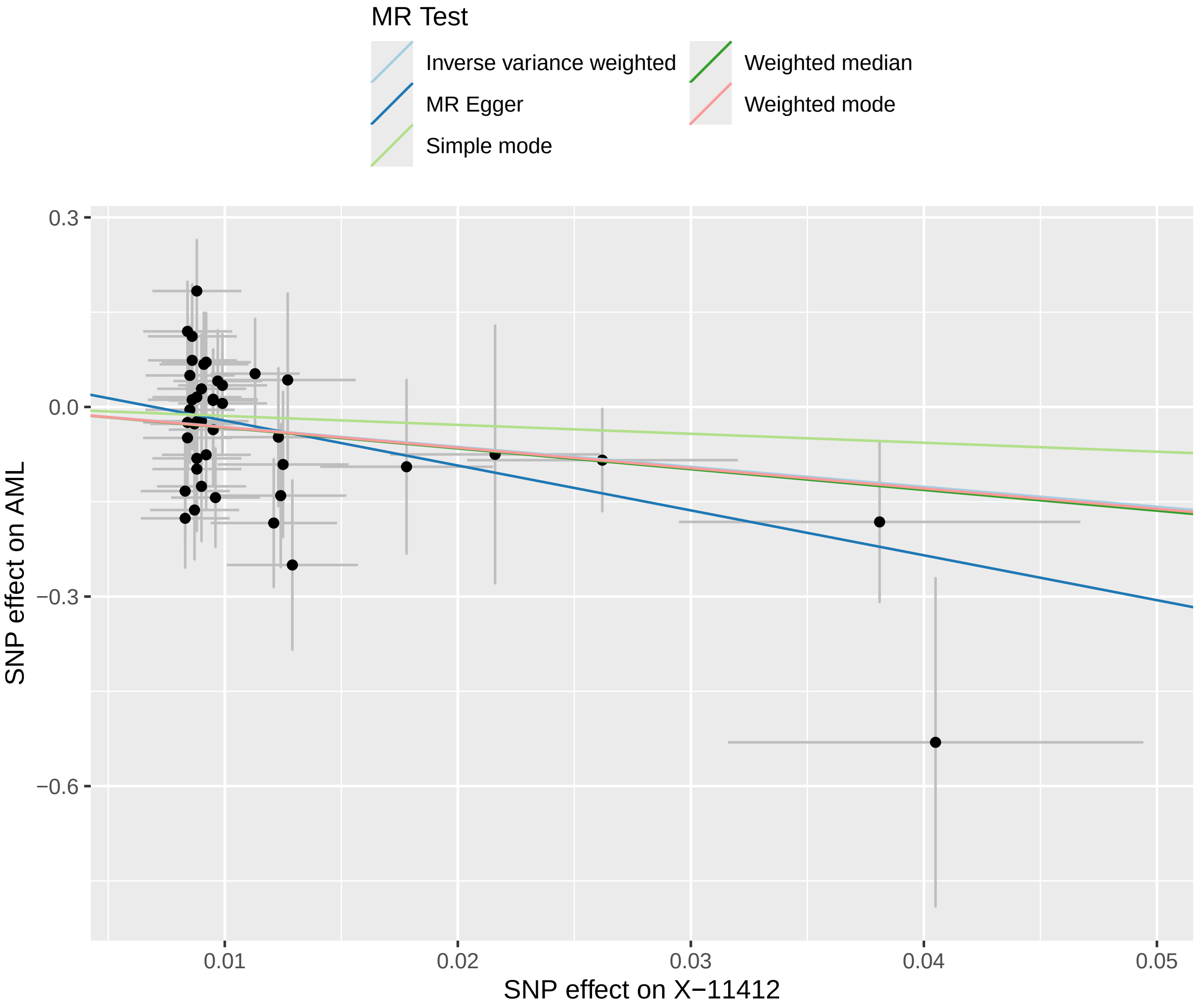
MR Test
Inverse variance weighted
MR Egger
Simple mode
Weighted median
Weighted mode
0.3
0.0
−0.3
−0.6
0.01
0.02
0.03
0.04
0.05
SNP effect on AML
SNP effect on X−11412

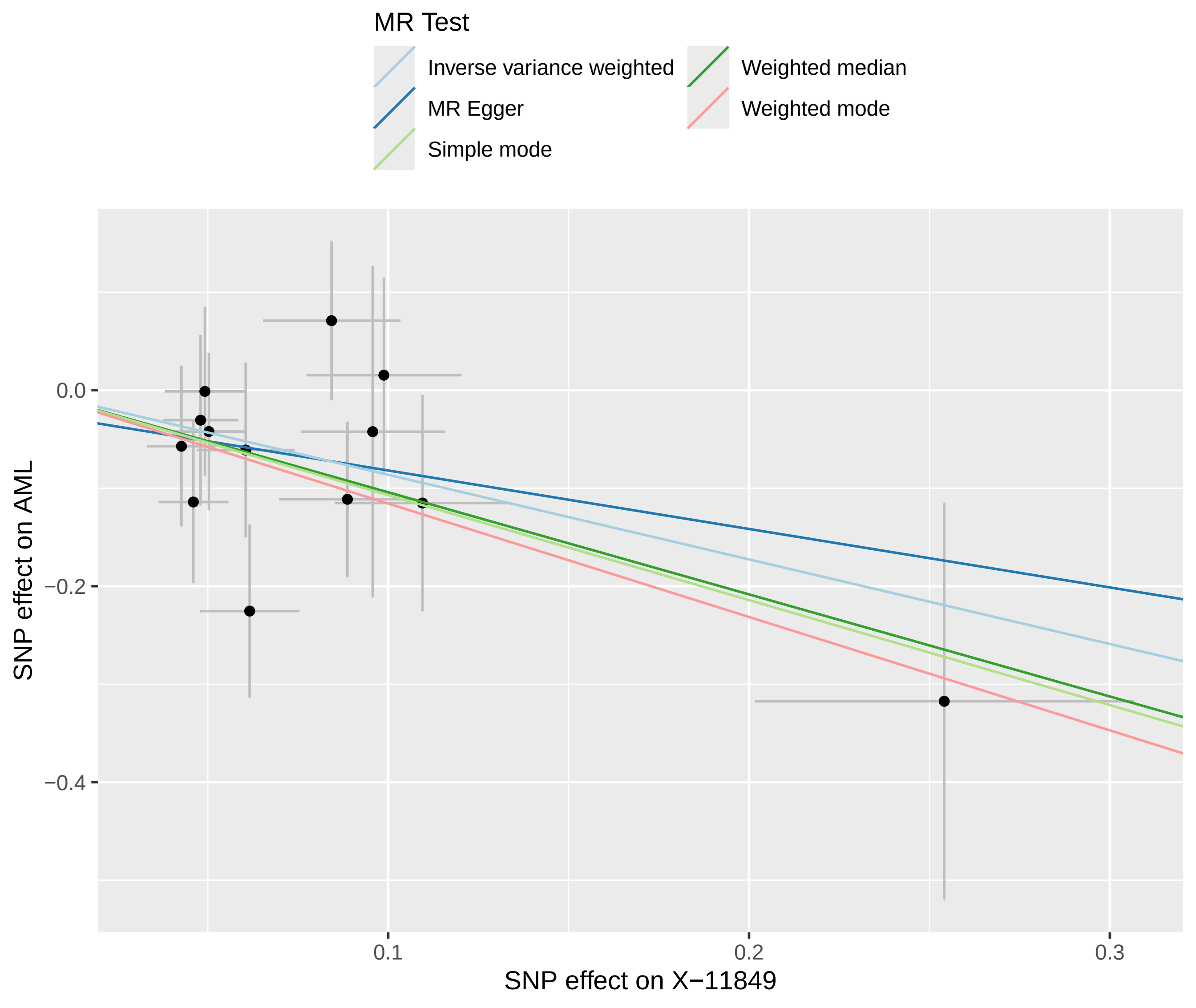
MR Test
Inverse variance weighted
MR Egger
Simple mode
Weighted median
Weighted mode
0.0
−0.2
−0.4
0.1
0.2
0.3
SNP effect on AML
SNP effect on X−11849

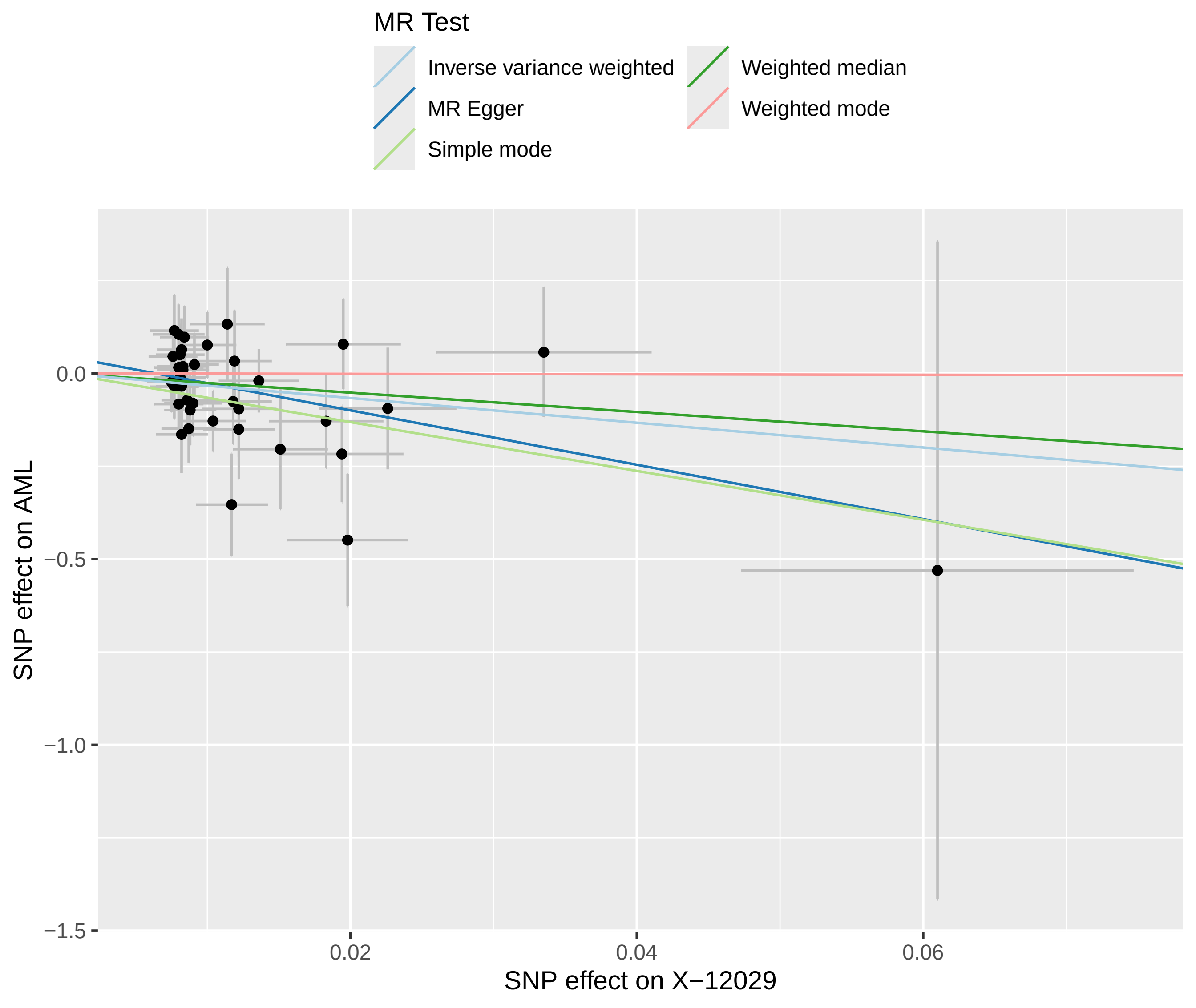
MR Test
Inverse variance weighted
MR Egger
Simple mode
Weighted median
Weighted mode
SNP effect on AML
0.0
−0.5
−1.0
−1.5
0.02
0.04
0.06
SNP effect on X−12029

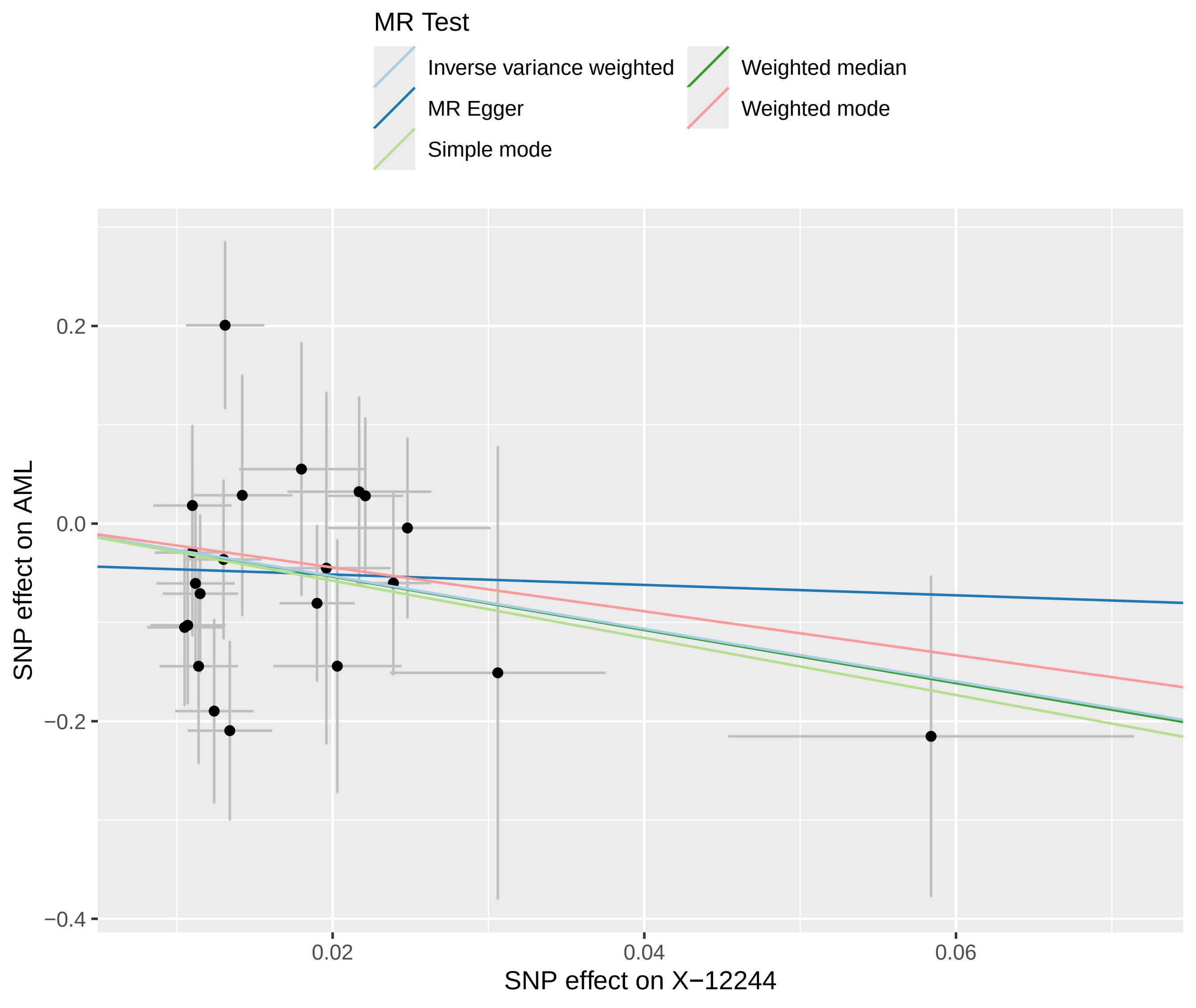

MR Test
Inverse variance weighted
MR Egger
Simple mode
Weighted median
Weighted mode
SNP effect on AML
0.2
0.0
−0.2
−0.4
0.02
0.04
0.06
SNP effect on X−12244

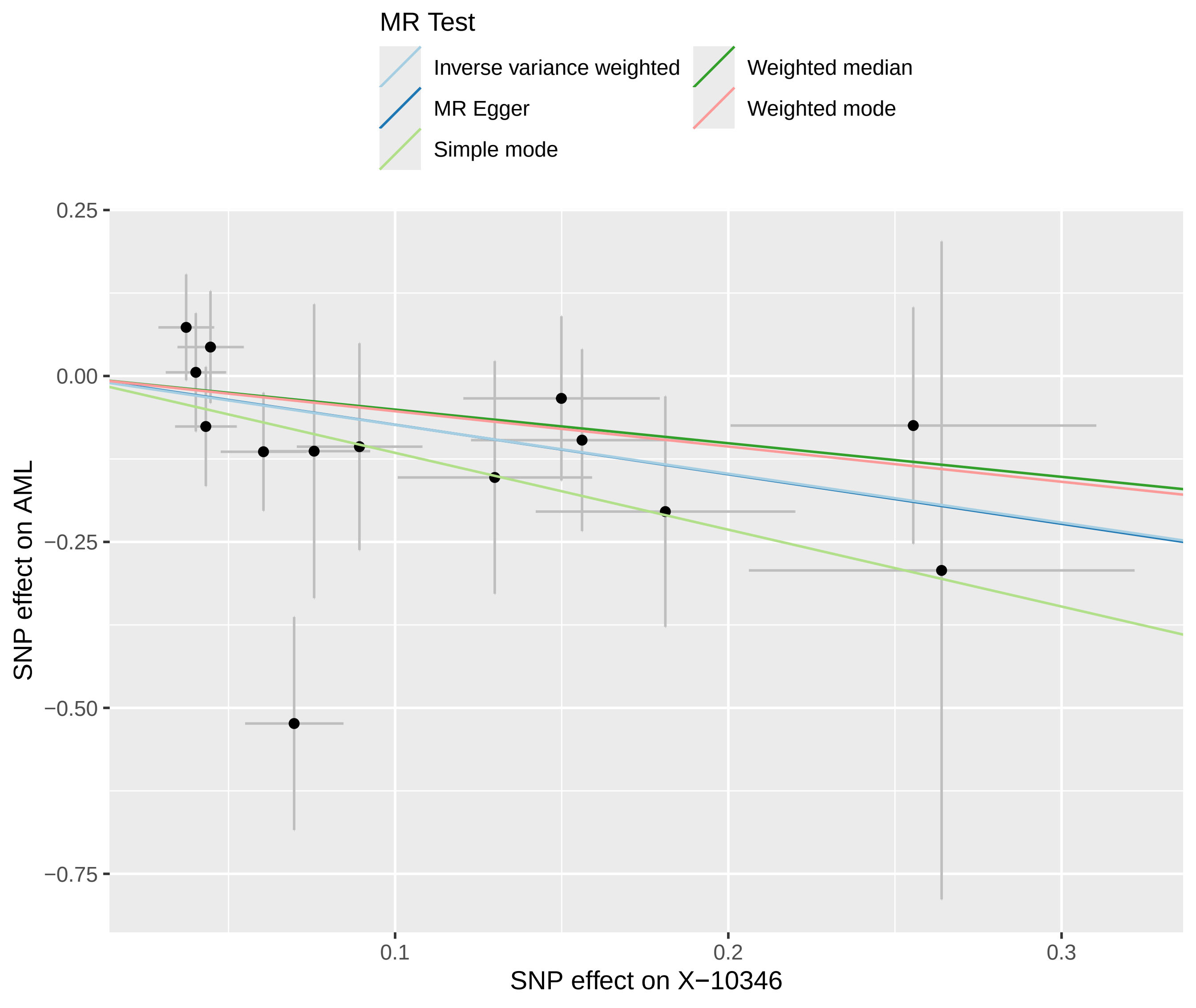
MR Test
Inverse variance weighted
MR Egger
Simple mode
Weighted median
Weighted mode
0.25
0.00
−0.25
−0.50
−0.75
SNP effect on AML
0.1
0.2
0.3
SNP effect on X−10346

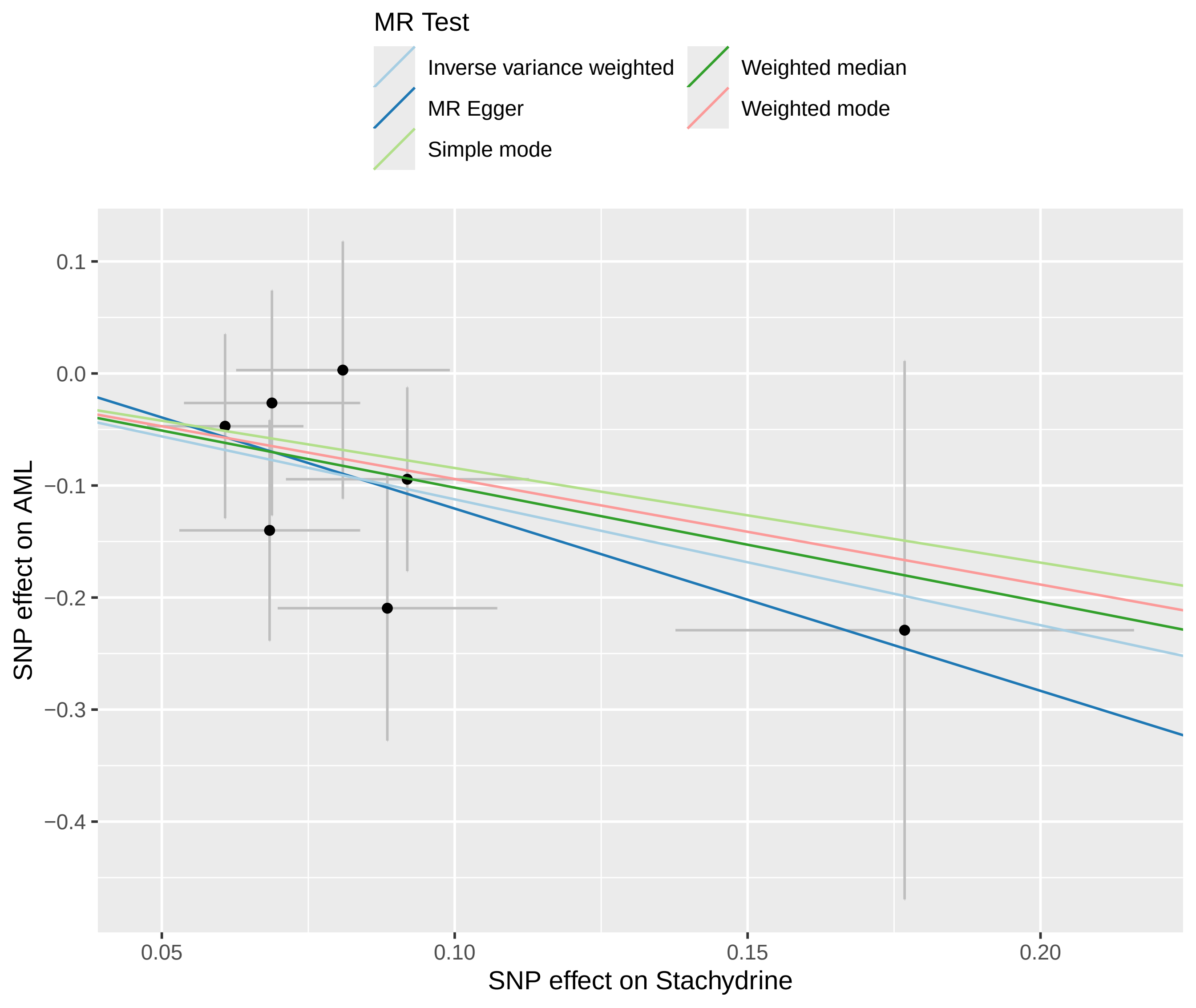

MR Test
Inverse variance weighted
MR Egger
Simple mode
Weighted median
Weighted mode
0.1
0.0
−0.1
−0.2
−0.3
−0.4
SNP effect on AML
0.05
0.10
0.15
0.20
SNP effect on Stachydrine

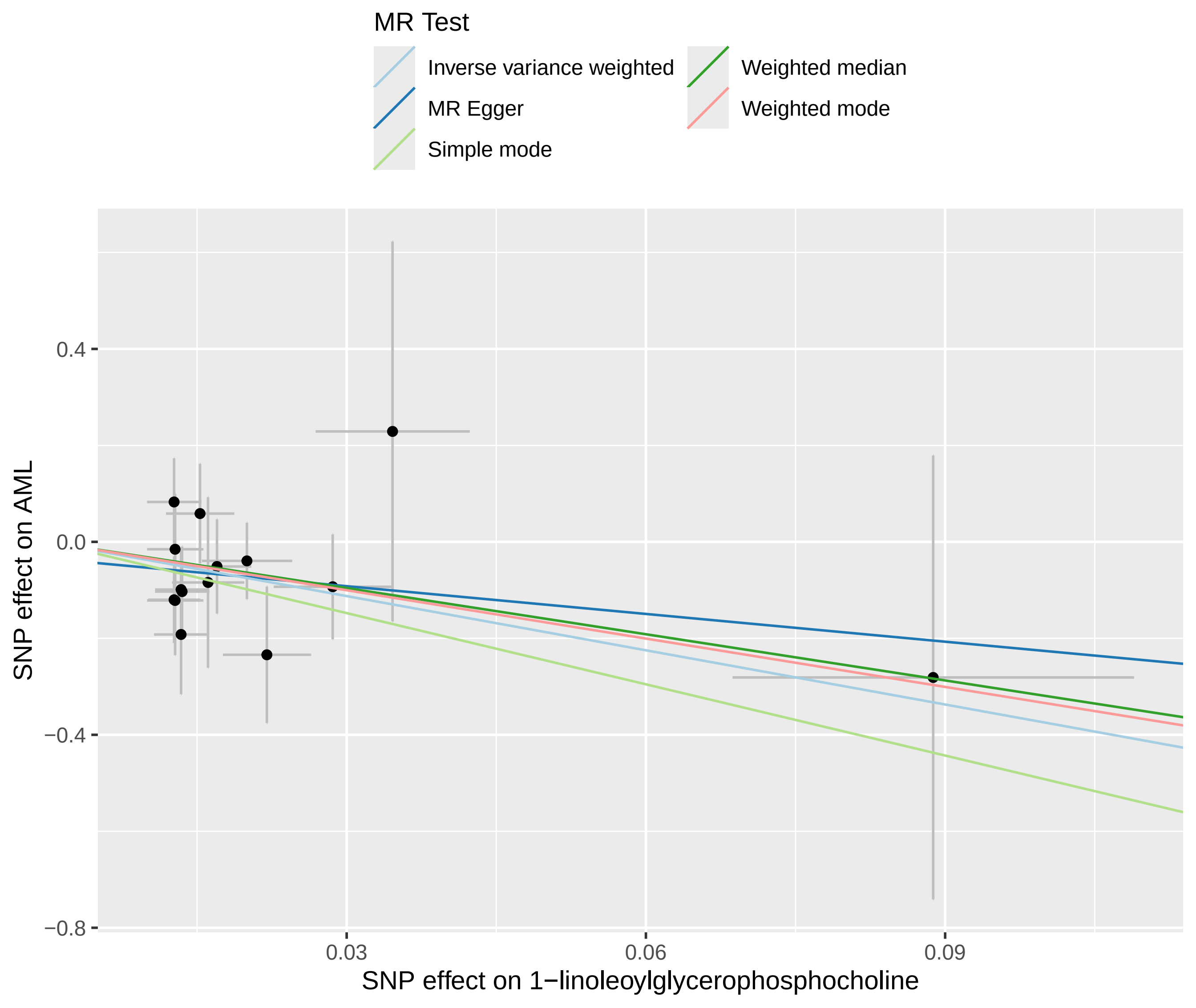
MR Test
Inverse variance weighted
MR Egger
Simple mode
Weighted median
Weighted mode
SNP effect on AML
0.4
0.0
−0.4
−0.8
0.03
0.06
0.09
SNP effect on 1−linoleoylglycerophosphocholine

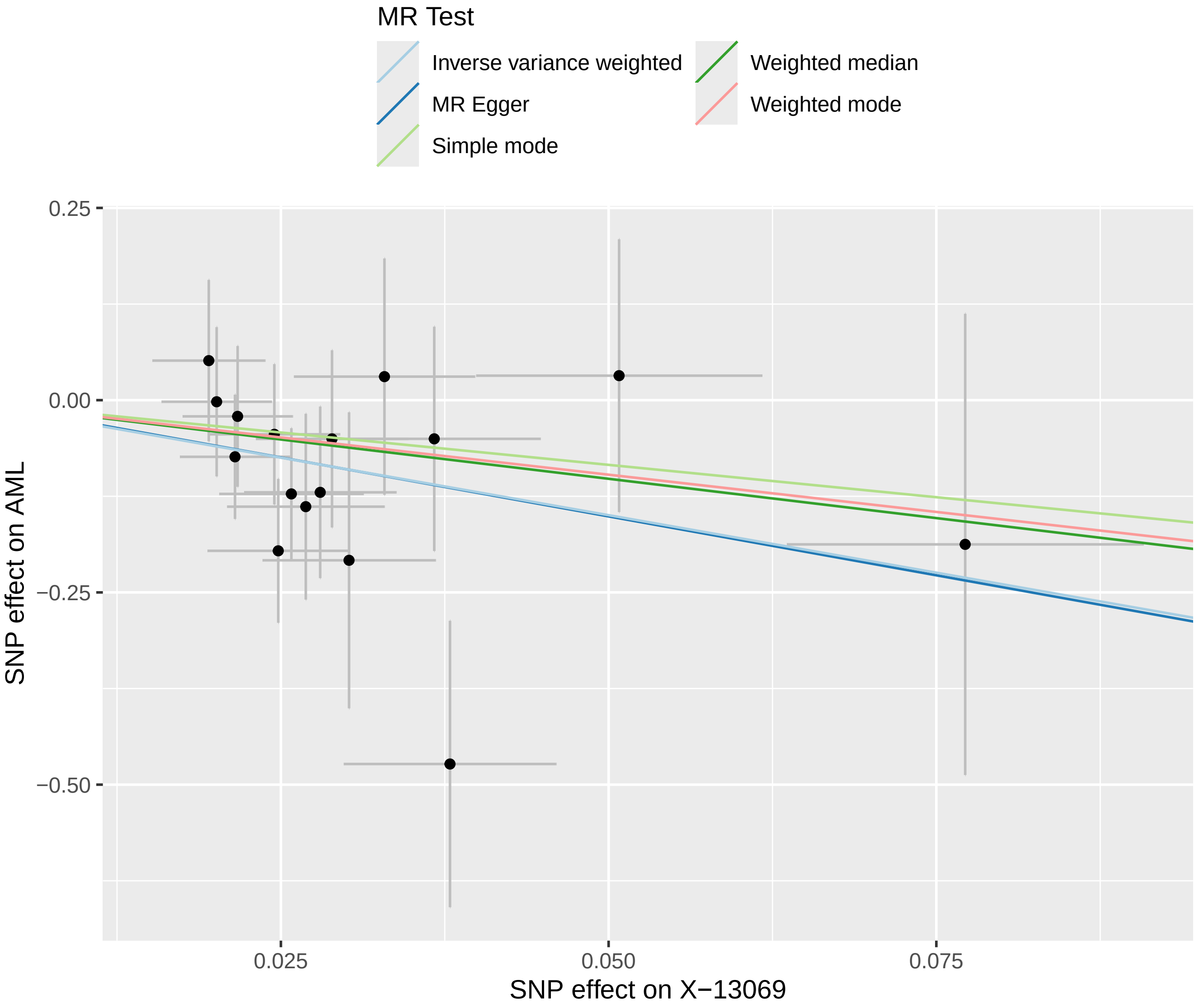
MR Test
Inverse variance weighted
MR Egger
Simple mode
Weighted median
Weighted mode
0.25
0.00
−0.25
−0.50
0.025
0.050
0.075
SNP effect on AML
SNP effect on X−13069

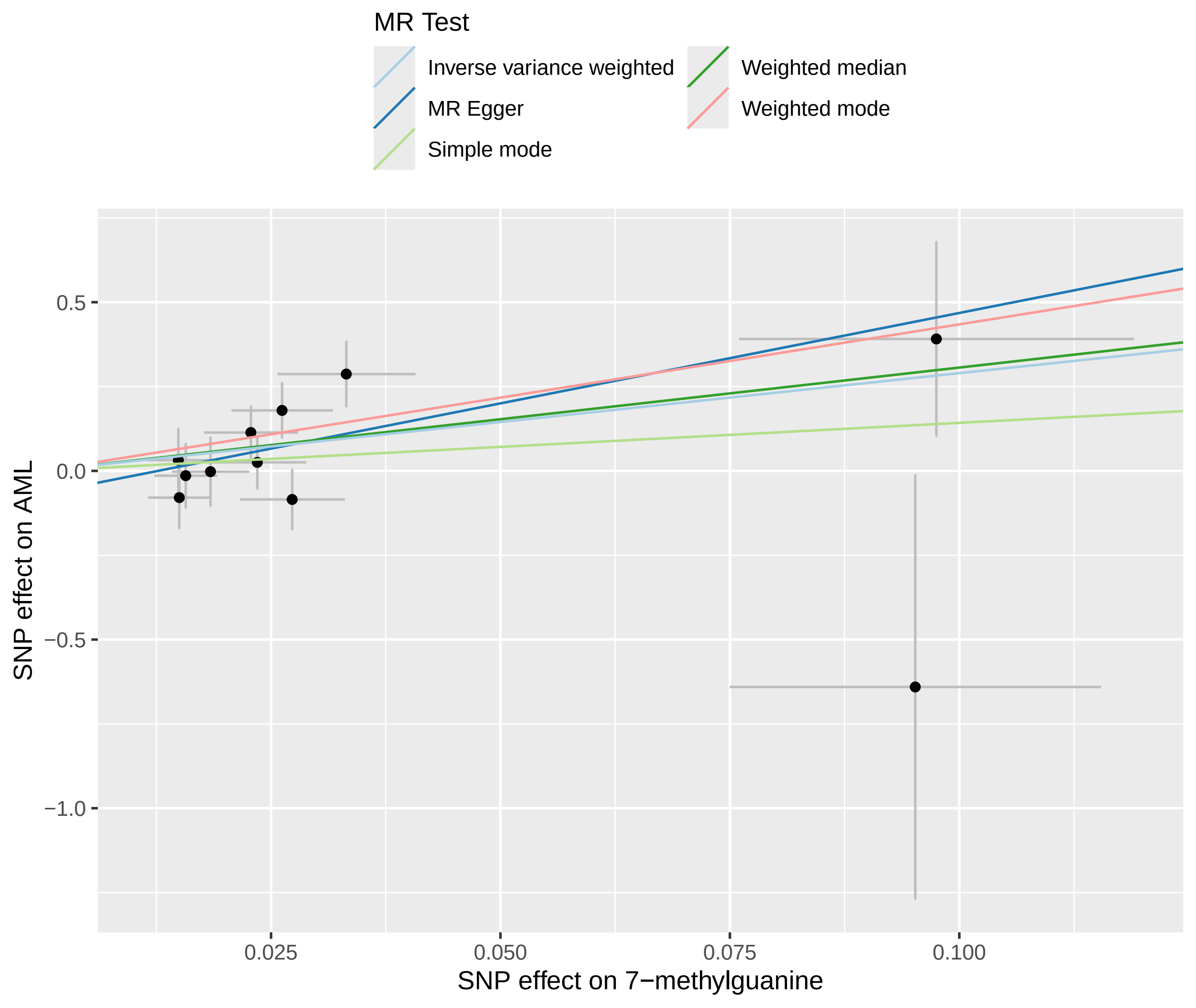
MR Test
Inverse variance weighted
MR Egger
Simple mode
Weighted median
Weighted mode
0.5
0.0
−0.5
−1.0
0.025
0.050
0.075
0.100
SNP effect on AML
SNP effect on 7−methylguanine

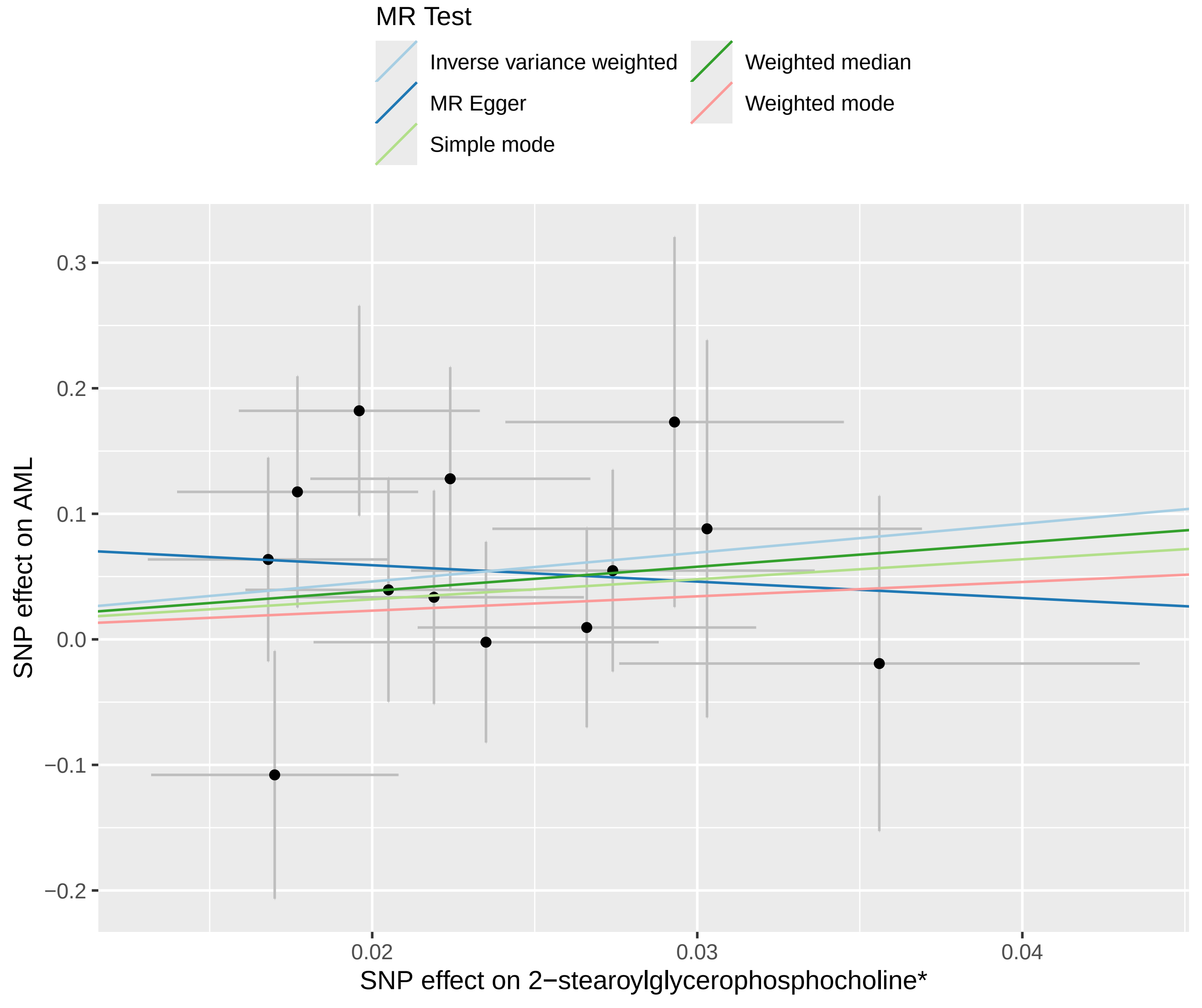
MR Test
Inverse variance weighted
MR Egger
Simple mode
Weighted median
Weighted mode
0.3
0.2
0.1
0.0
−0.1
−0.2
SNP effect on AML
0.02
0.03
0.04
SNP effect on 2−stearoylglycerophosphocholine*

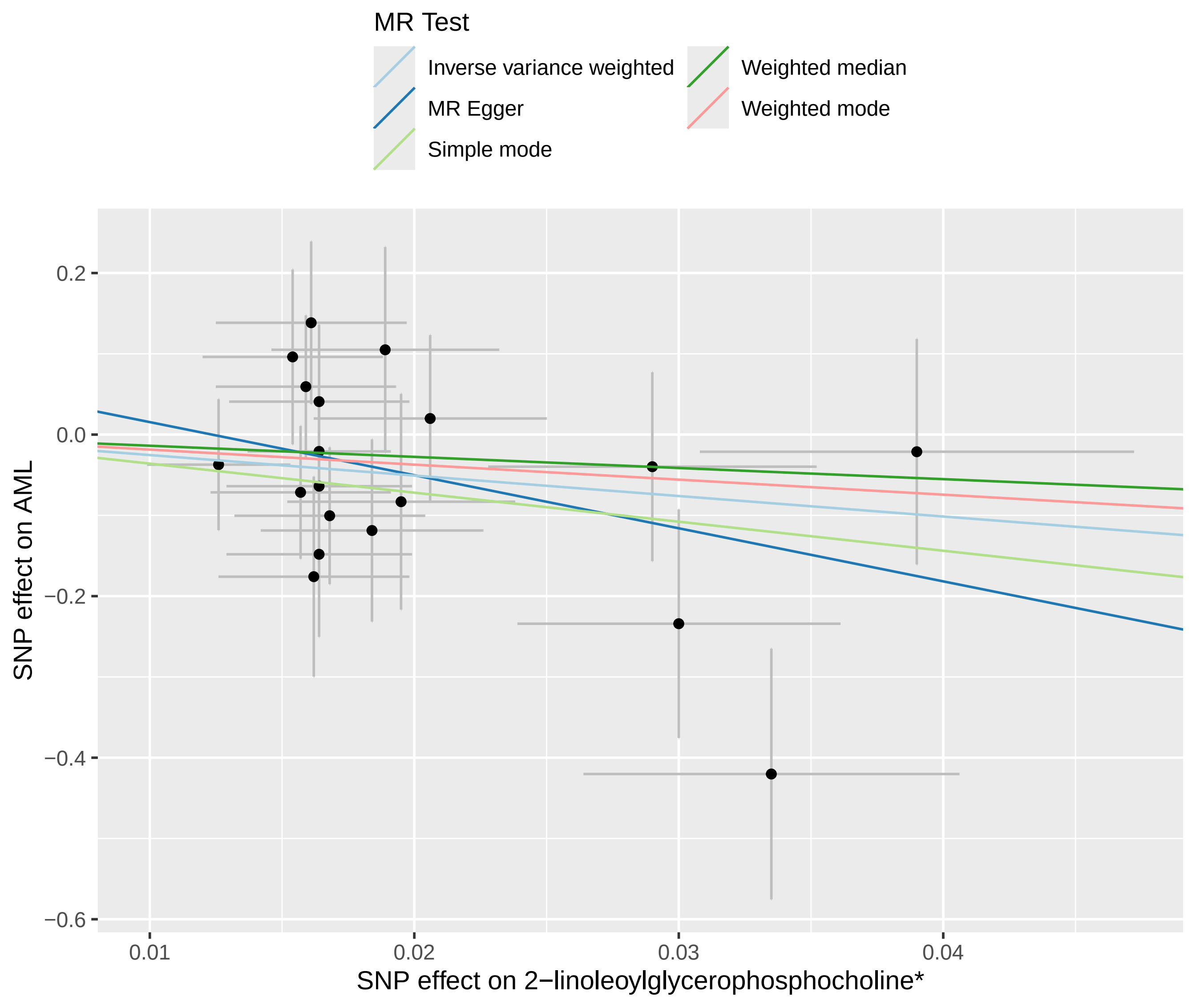
MR Test
Inverse variance weighted
MR Egger
Simple mode
Weighted median
Weighted mode
0.2
0.0
−0.2
−0.4
−0.6
SNP effect on AML
0.01
0.02
0.03
0.04
SNP effect on 2−linoleoylglycerophosphocholine*

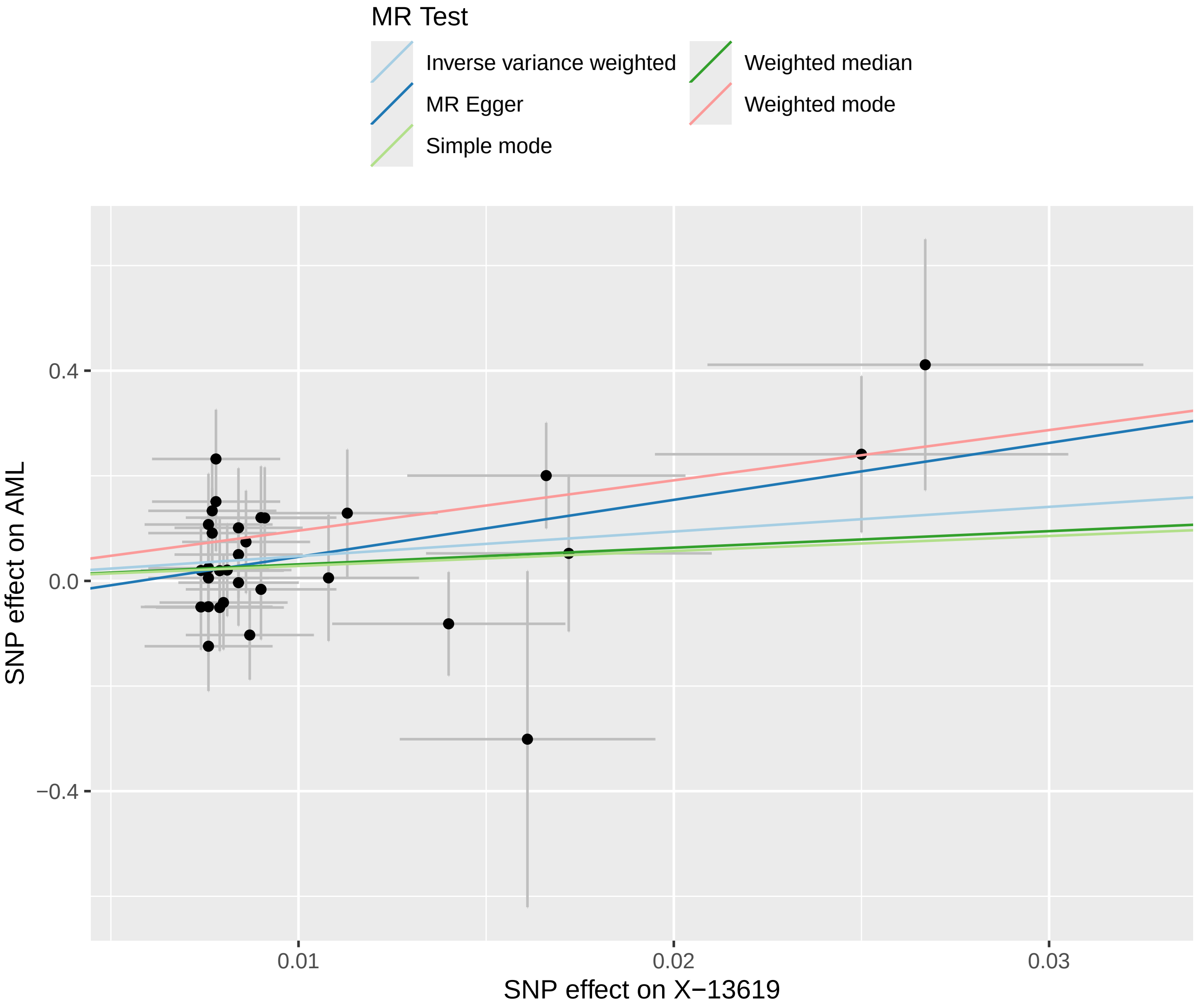
MR Test
Inverse variance weighted
MR Egger
Simple mode
Weighted median
Weighted mode
SNP effect on AML
0.4
0.0
−0.4
0.01
0.02
0.03
SNP effect on X−13619
