# Supplementary material for: Bidirectional Mendelian Randomization and Multi-Omics Uncover Causal Serum Metabolites and Neuro-Related Mechanistic Pathways in Acute Myeloid Leukemia
Source: Int J Mol Sci. 2025 Nov 22;26(23):11307. doi: 10.3390/ijms262311307 (PMC12692008; doi:10.3390/ijms262311307)
Supplement: Supplementary file 1 [file ijms-26-11307-s001.zip › Figure S2. Funnel plots for 23 potential metabolites on AML..pdf]

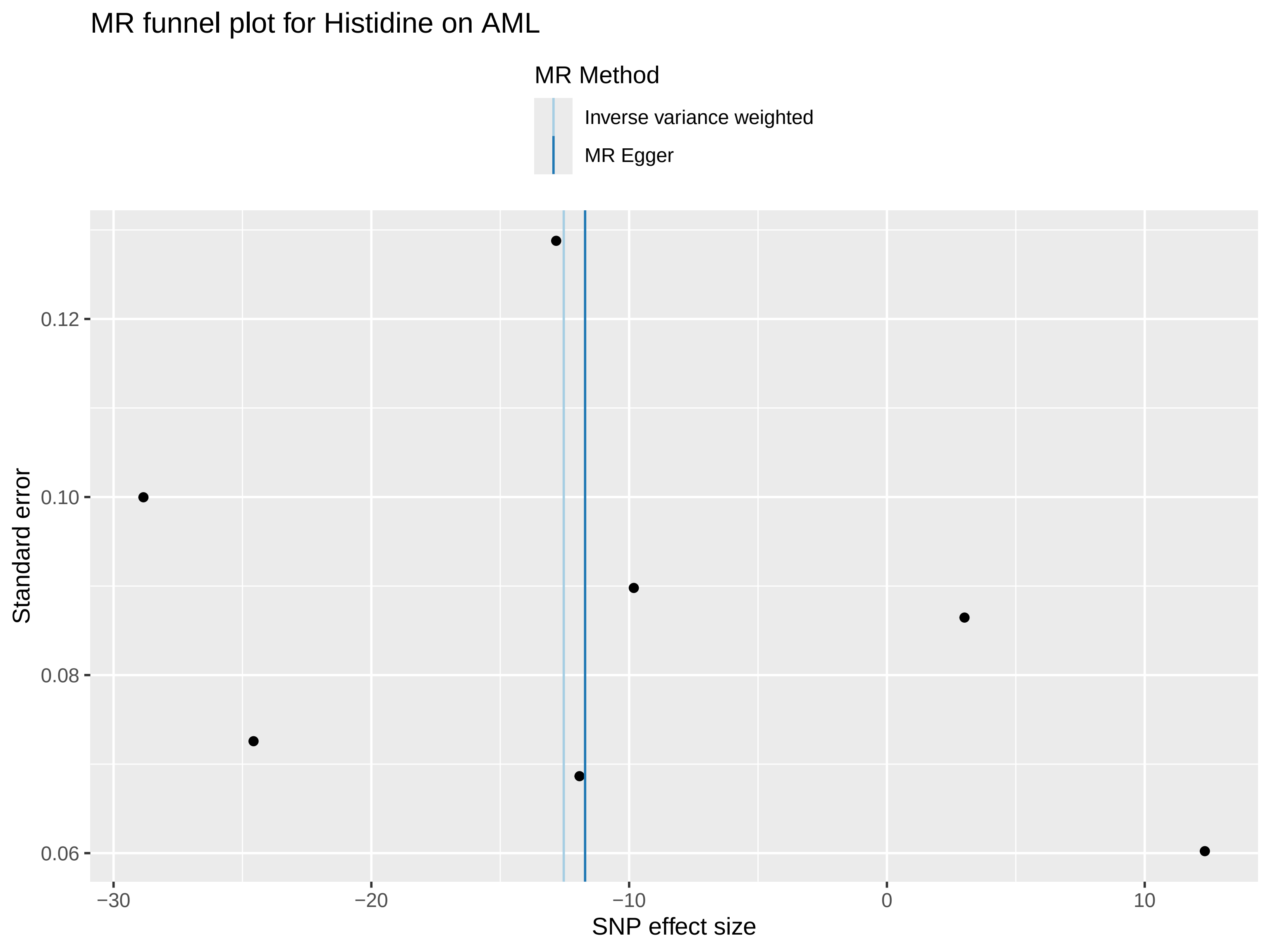

MR funnel plot for Histidine on AML
MR Method
Inverse variance weighted
MR Egger
Standard error
0.12
0.10
0.08
0.06
−30
−20
−10
0
10
SNP effect size

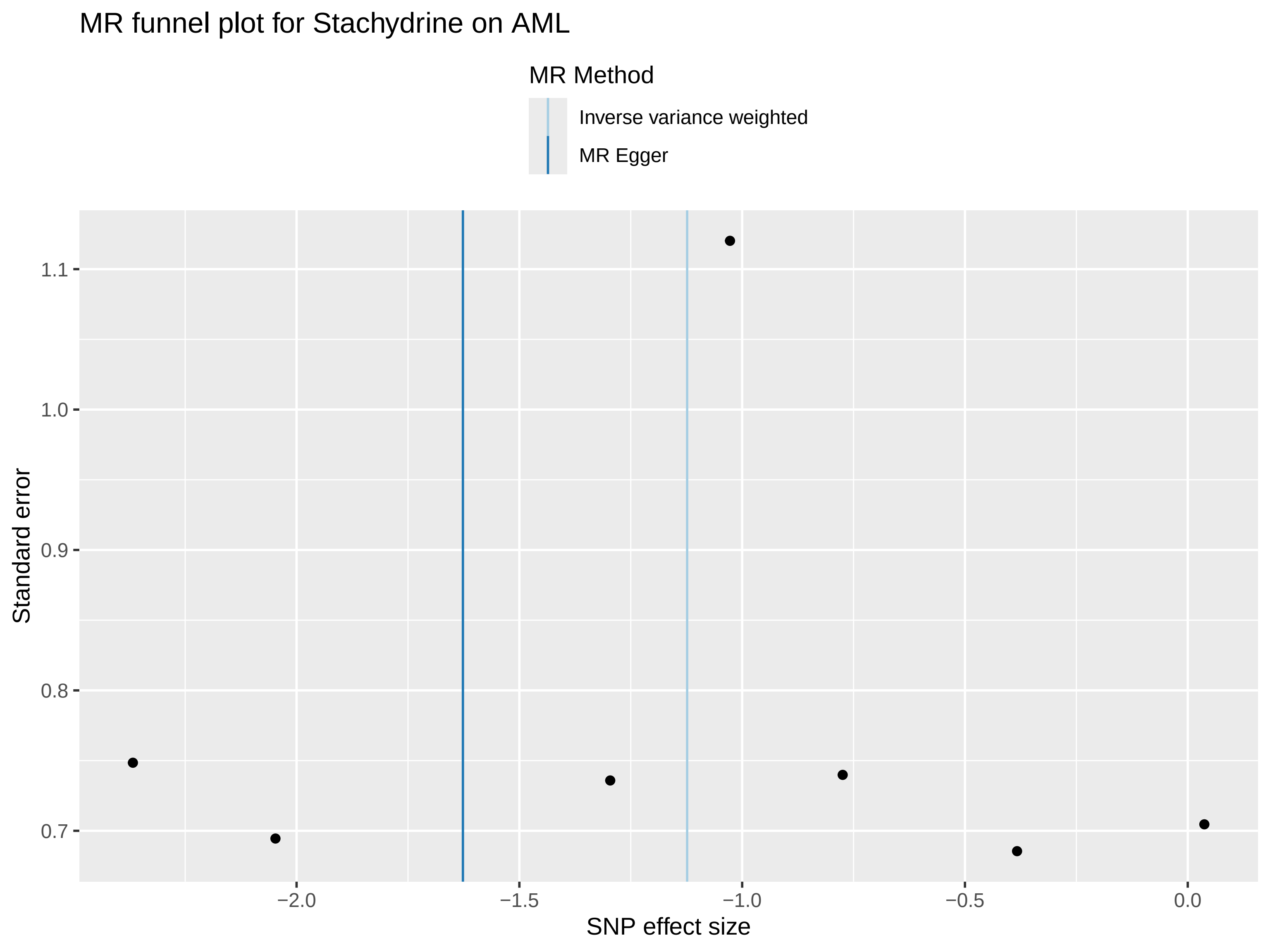
MR funnel plot for Stachydrine on AML
MR Method
Inverse variance weighted
MR Egger
Standard error
1.1
1.0
0.9
0.8
0.7
−2.0
−1.5
−1.0
−0.5
0.0
SNP effect size

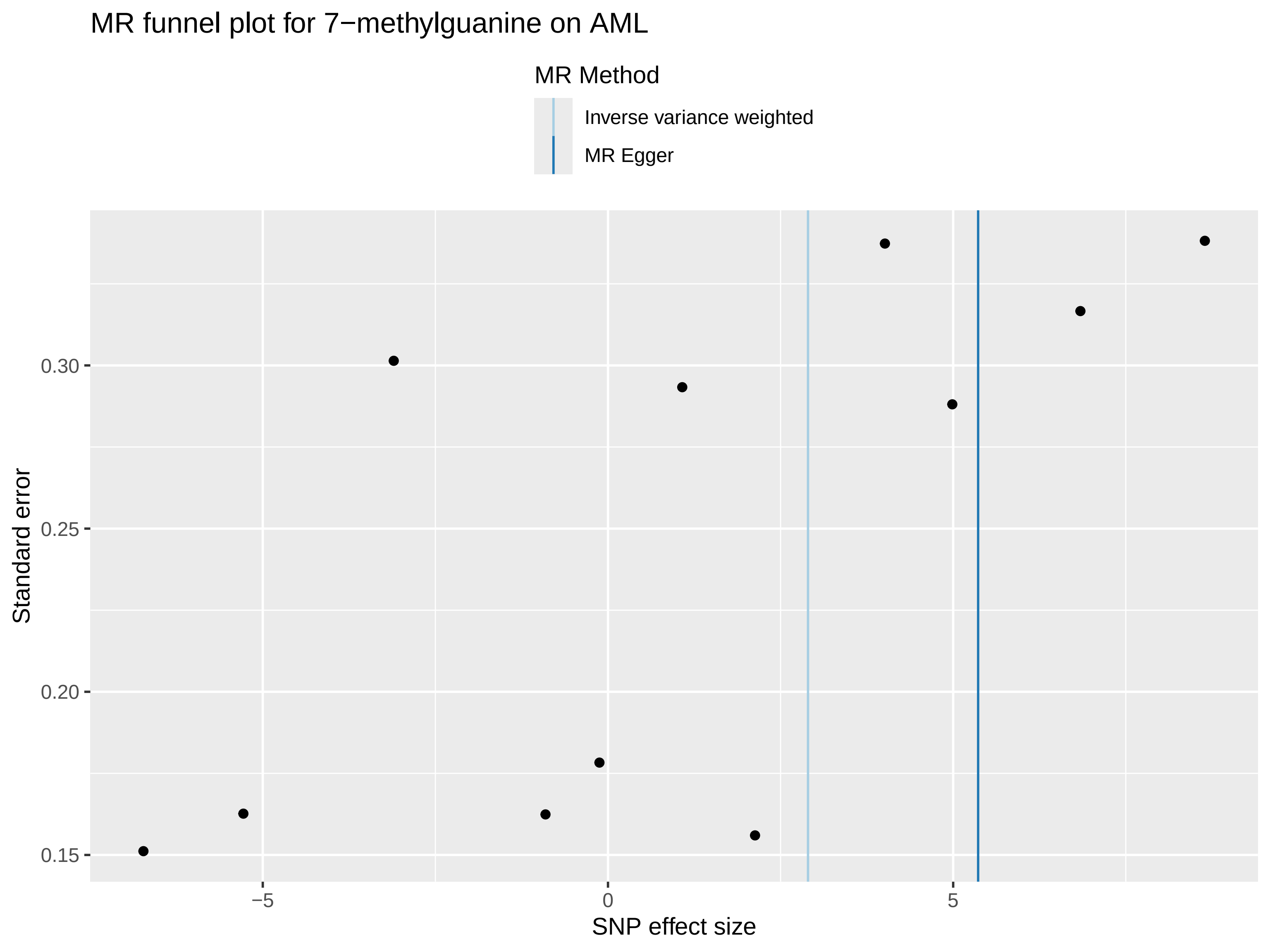
MR funnel plot for 7−methylguanine on AML
MR Method
Inverse variance weighted
MR Egger
Standard error
0.30
0.25
0.20
0.15
−5
0
5
SNP effect size

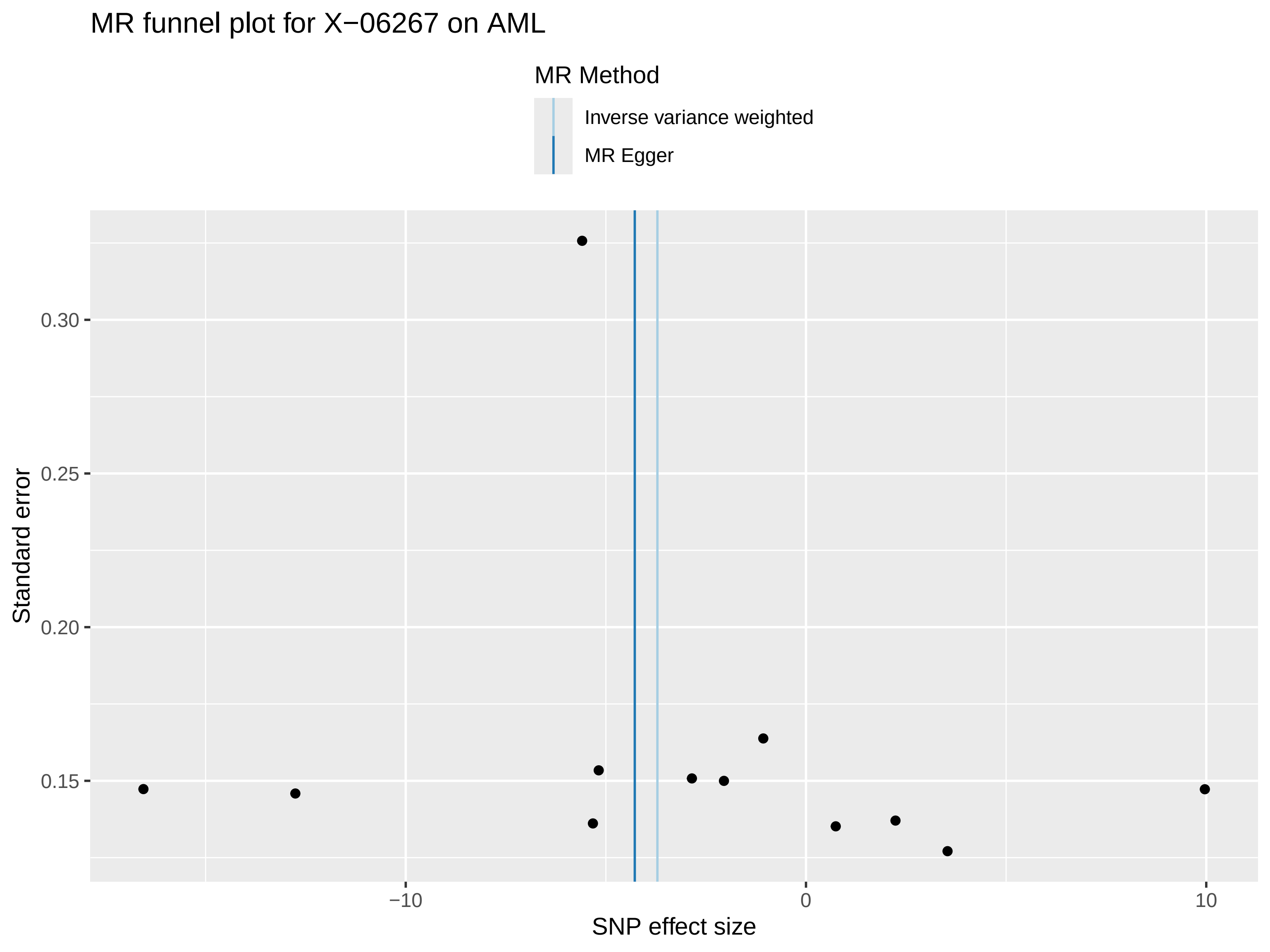
MR funnel plot for X−06267 on AML
MR Method
Inverse variance weighted
MR Egger
Standard error
0.30
0.25
0.20
0.15
SNP effect size
−10
0
10

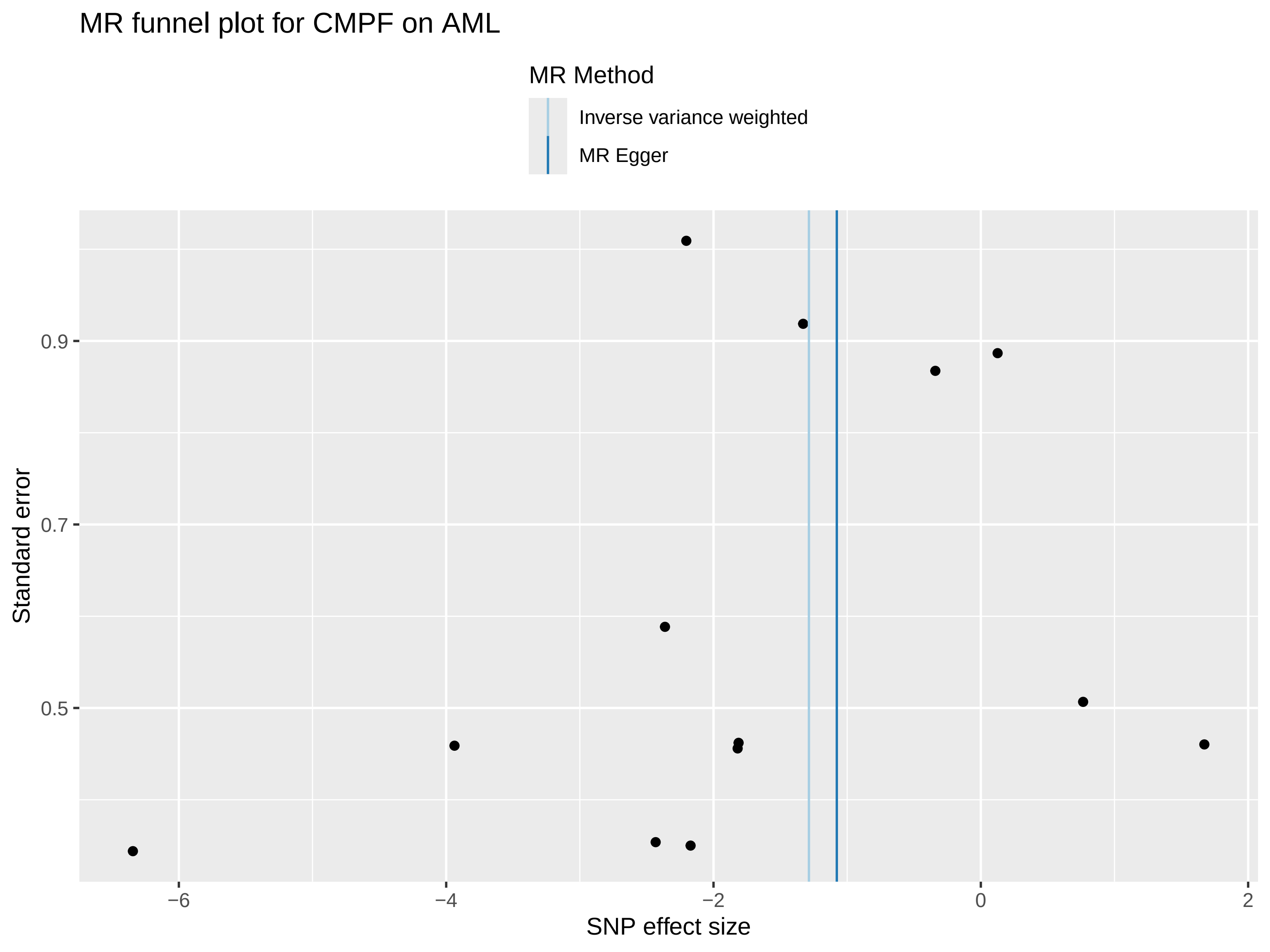
MR funnel plot for CMPF on AML
MR Method
Inverse variance weighted
MR Egger
Standard error
0.9
0.7
0.5
−6
−4
−2
0
2
SNP effect size

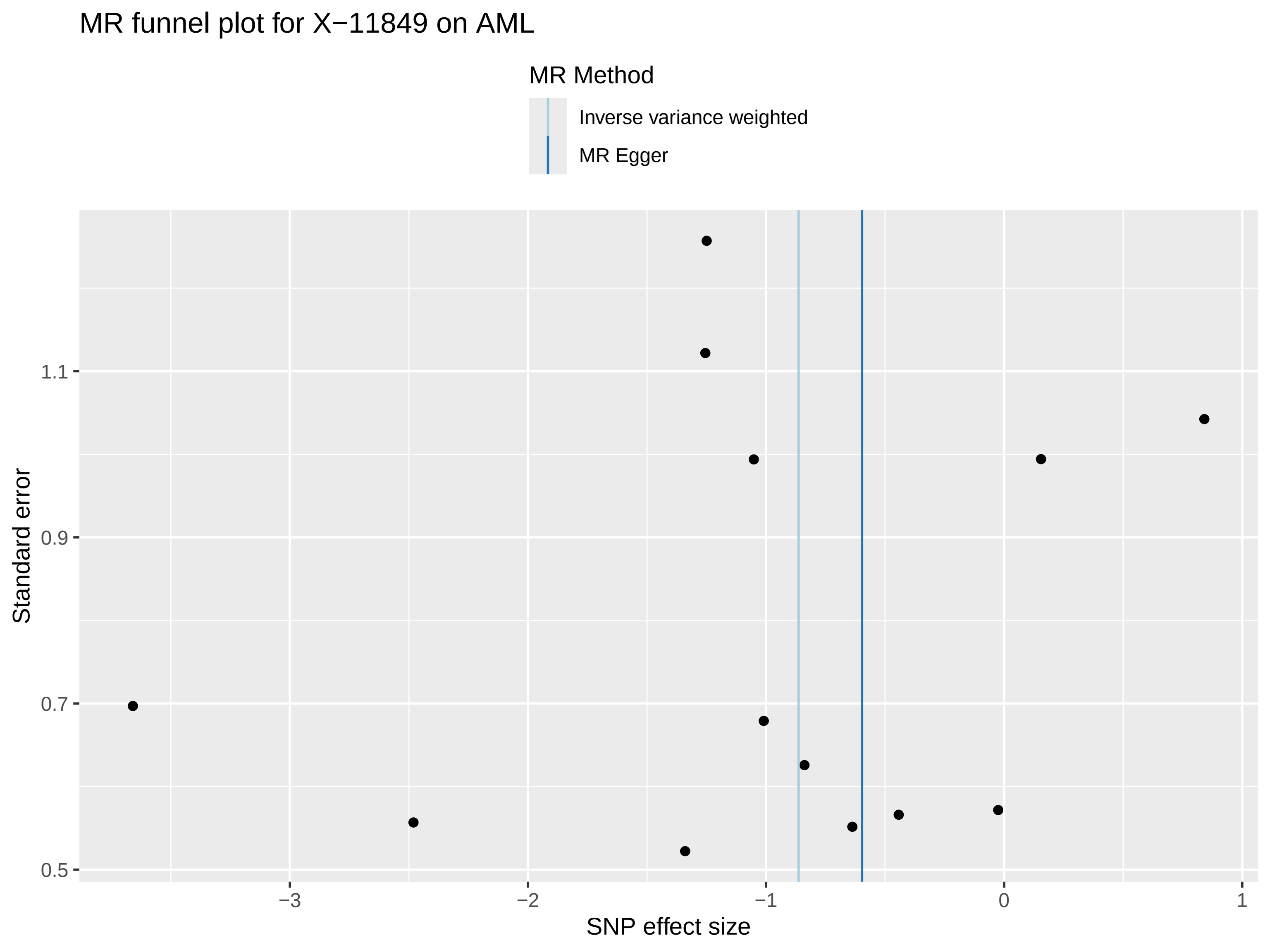
MR funnel plot for X−11849 on AML
MR Method
Inverse variance weighted
MR Egger
Standard error
1.1
0.9
0.7
0.5
−3
−2
−1
0
1
SNP effect size

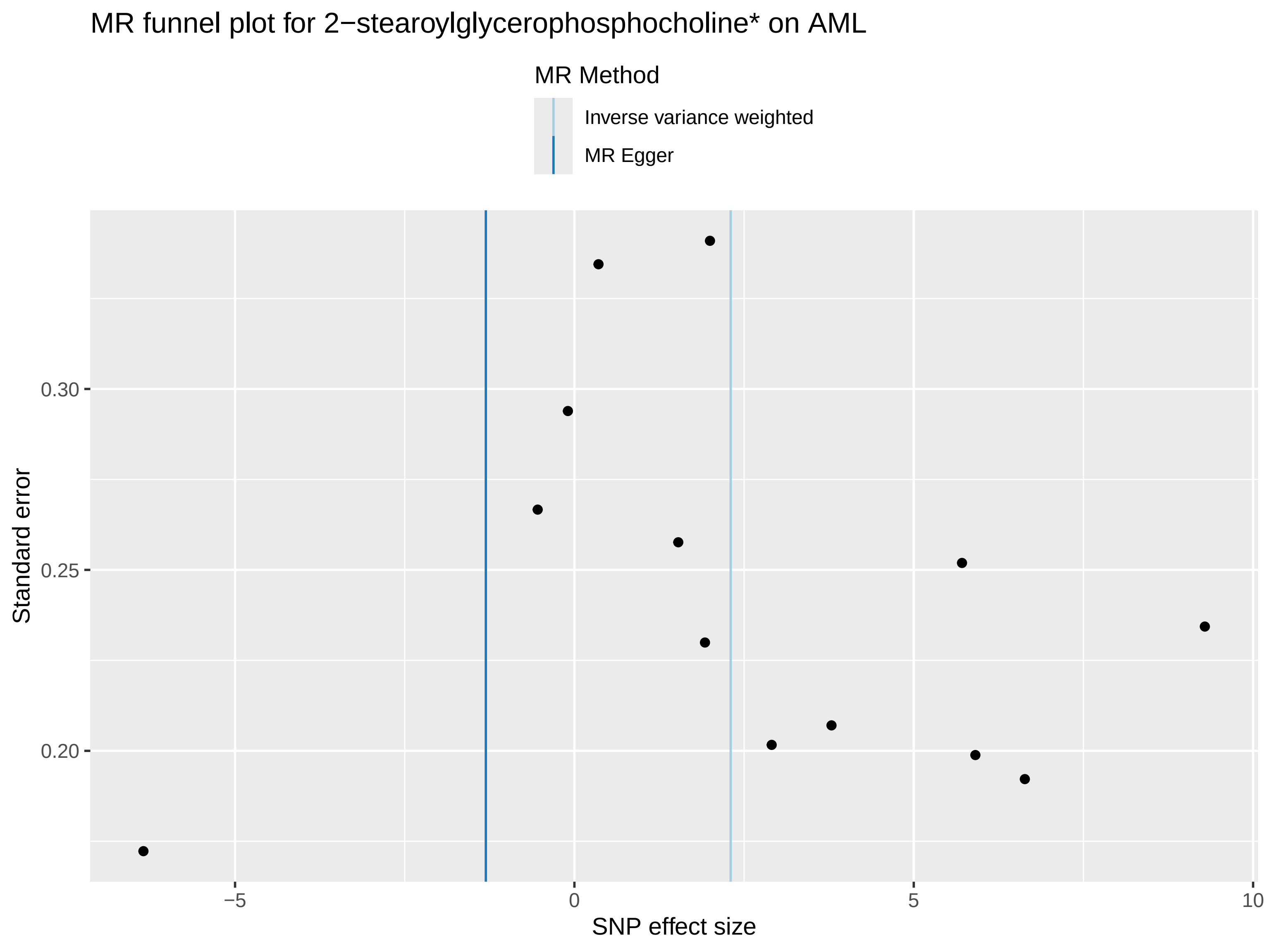
MR funnel plot for 2−stearoylglycerophosphocholine* on AML
MR Method
Inverse variance weighted
MR Egger
Standard error
0.30
0.25
0.20
SNP effect size
−5
0
5
10

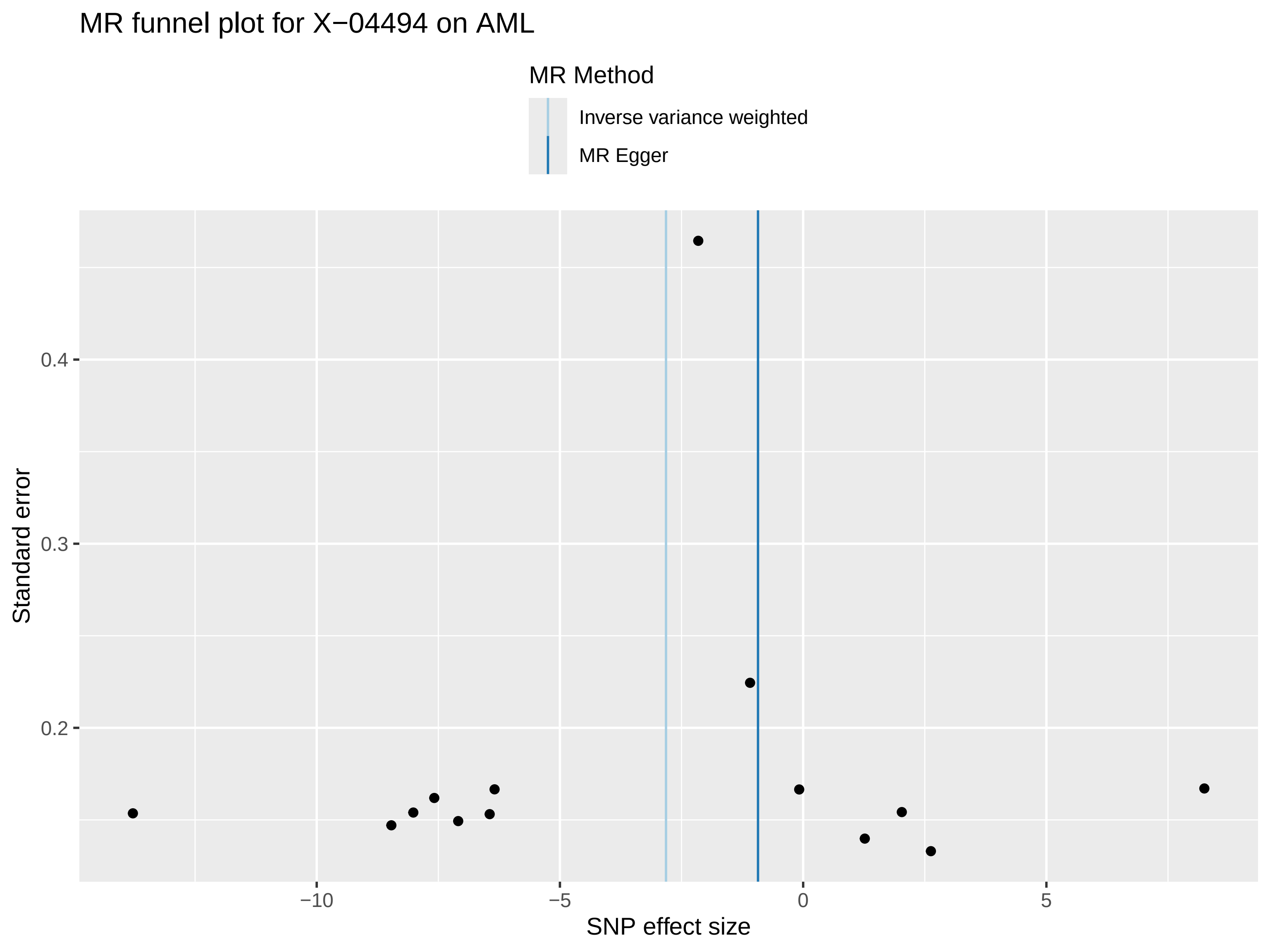
MR funnel plot for X−04494 on AML
MR Method
Inverse variance weighted
MR Egger
Standard error
0.4
0.3
0.2
−10
−5
0
5
SNP effect size

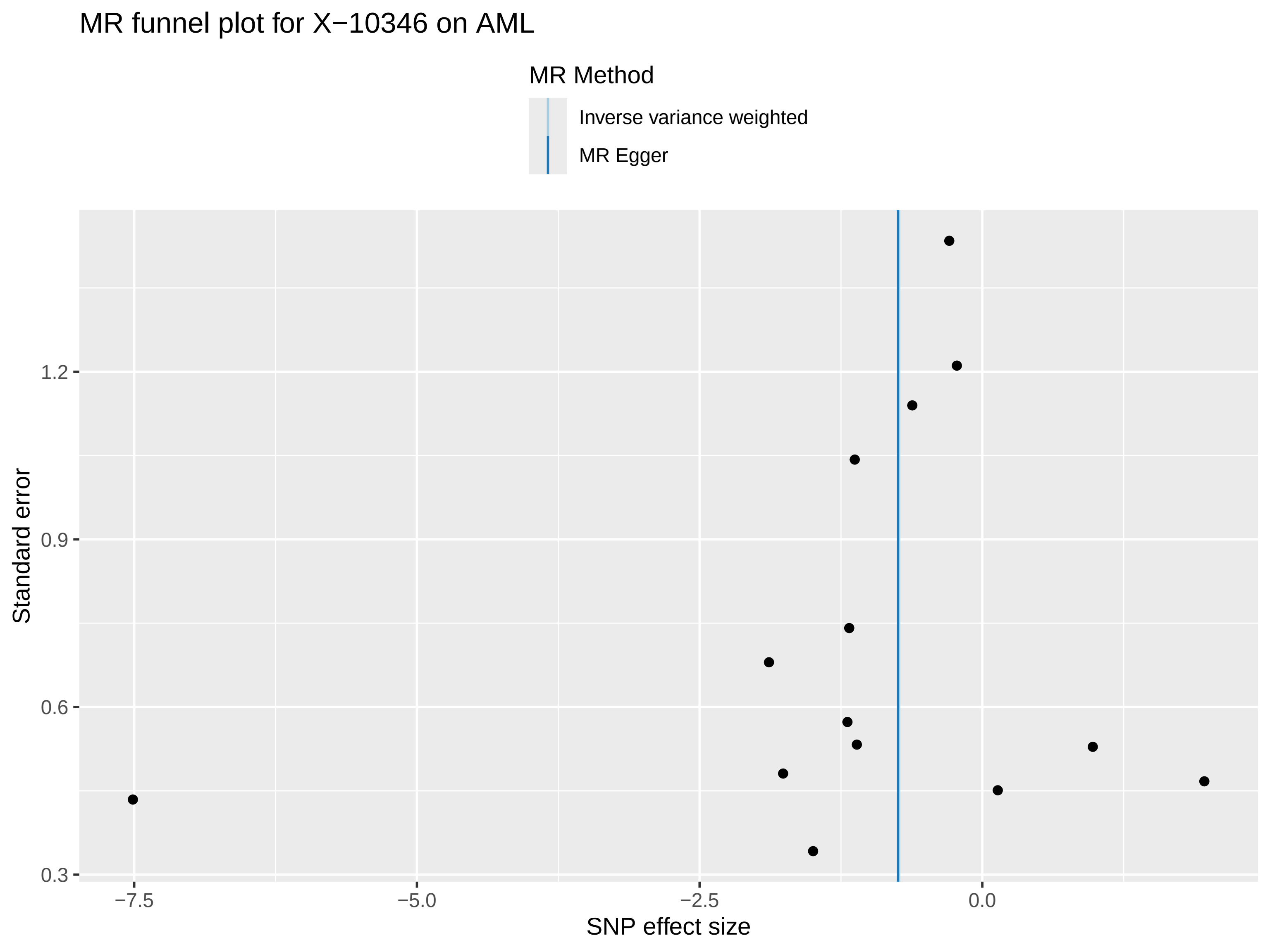
MR funnel plot for X−10346 on AML
MR Method
Inverse variance weighted
MR Egger
Standard error
1.2
0.9
0.6
0.3
−7.5
−5.0
−2.5
0.0
SNP effect size

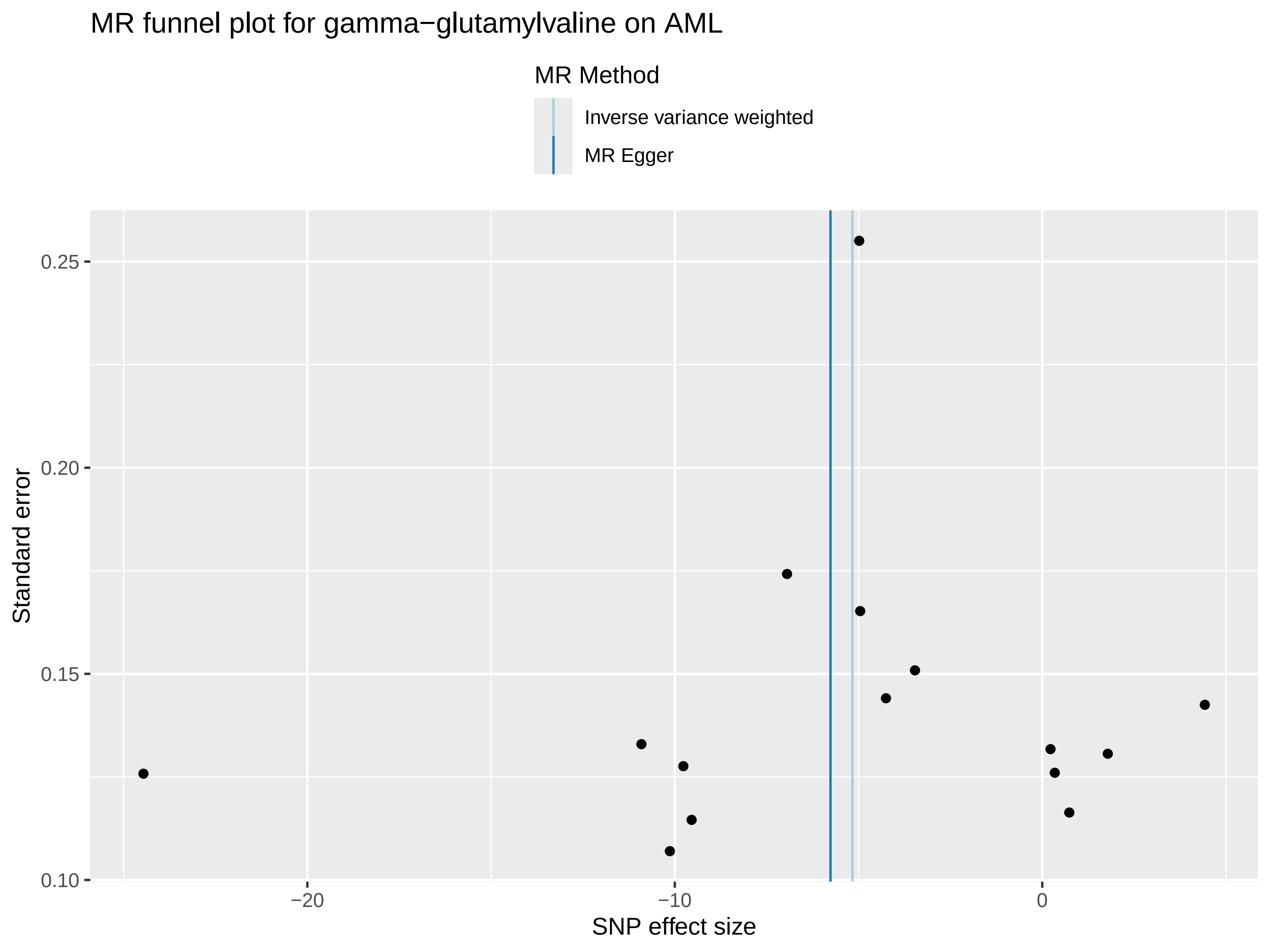
MR funnel plot for gamma−glutamylvaline on AML
MR Method
Inverse variance weighted
MR Egger
Standard error
0.25
0.20
0.15
0.10
−20
−10
0
SNP effect size

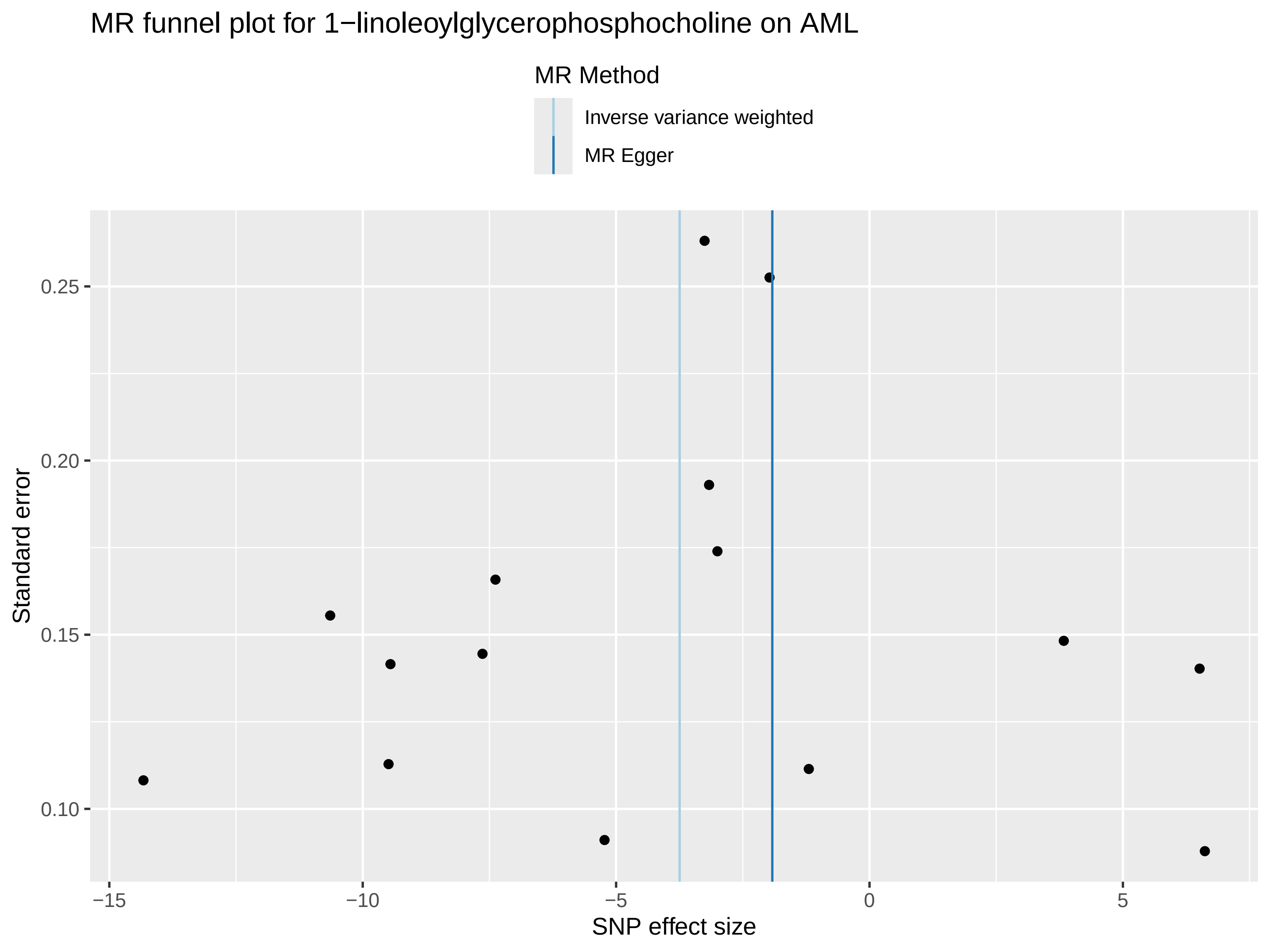

MR funnel plot for 1−linoleoylglycerophosphocholine on AML
MR Method
Inverse variance weighted
MR Egger
Standard error
0.25
0.20
0.15
0.10
−15
−10
−5
0
5
SNP effect size

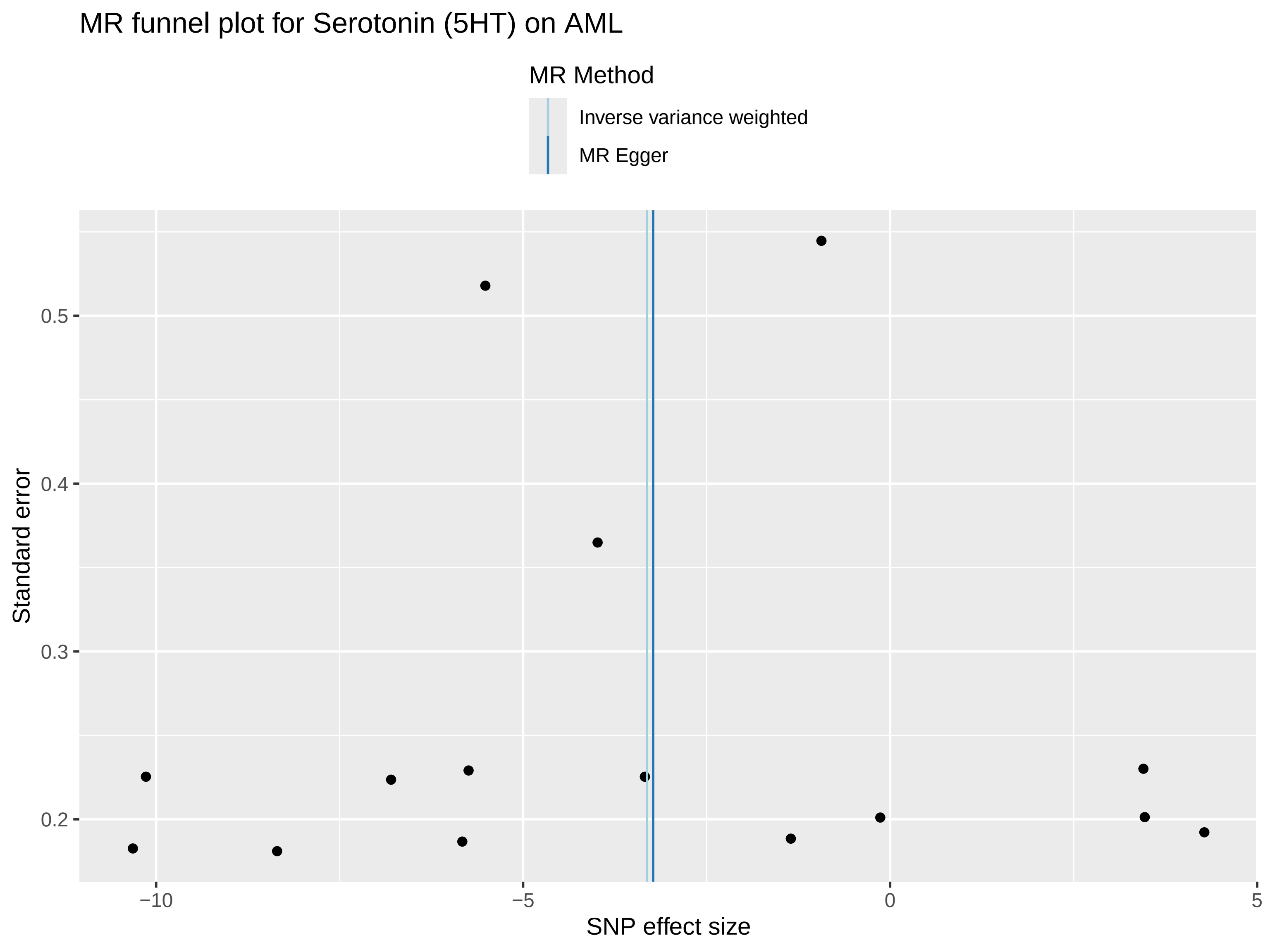
MR funnel plot for Serotonin (5HT) on AML
MR Method
Inverse variance weighted
MR Egger
Standard error
0.5
0.4
0.3
0.2
−10
−5
0
5
SNP effect size

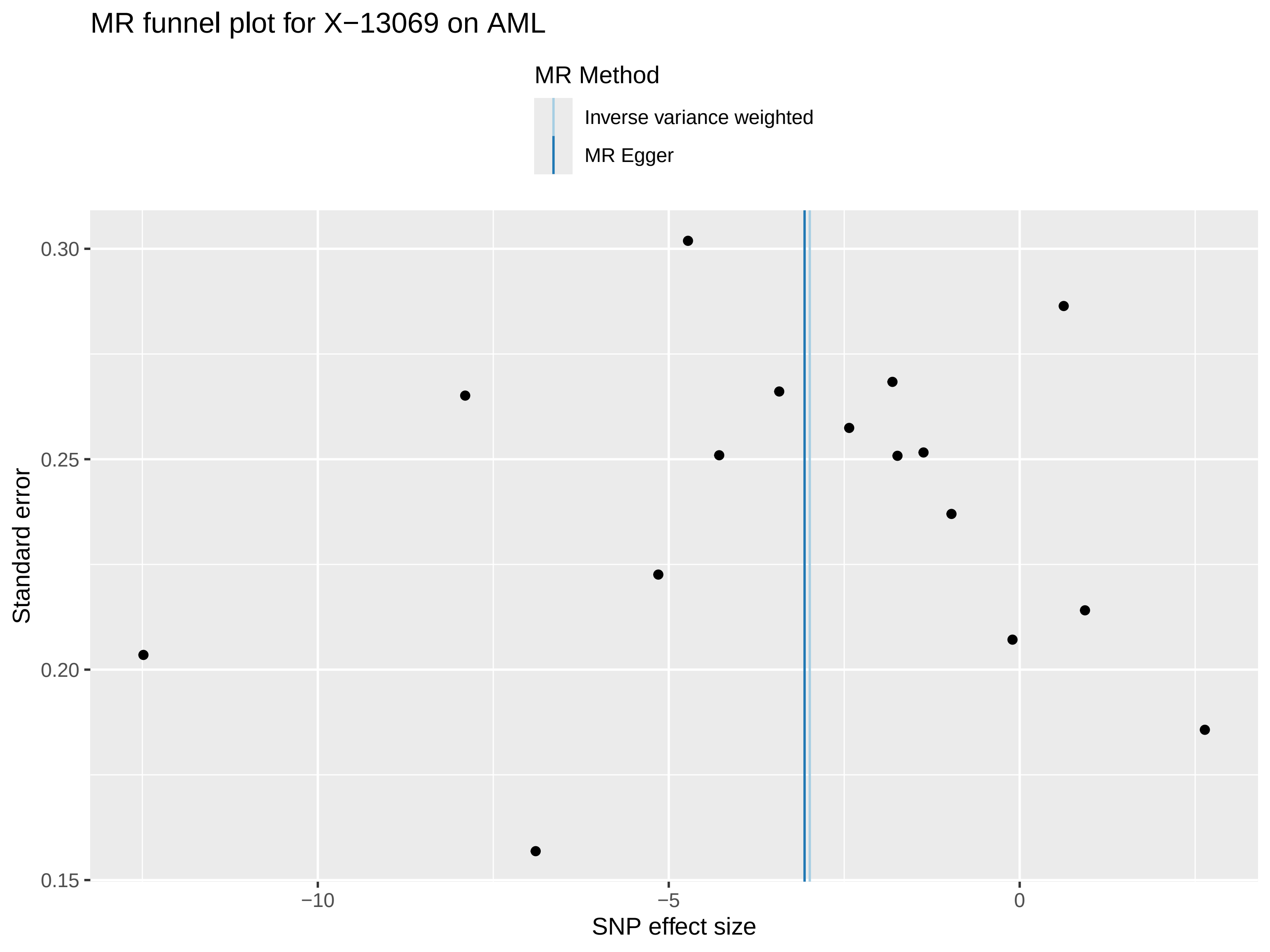
MR funnel plot for X−13069 on AML
MR Method
Inverse variance weighted
MR Egger
Standard error
0.30
0.25
0.20
0.15
−10
−5
0
SNP effect size

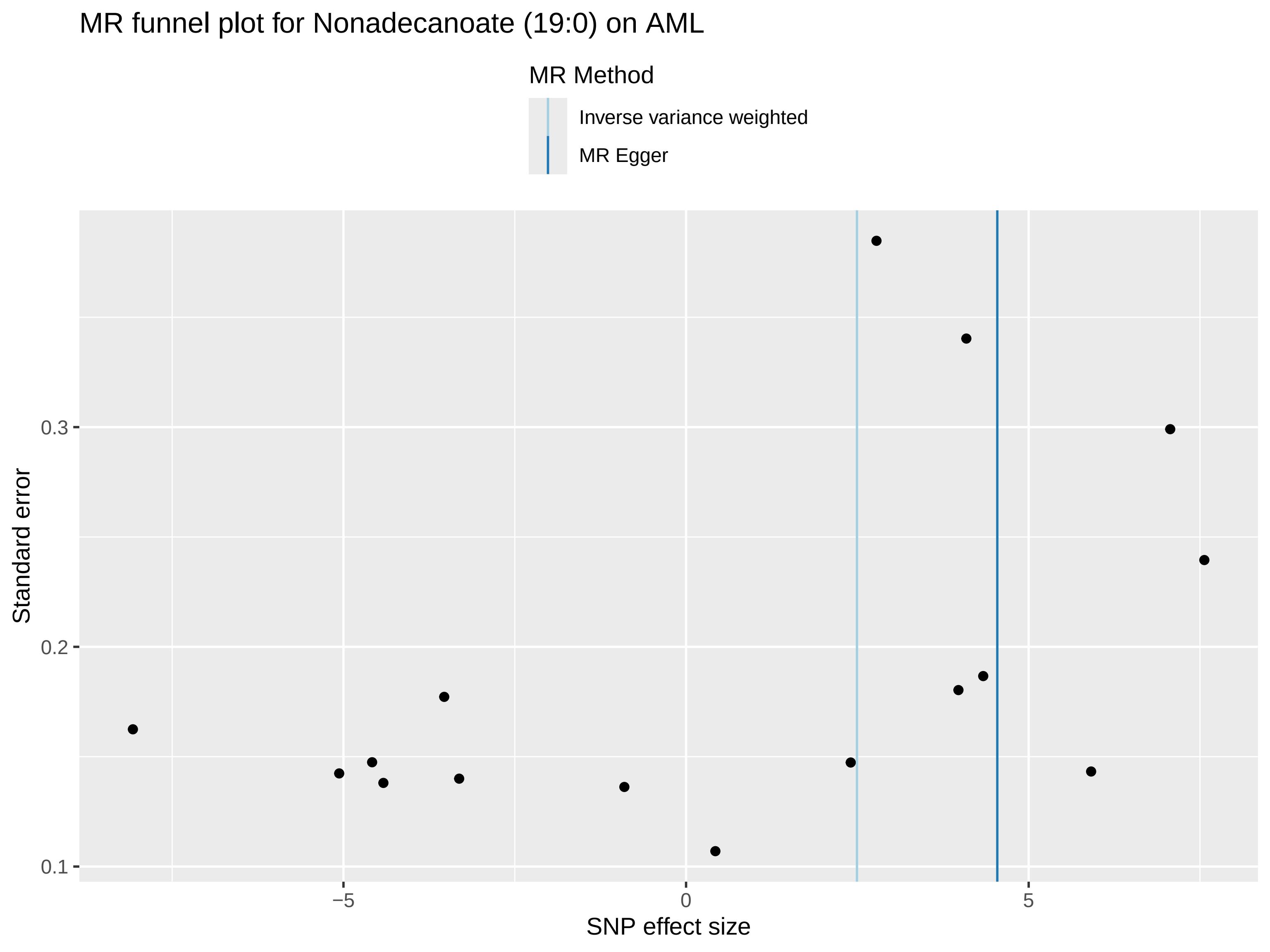
MR funnel plot for Nonadecanoate (19:0) on AML
MR Method
Inverse variance weighted
MR Egger
Standard error
0.3
0.2
0.1
−5
0
5
SNP effect size

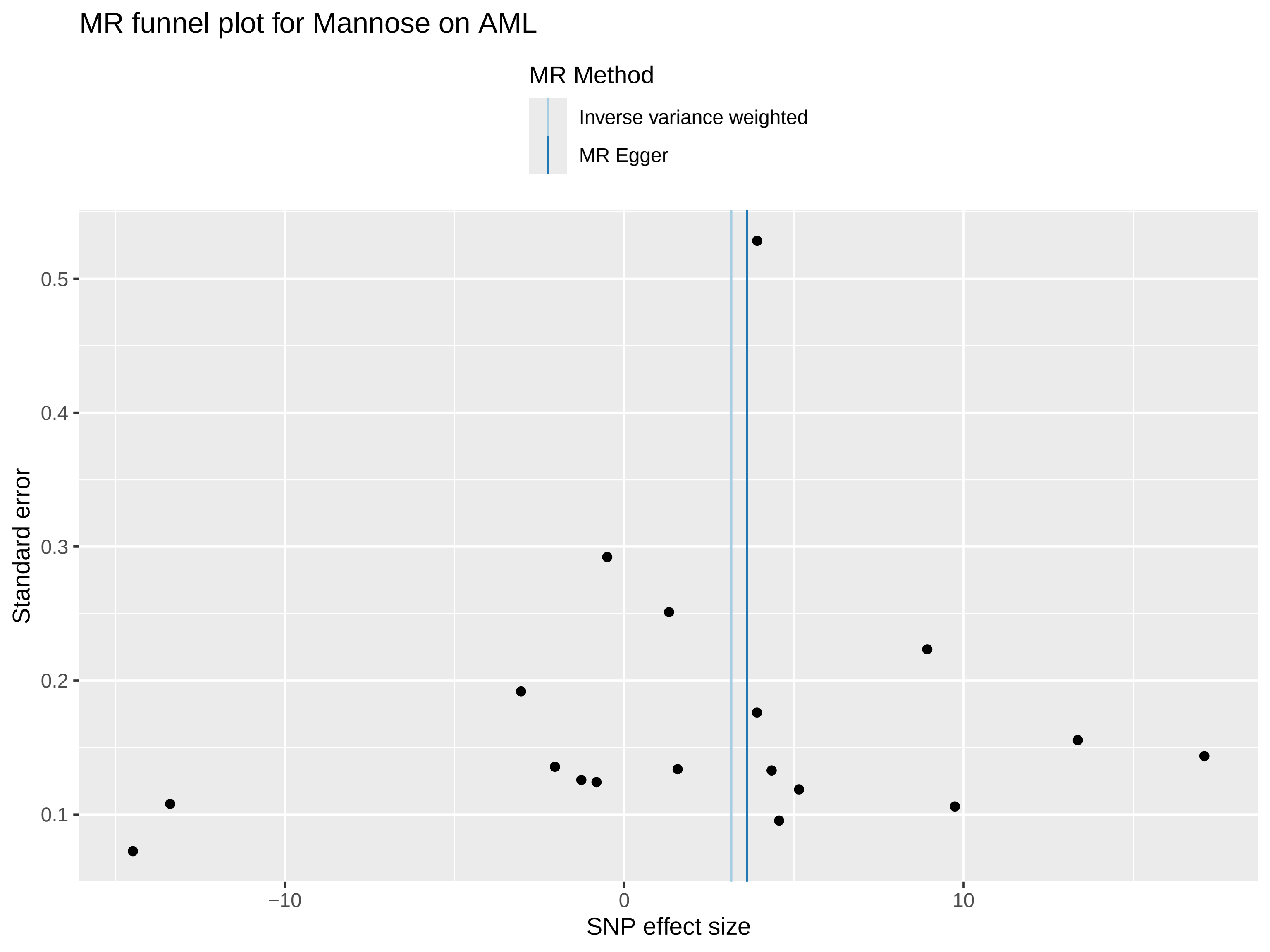
MR funnel plot for Mannose on AML
MR Method
Inverse variance weighted
MR Egger
Standard error
0.5
0.4
0.3
0.2
0.1
SNP effect size
−10
0
10

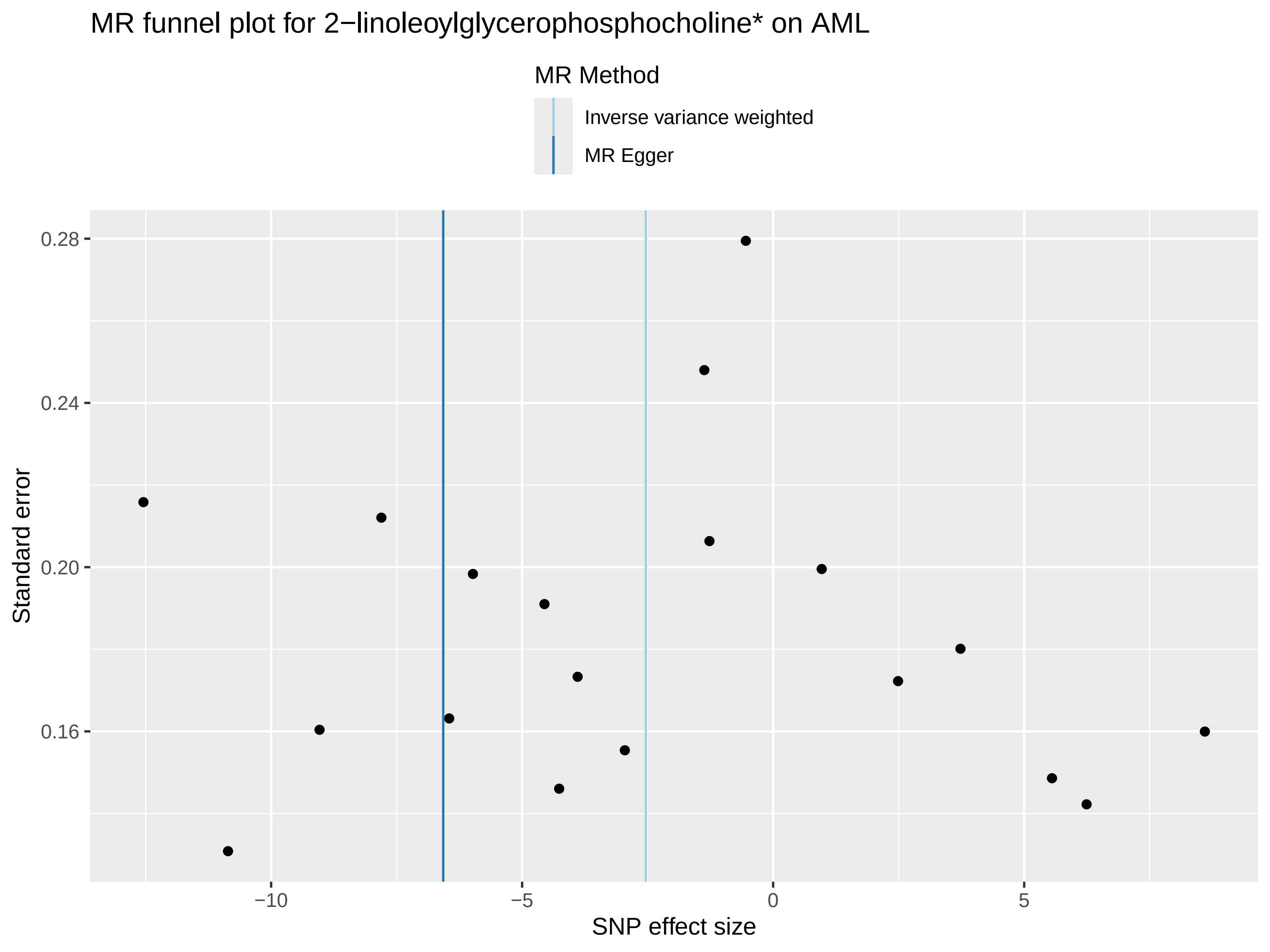

MR funnel plot for 2−linoleoylglycerophosphocholine* on AML
MR Method
Inverse variance weighted
MR Egger
Standard error
0.28
0.24
0.20
0.16
−10
−5
0
5
SNP effect size

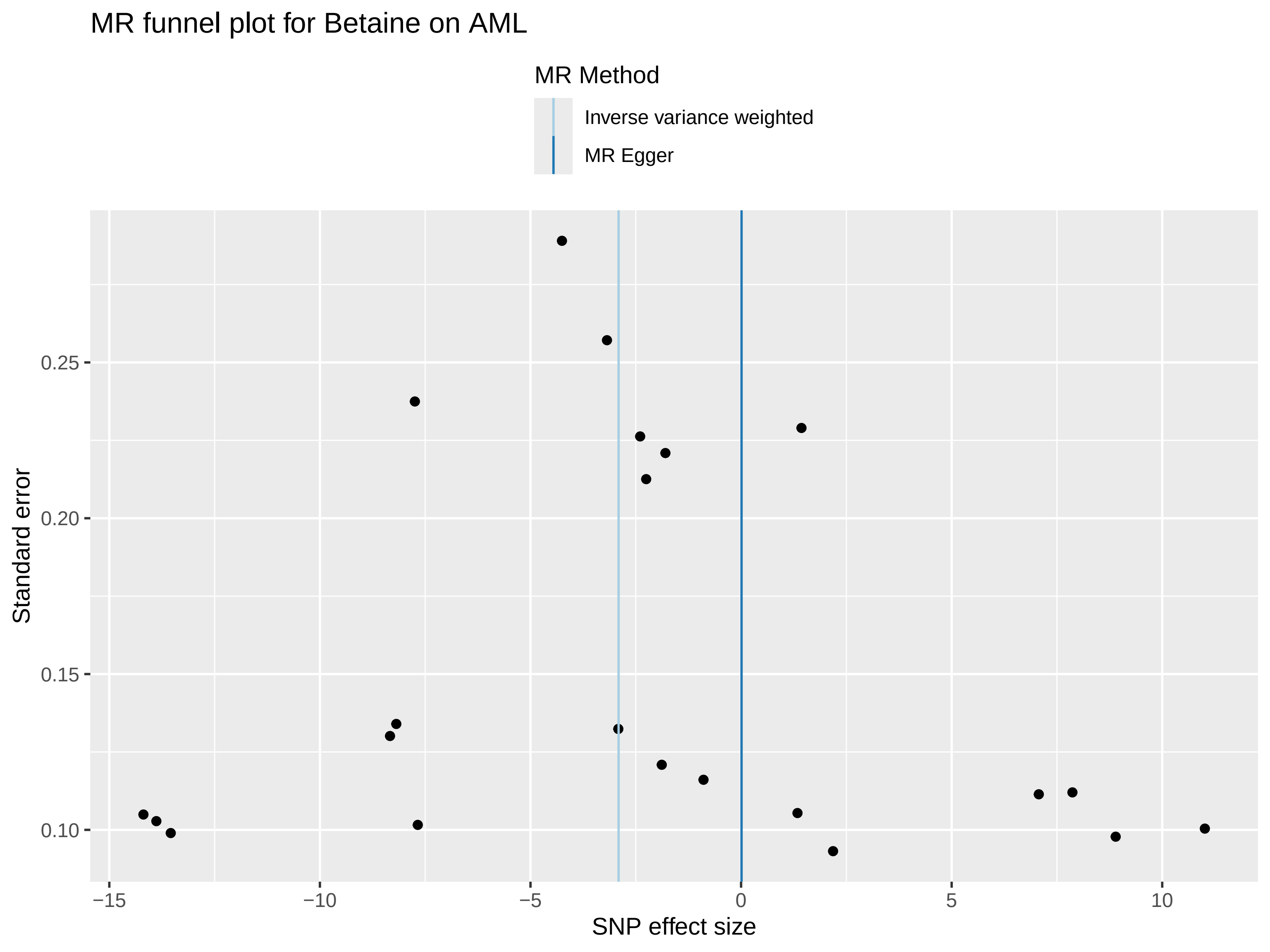
MR funnel plot for Betaine on AML
MR Method
Inverse variance weighted
MR Egger
Standard error
0.25
0.20
0.15
0.10
−15
−10
−5
0
5
10
SNP effect size

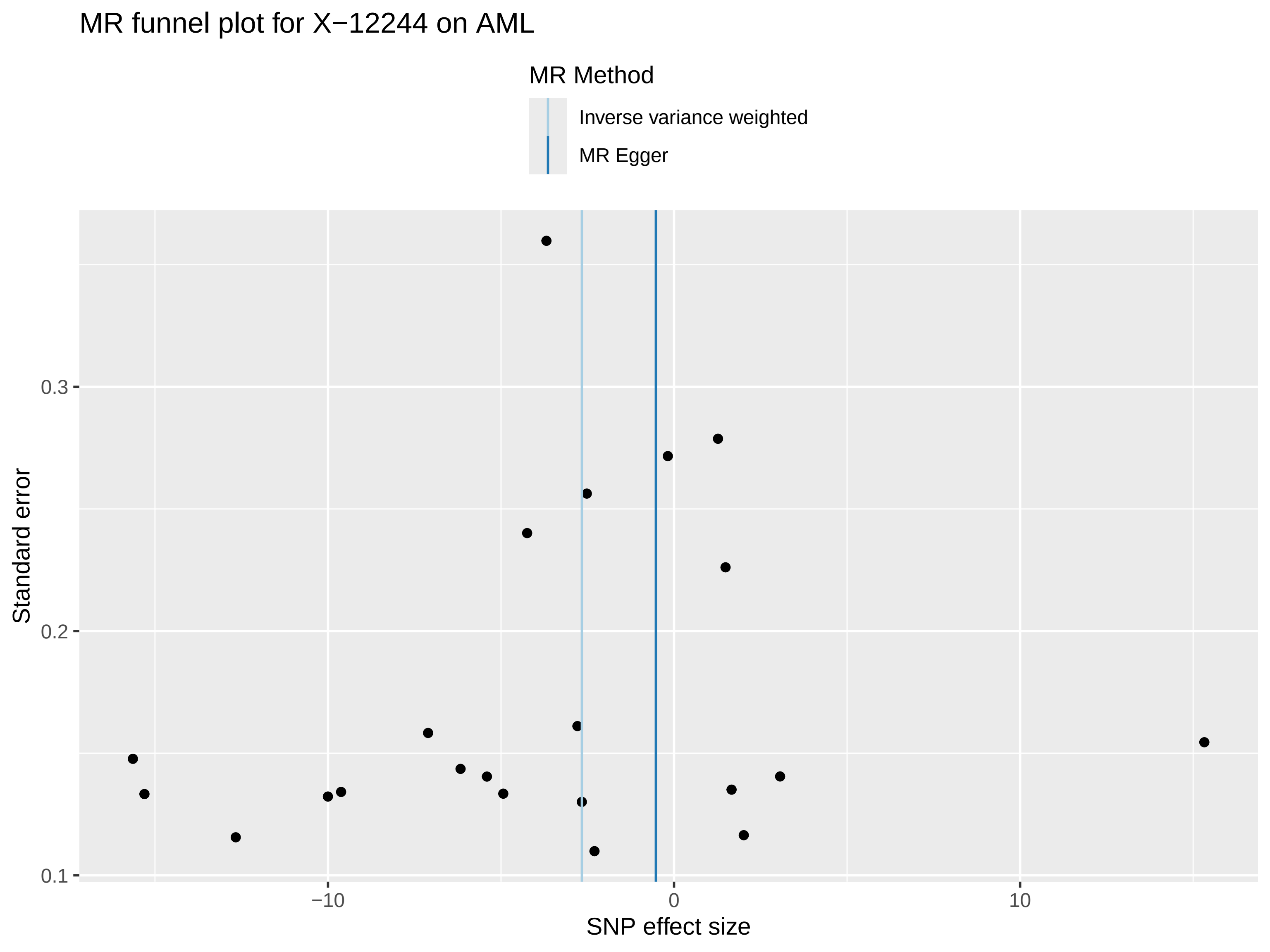
MR funnel plot for X−12244 on AML
MR Method
Inverse variance weighted
MR Egger
Standard error
0.3
0.2
0.1
−10
0
10
SNP effect size

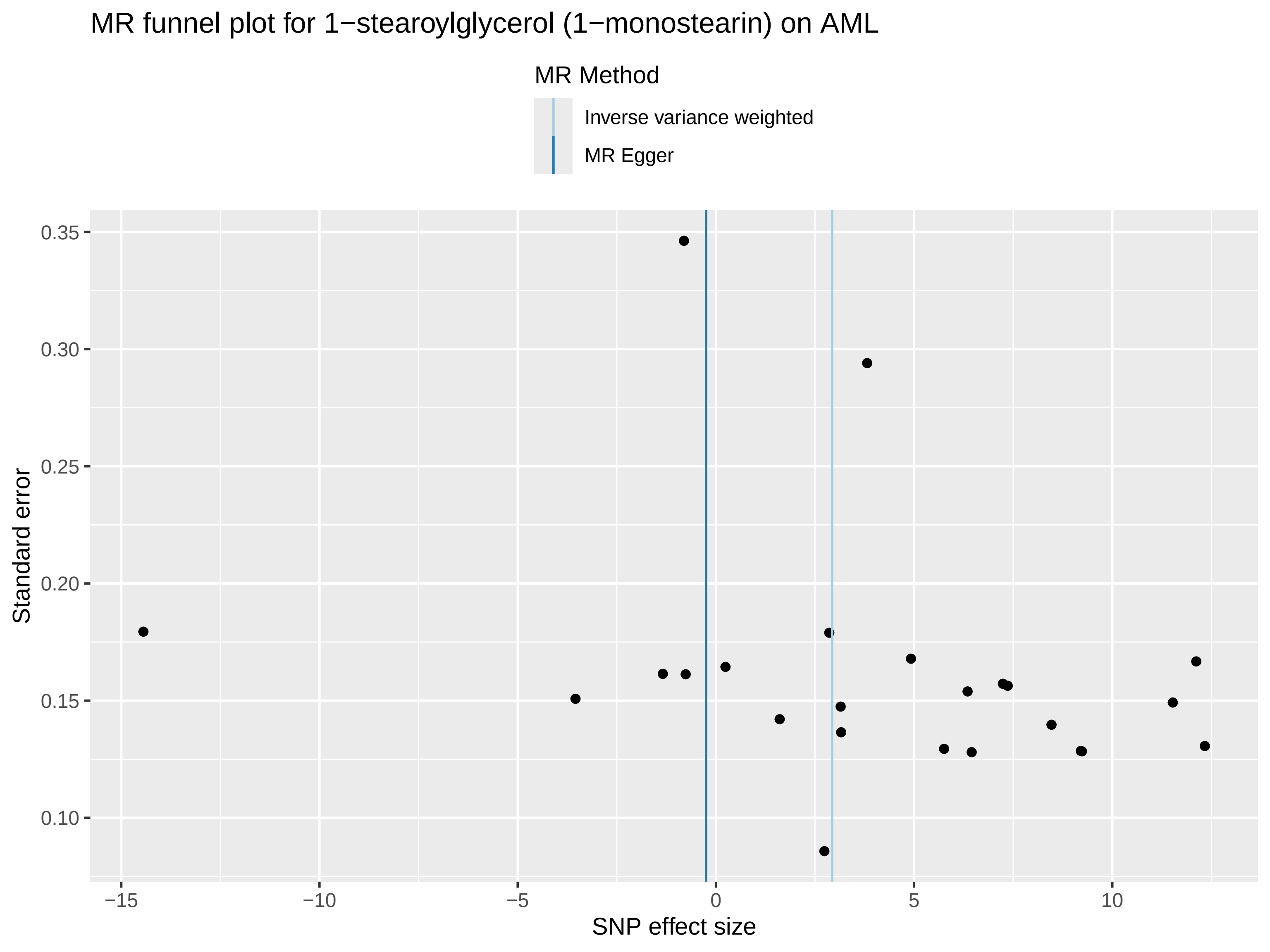
MR funnel plot for 1−stearoylglycerol (1−monostearin) on AML
MR Method
Inverse variance weighted
MR Egger
Standard error
0.35
0.30
0.25
0.20
0.15
0.10
−15
−10
−5
0
5
10
SNP effect size

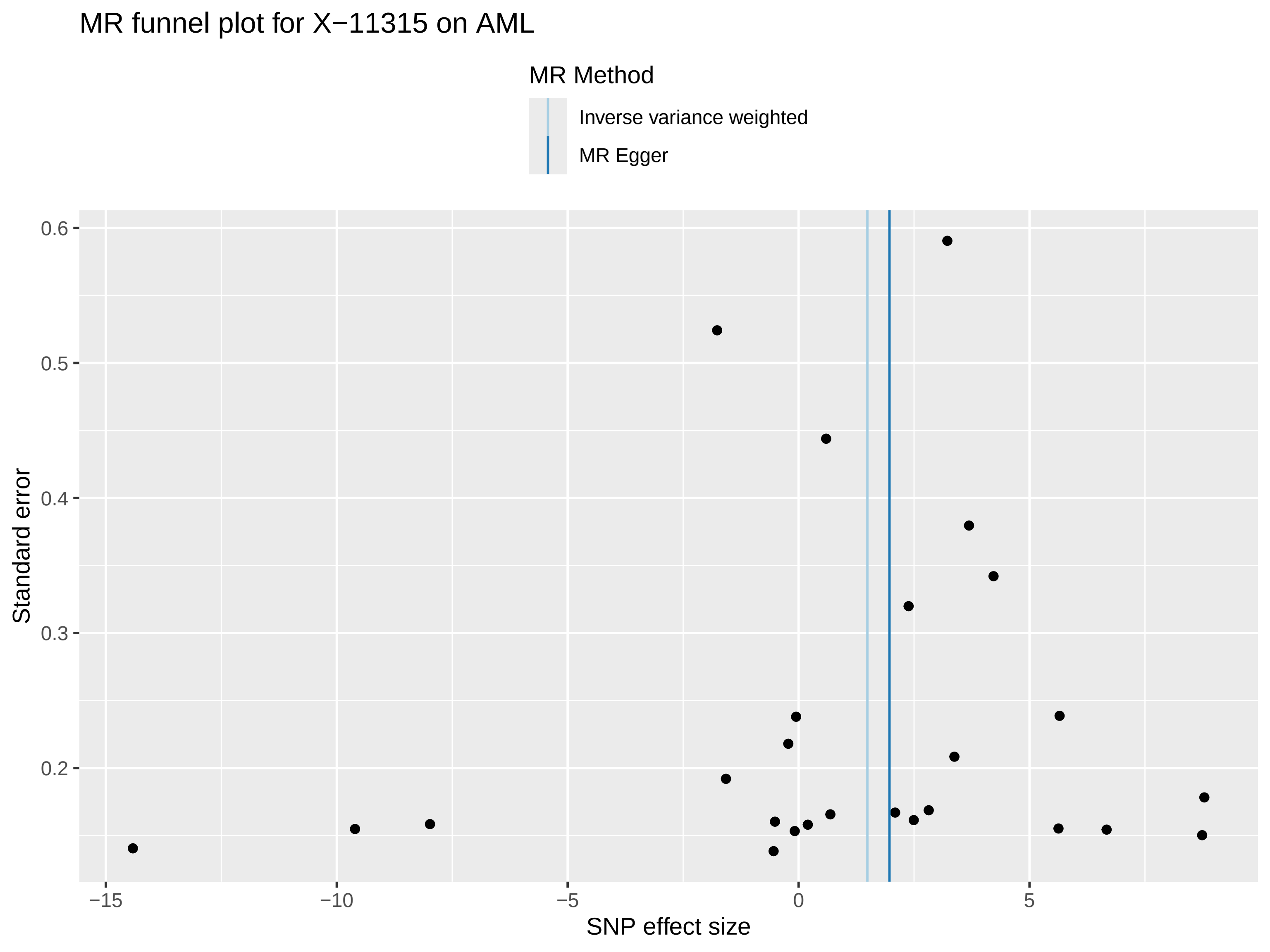
MR funnel plot for X−11315 on AML
MR Method
Inverse variance weighted
MR Egger
Standard error
SNP effect size
0.6
0.5
0.4
0.3
0.2
−15
−10
−5
0
5

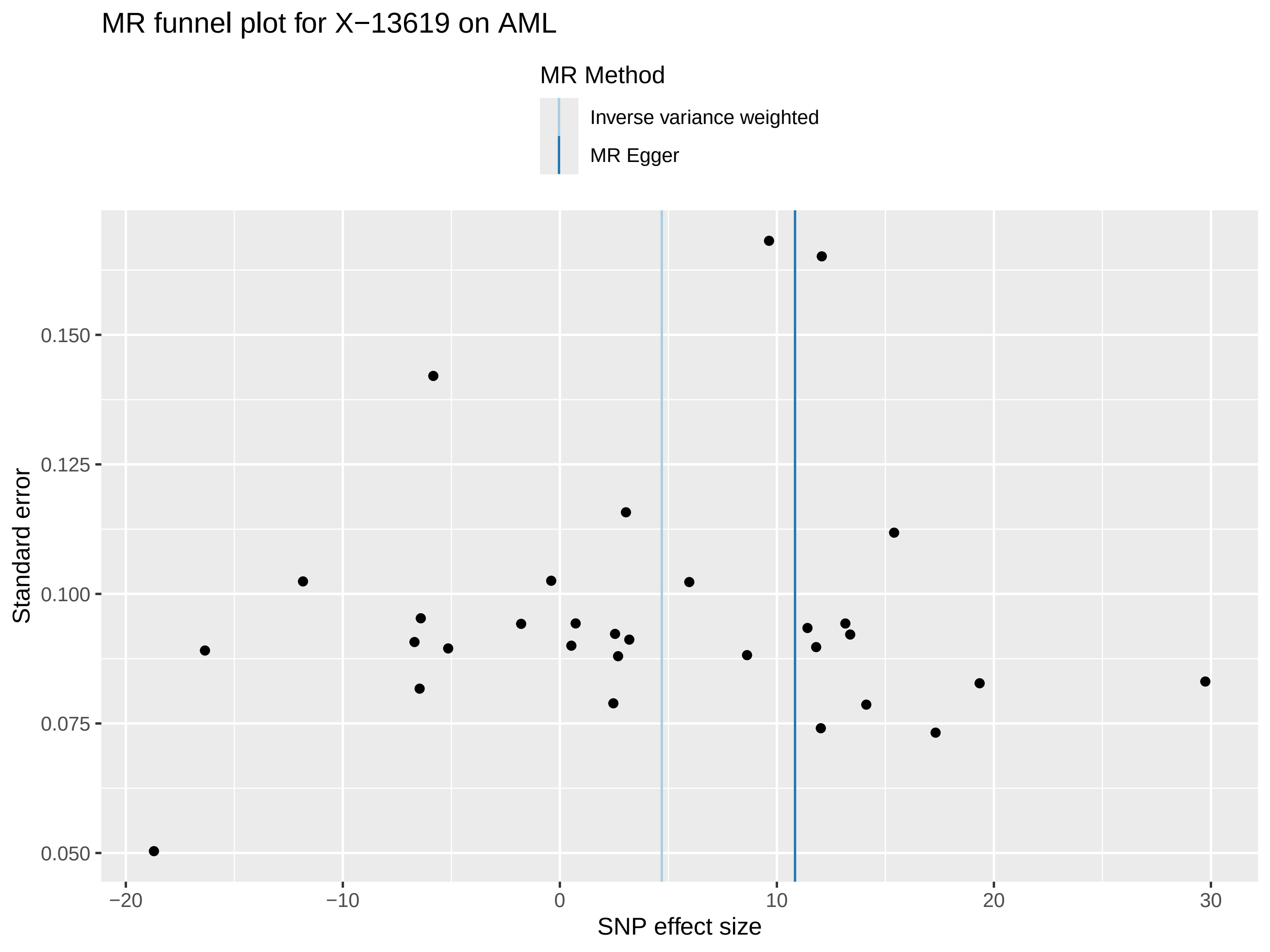
MR funnel plot for X−13619 on AML
MR Method
Inverse variance weighted
MR Egger
Standard error
0.150
0.125
0.100
0.075
0.050
−20
−10
0
10
20
30
SNP effect size

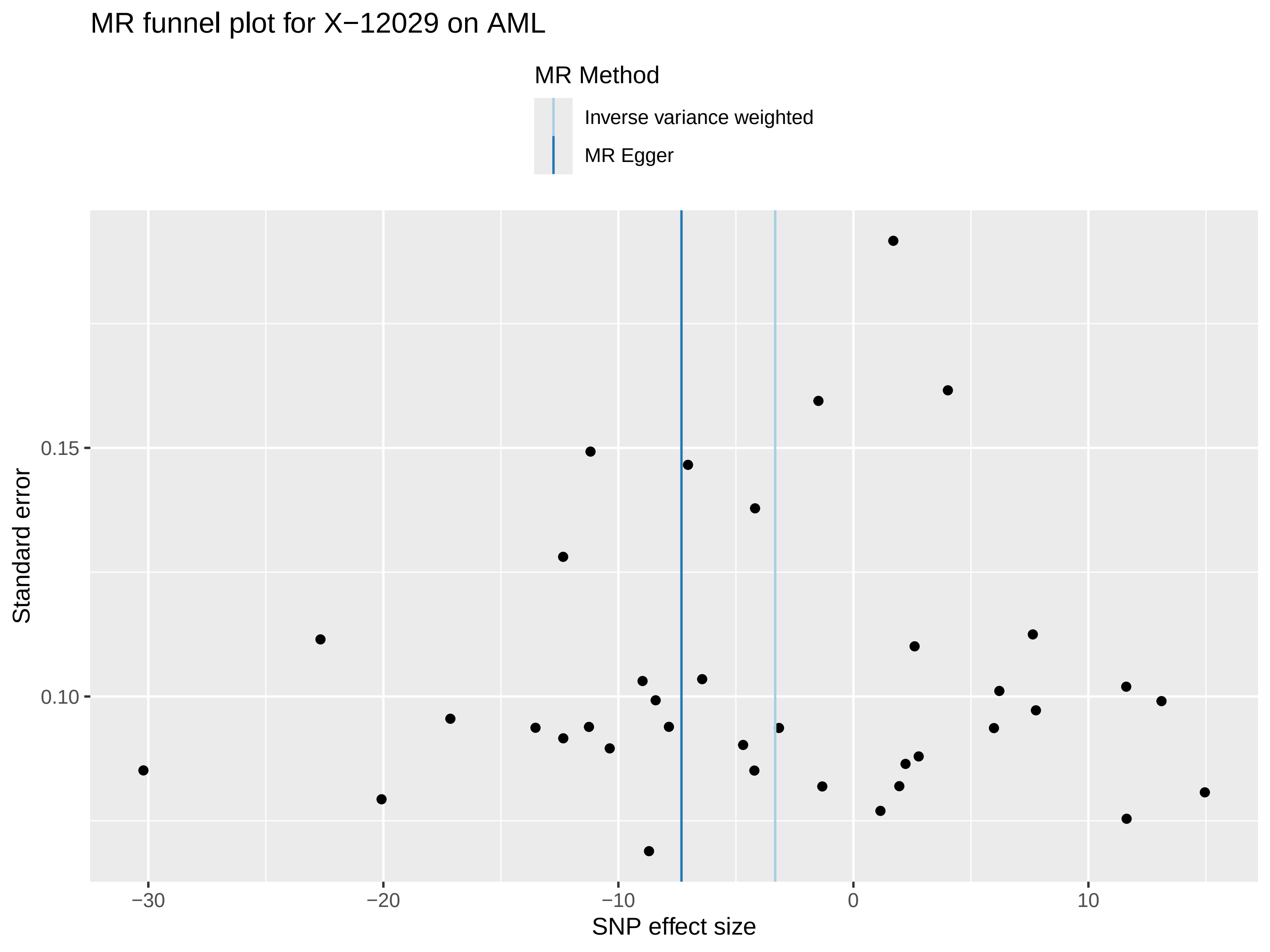

MR funnel plot for X−12029 on AML
MR Method
Inverse variance weighted
MR Egger
Standard error
0.15
0.10
SNP effect size
−30
−20
−10
0
10

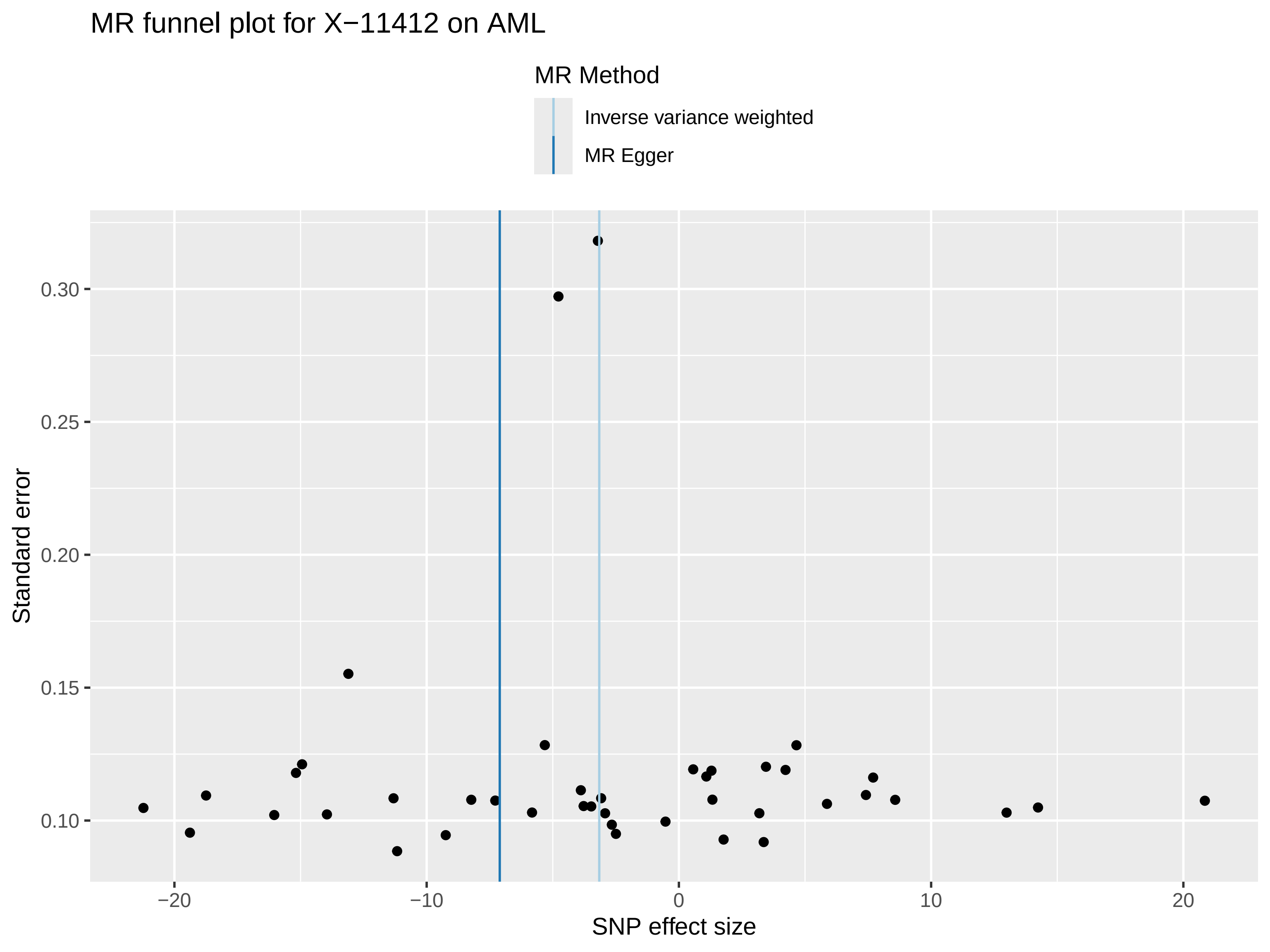
MR funnel plot for X−11412 on AML
MR Method
Inverse variance weighted
MR Egger
Standard error
0.30
0.25
0.20
0.15
0.10
SNP effect size
−20
−10
0
10
20
