# Supplementary material for: Bidirectional Mendelian Randomization and Multi-Omics Uncover Causal Serum Metabolites and Neuro-Related Mechanistic Pathways in Acute Myeloid Leukemia
Source: Int J Mol Sci. 2025 Nov 22;26(23):11307. doi: 10.3390/ijms262311307 (PMC12692008; doi:10.3390/ijms262311307)
Supplement: Supplementary file 1 [file ijms-26-11307-s001.zip › Figure S3. Leave-one-out plots for the 23 potential metabolites on AML..pdf]

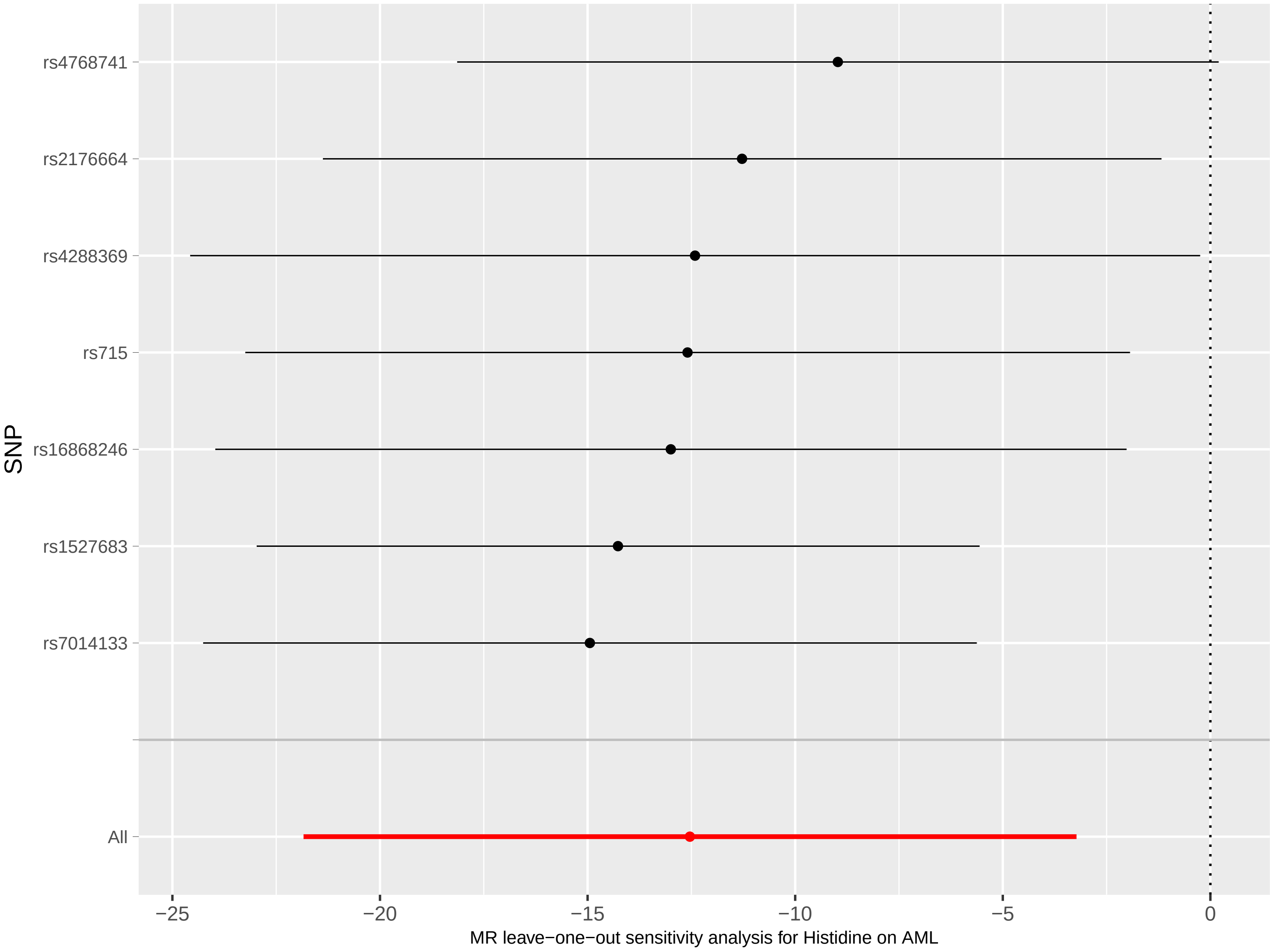
rs4768741
rs2176664
rs4288369
rs715
rs16868246
rs1527683
rs7014133
All
SNP
−25
−20
−15
−10
−5
0
MR leave−one−out sensitivity analysis for Histidine on AML

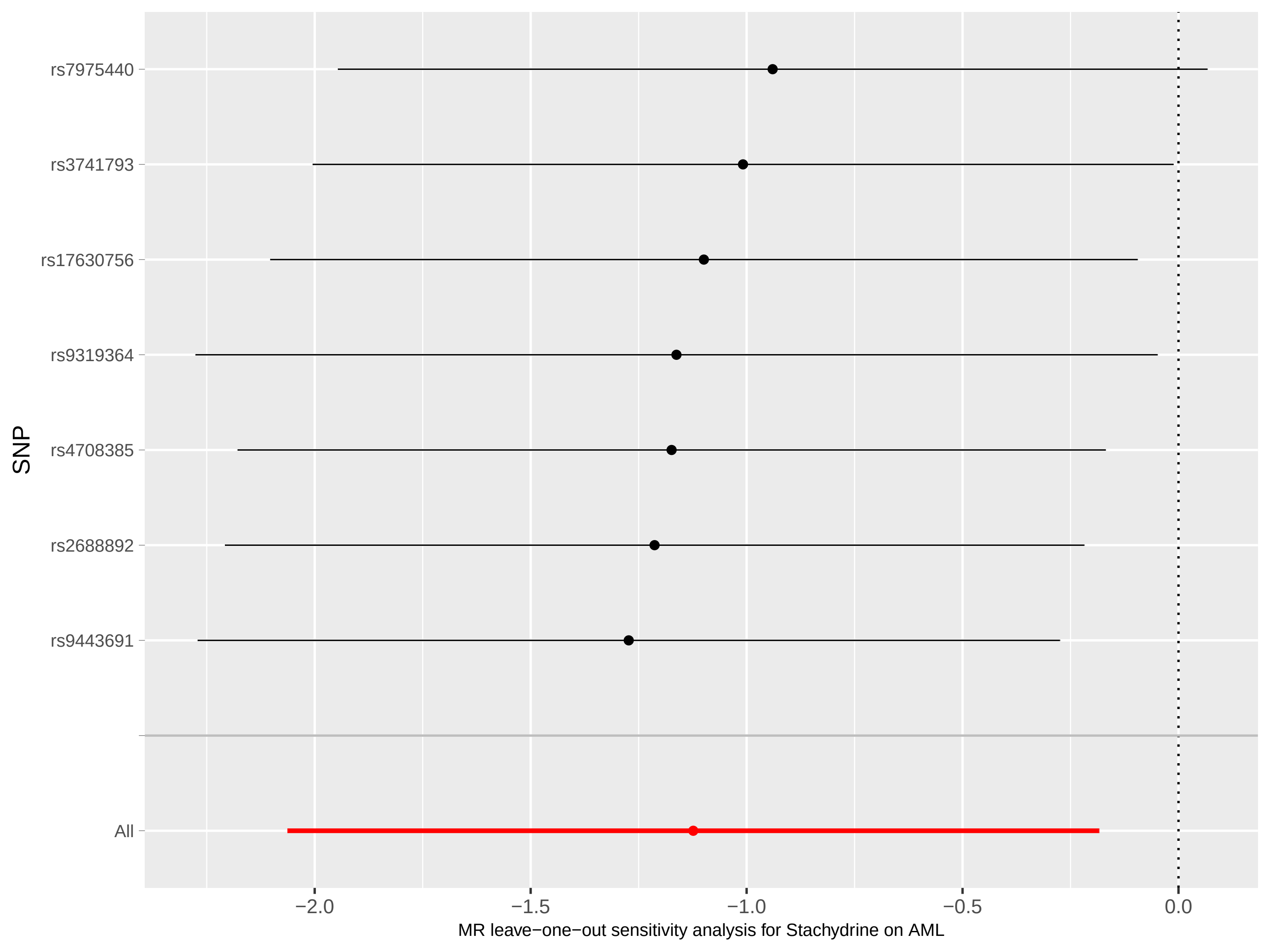

SNP
rs7975440
rs3741793
rs17630756
rs9319364
rs4708385
rs2688892
rs9443691
All
−2.0
−1.5
−1.0
−0.5
0.0
MR leave−one−out sensitivity analysis for Stachydrine on AML

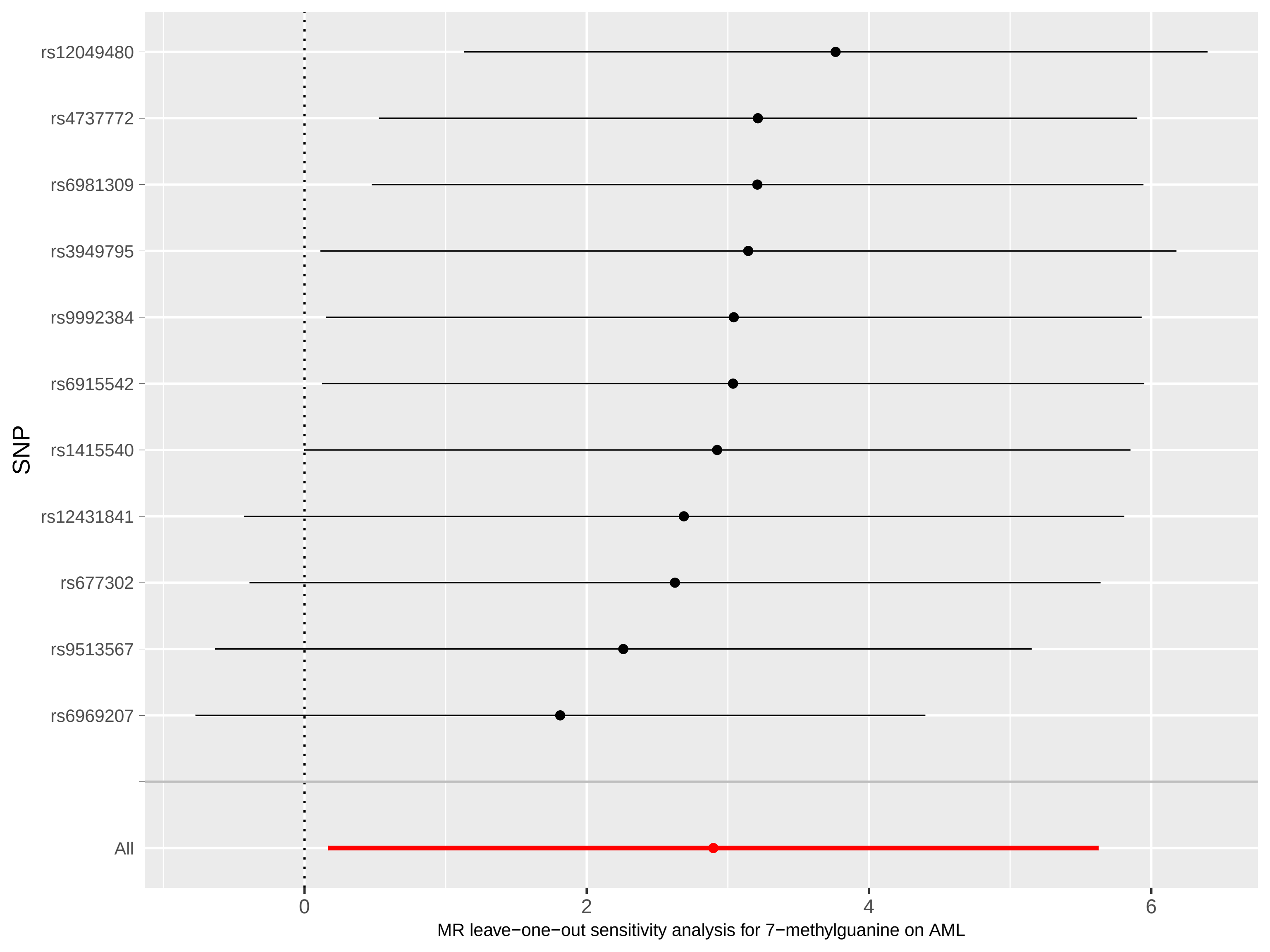

SNP
rs12049480
rs4737772
rs6981309
rs3949795
rs9992384
rs6915542
rs1415540
rs12431841
rs677302
rs9513567
rs6969207
All
0
2
4
6
MR leave−one−out sensitivity analysis for 7−methylguanine on AML

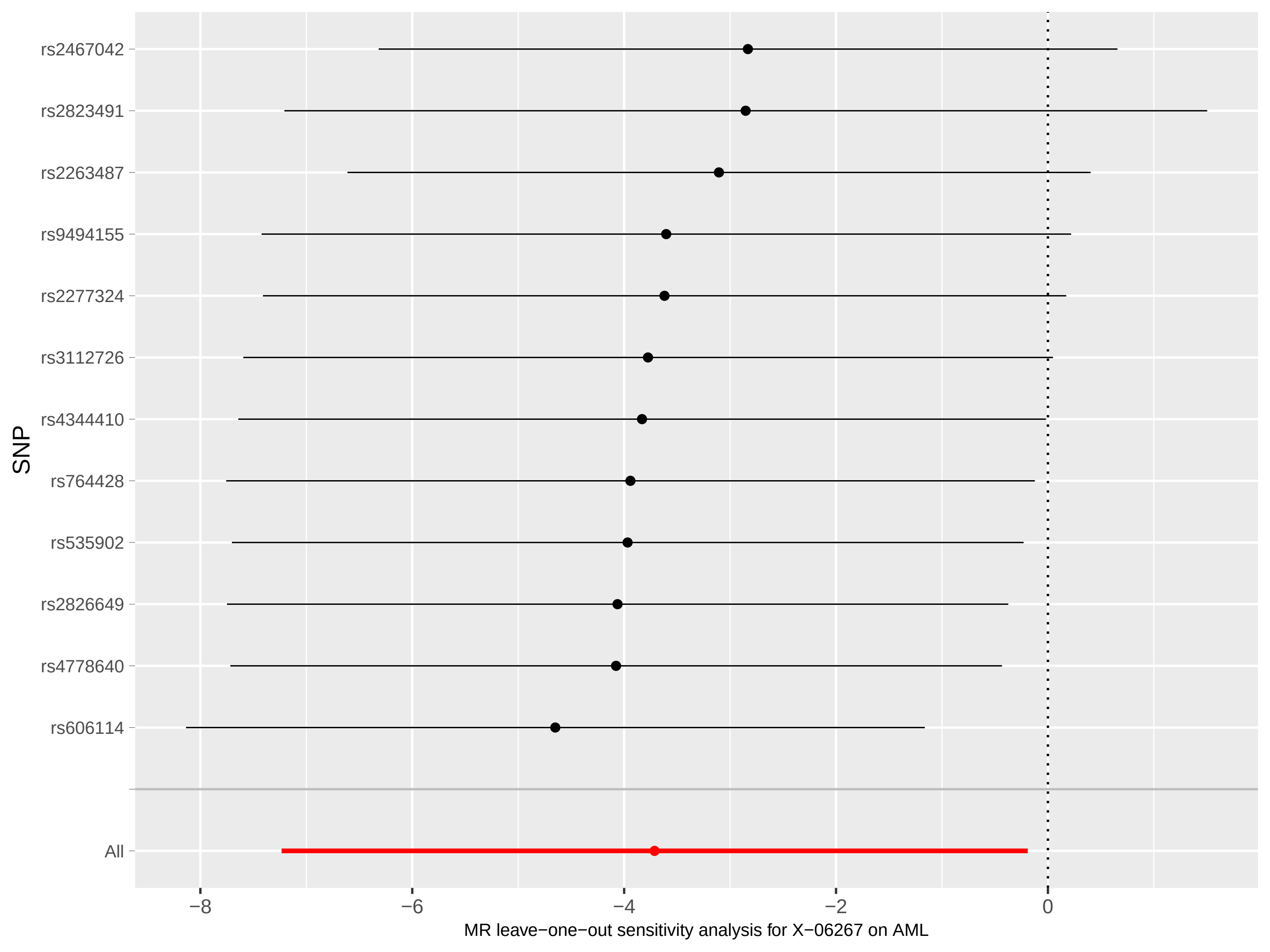

rs2467042
rs2823491
rs2263487
rs9494155
rs2277324
rs3112726
rs4344410
rs764428
rs535902
rs2826649
rs4778640
rs606114
All
SNP
−8
−6
−4
−2
0
MR leave−one−out sensitivity analysis for X−06267 on AML

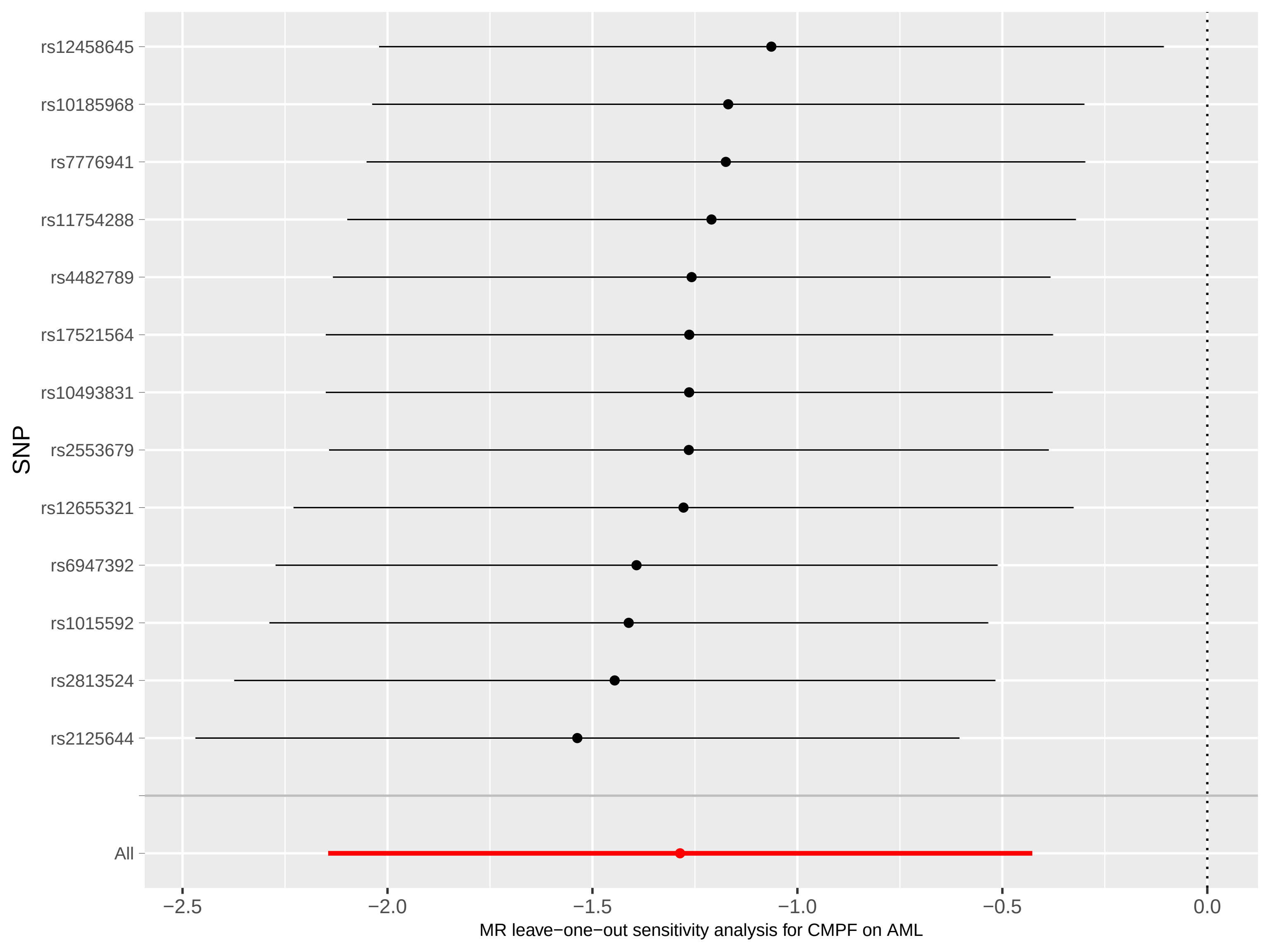
rs12458645
rs10185968
rs7776941
rs11754288
rs4482789
rs17521564
rs10493831
rs2553679
rs12655321
rs6947392
rs1015592
rs2813524
rs2125644
All
SNP
−2.5
−2.0
−1.5
−1.0
−0.5
0.0
MR leave−one−out sensitivity analysis for CMPF on AML

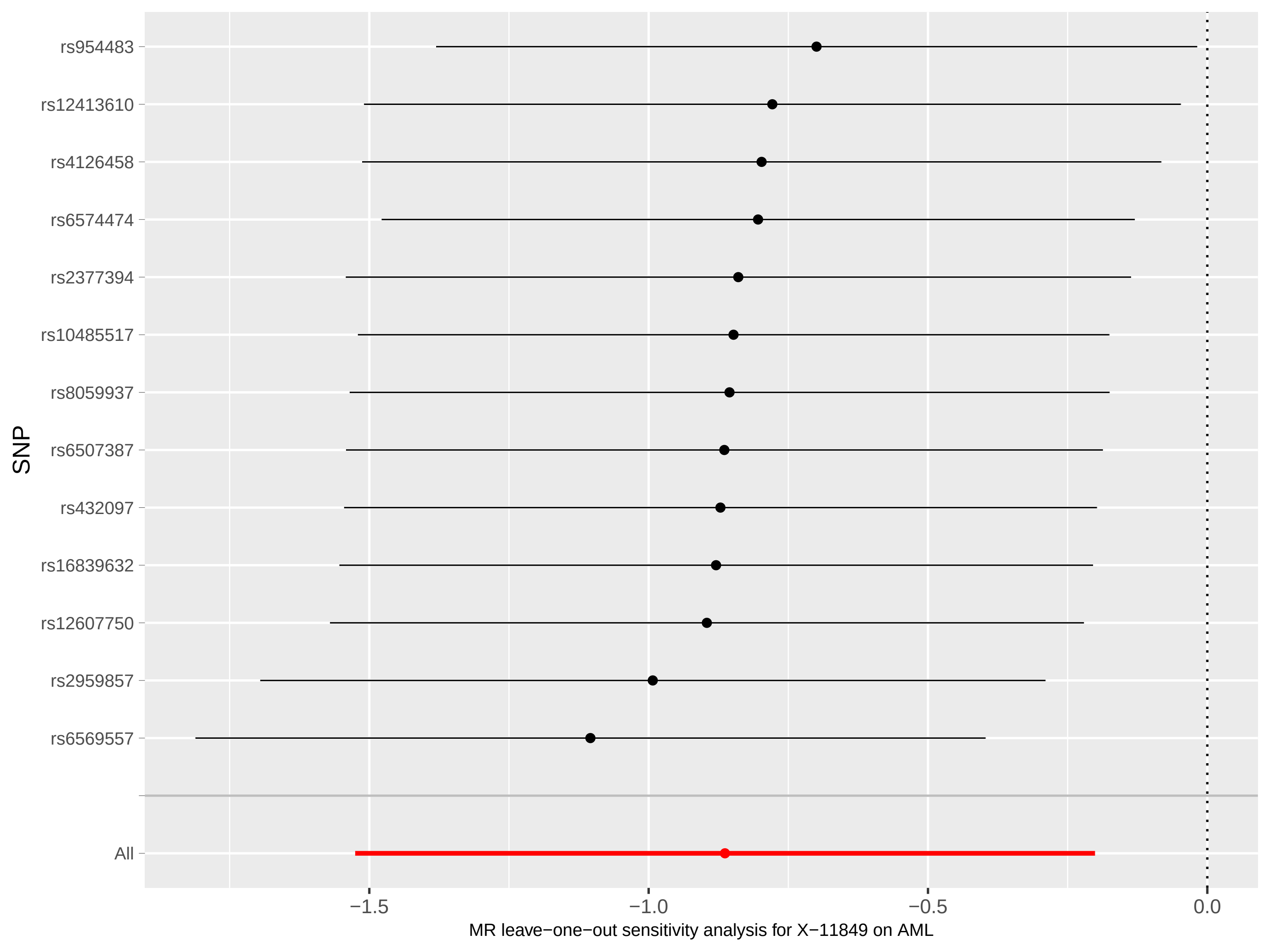

rs954483
rs12413610
rs4126458
rs6574474
rs2377394
rs10485517
rs8059937
rs6507387
rs432097
rs16839632
rs12607750
rs2959857
rs6569557
All
SNP
−1.5
−1.0
−0.5
0.0
MR leave−one−out sensitivity analysis for X−11849 on AML

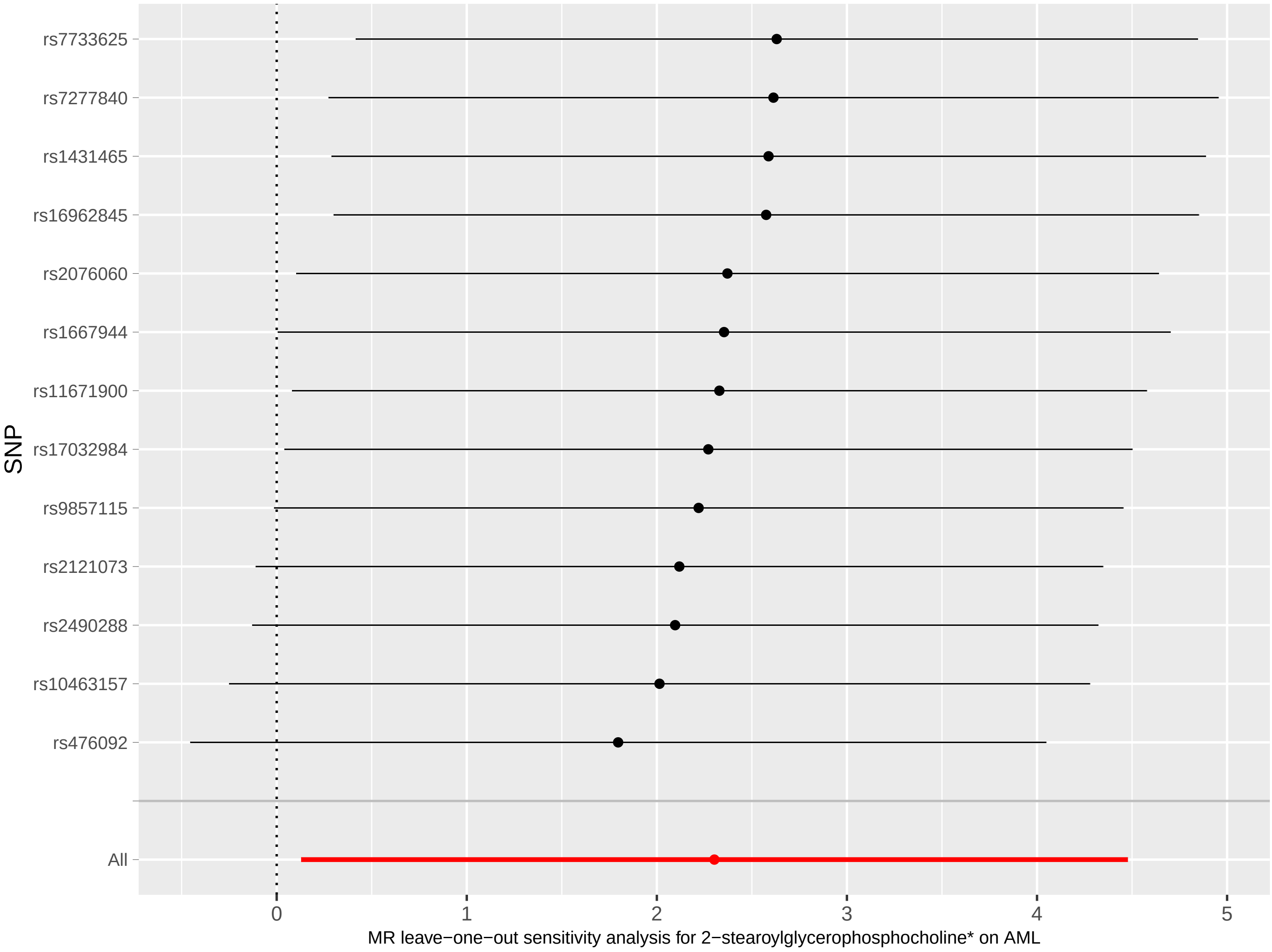

SNP
rs7733625
rs7277840
rs1431465
rs16962845
rs2076060
rs1667944
rs11671900
rs17032984
rs9857115
rs2121073
rs2490288
rs10463157
rs476092
All
0
1
2
3
4
5
MR leave−one−out sensitivity analysis for 2−stearoylglycerophosphocholine* on AML

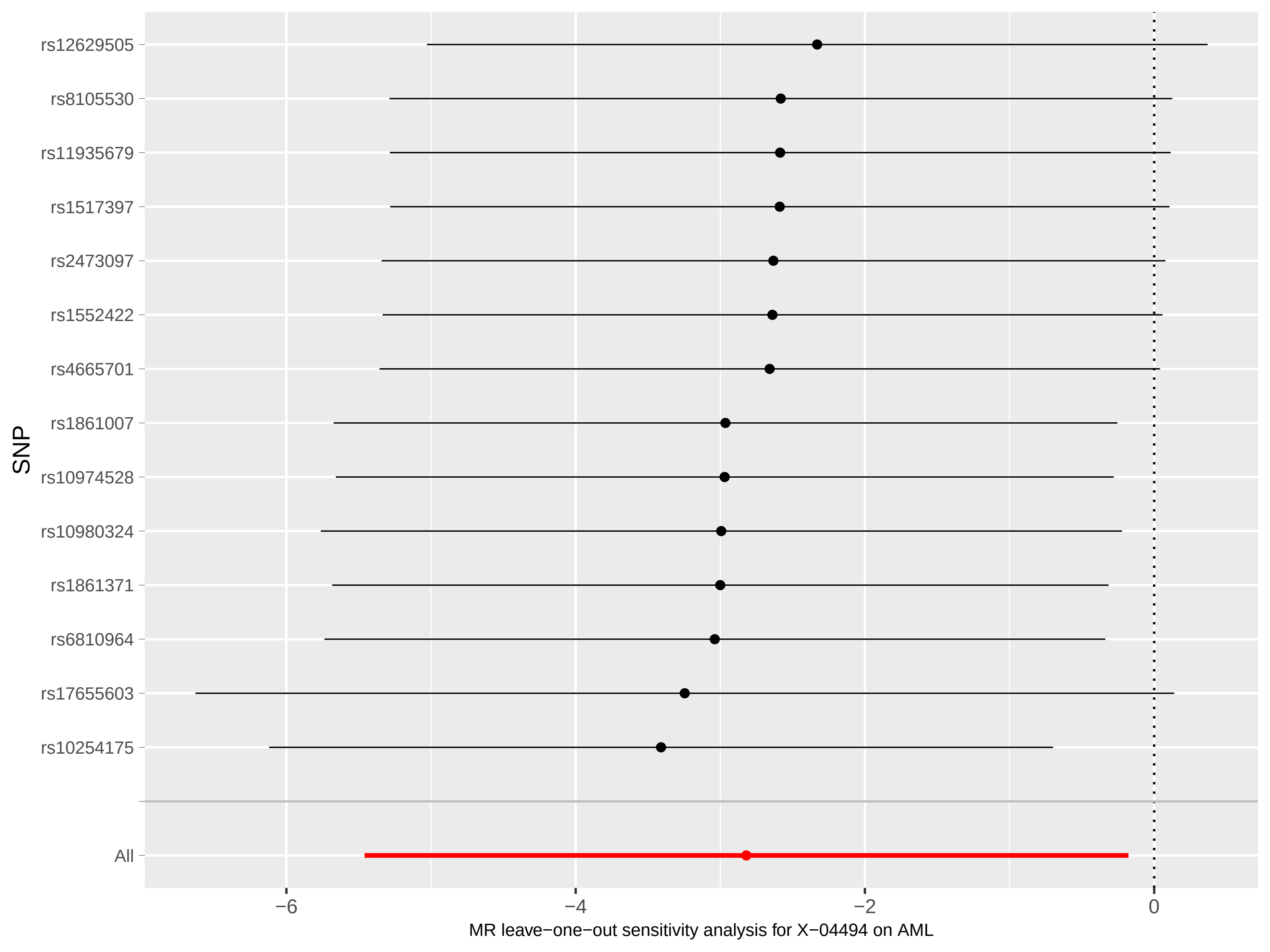

rs12629505
rs8105530
rs11935679
rs1517397
rs2473097
rs1552422
rs4665701
rs1861007
rs10974528
rs10980324
rs1861371
rs6810964
rs17655603
rs10254175
All
SNP
−6
−4
−2
0
MR leave−one−out sensitivity analysis for X−04494 on AML

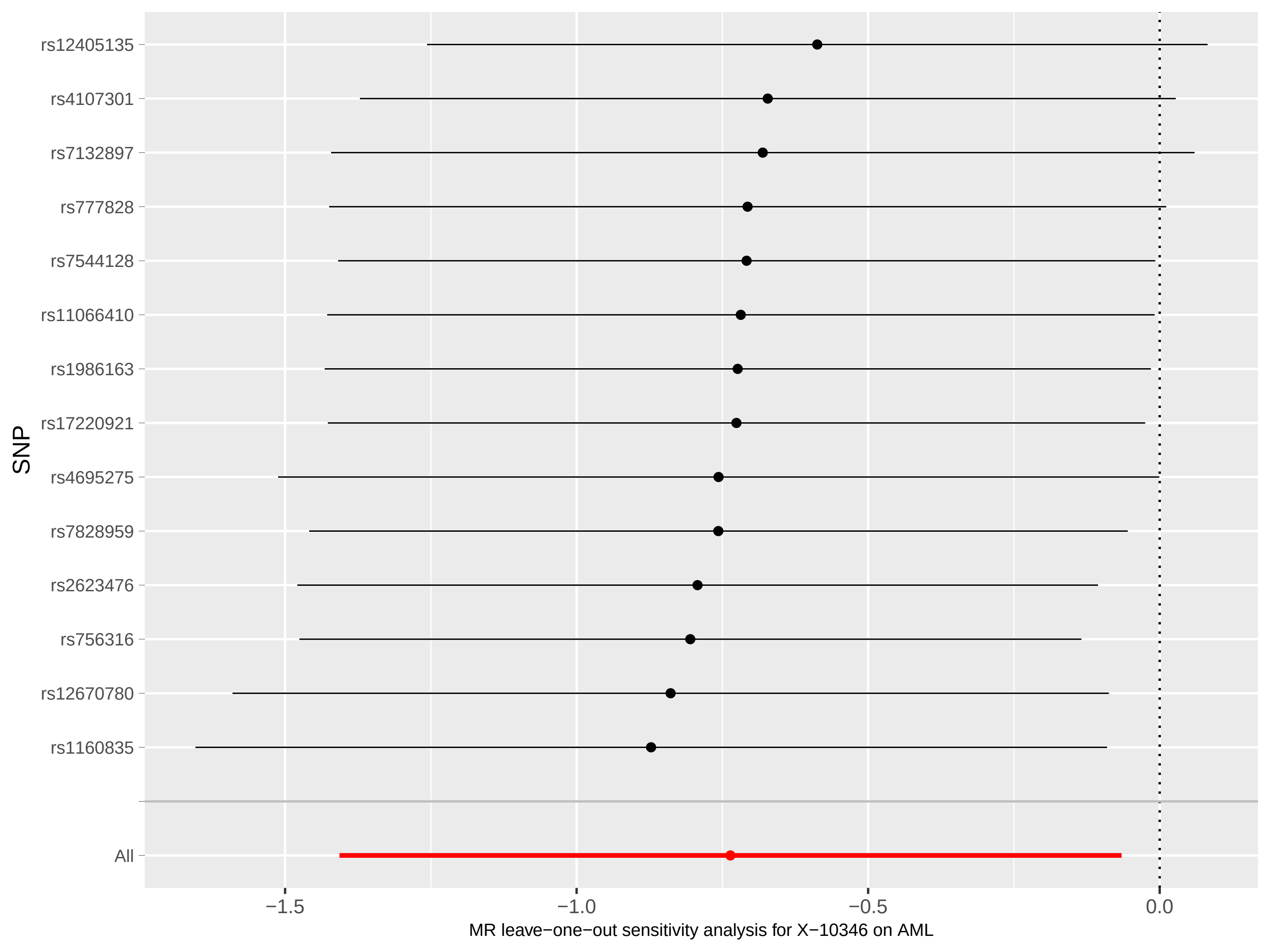

rs12405135
rs4107301
rs7132897
rs777828
rs7544128
rs11066410
rs1986163
rs17220921
rs4695275
rs7828959
rs2623476
rs756316
rs12670780
rs1160835
All
SNP
−1.5
−1.0
−0.5
0.0
MR leave−one−out sensitivity analysis for X−10346 on AML

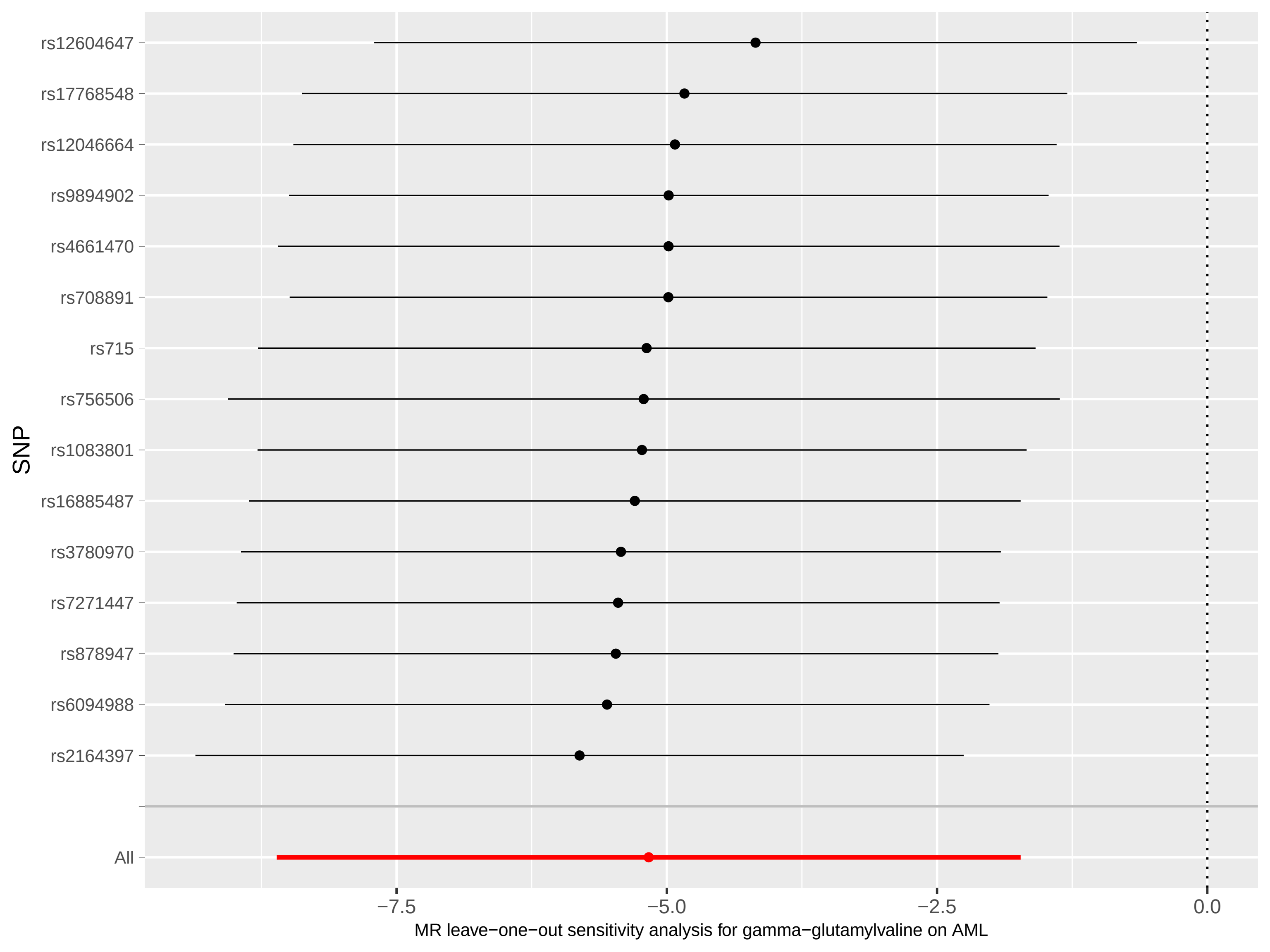

SNP
rs12604647
rs17768548
rs12046664
rs9894902
rs4661470
rs708891
rs715
rs756506
rs1083801
rs16885487
rs3780970
rs7271447
rs878947
rs6094988
rs2164397
All
−7.5
−5.0
−2.5
0.0
MR leave−one−out sensitivity analysis for gamma−glutamylvaline on AML

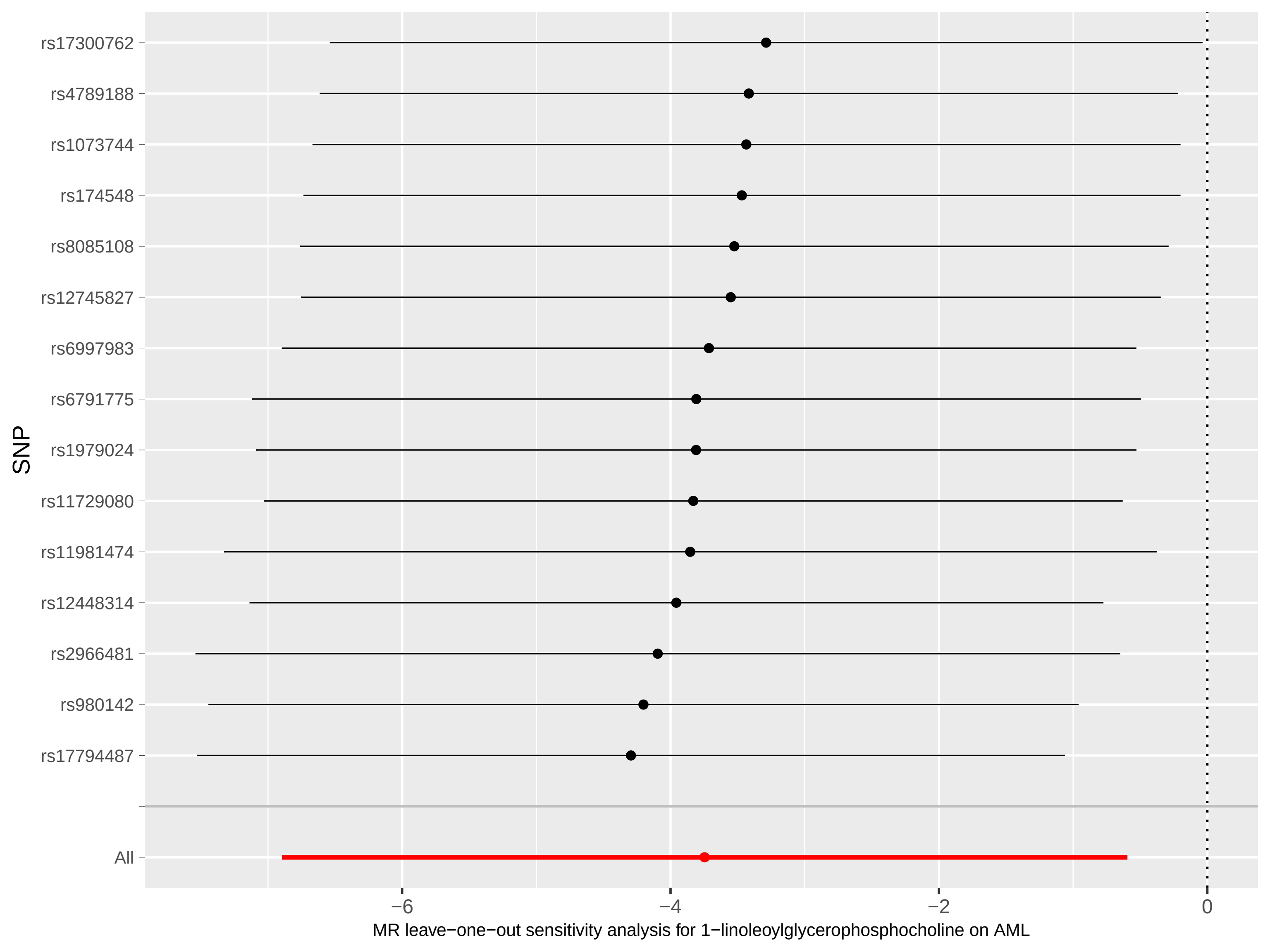

SNP
rs17300762
rs4789188
rs1073744
rs174548
rs8085108
rs12745827
rs6997983
rs6791775
rs1979024
rs11729080
rs11981474
rs12448314
rs2966481
rs980142
rs17794487
All
−6
−4
−2
0
MR leave−one−out sensitivity analysis for 1−linoleoylglycerophosphocholine on AML

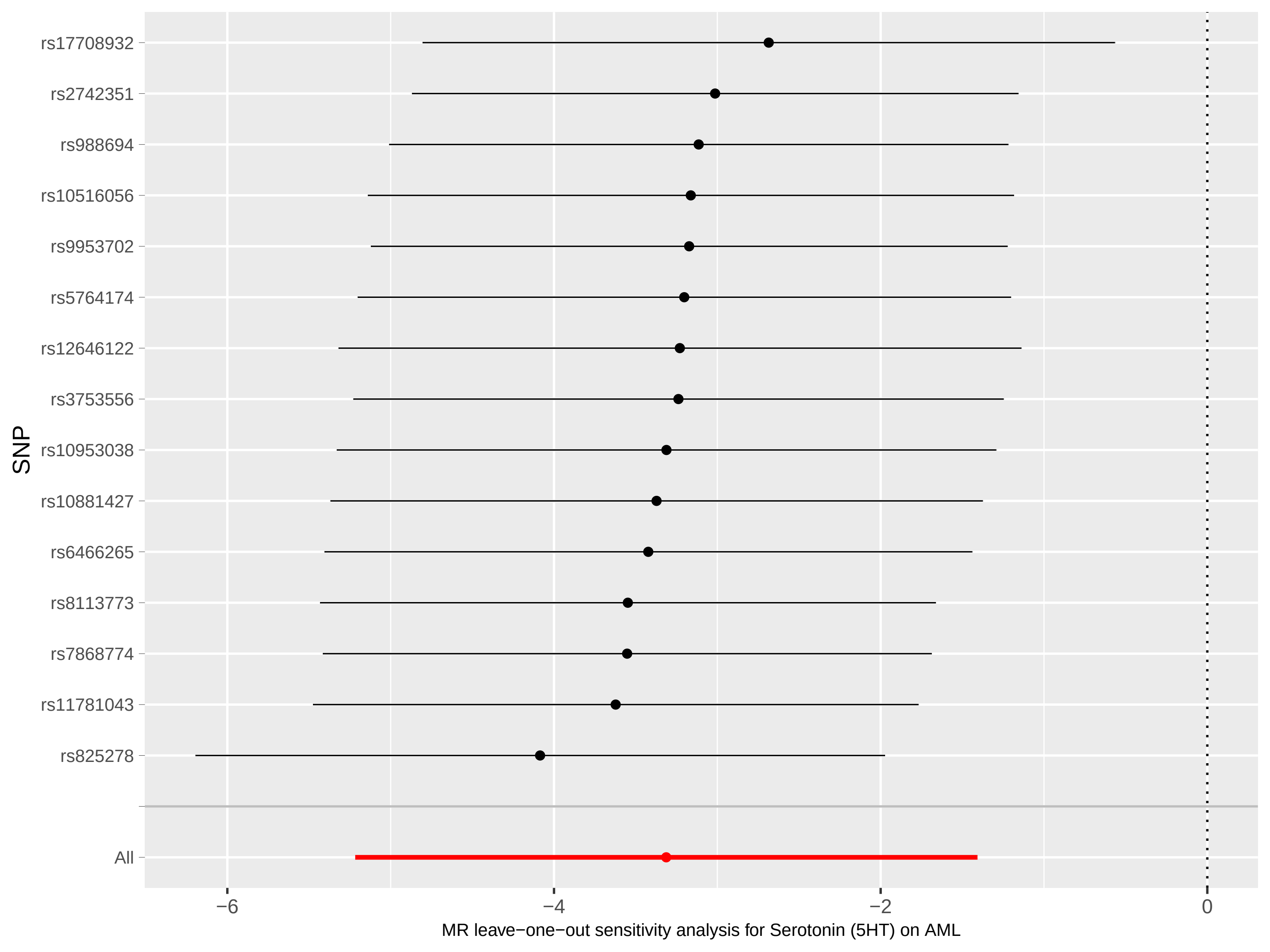

SNP
rs17708932
rs2742351
rs988694
rs10516056
rs9953702
rs5764174
rs12646122
rs3753556
rs10953038
rs10881427
rs6466265
rs8113773
rs7868774
rs11781043
rs825278
All
−6
−4
−2
0
MR leave−one−out sensitivity analysis for Serotonin (5HT) on AML

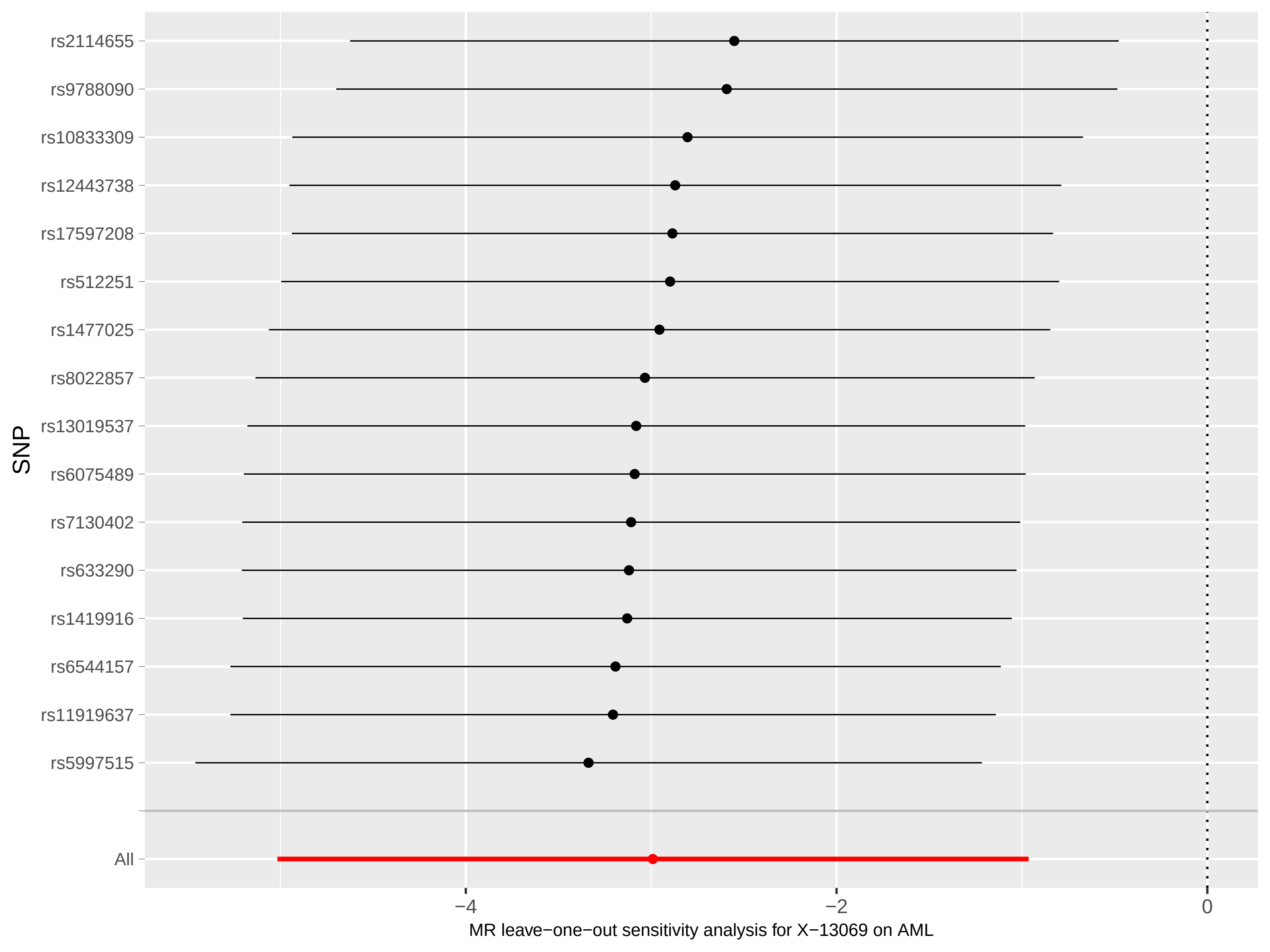

rs2114655
rs9788090
rs10833309
rs12443738
rs17597208
rs512251
rs1477025
rs8022857
rs13019537
rs6075489
rs7130402
rs633290
rs1419916
rs6544157
rs11919637
rs5997515
All
SNP
−4
−2
0
MR leave−one−out sensitivity analysis for X−13069 on AML

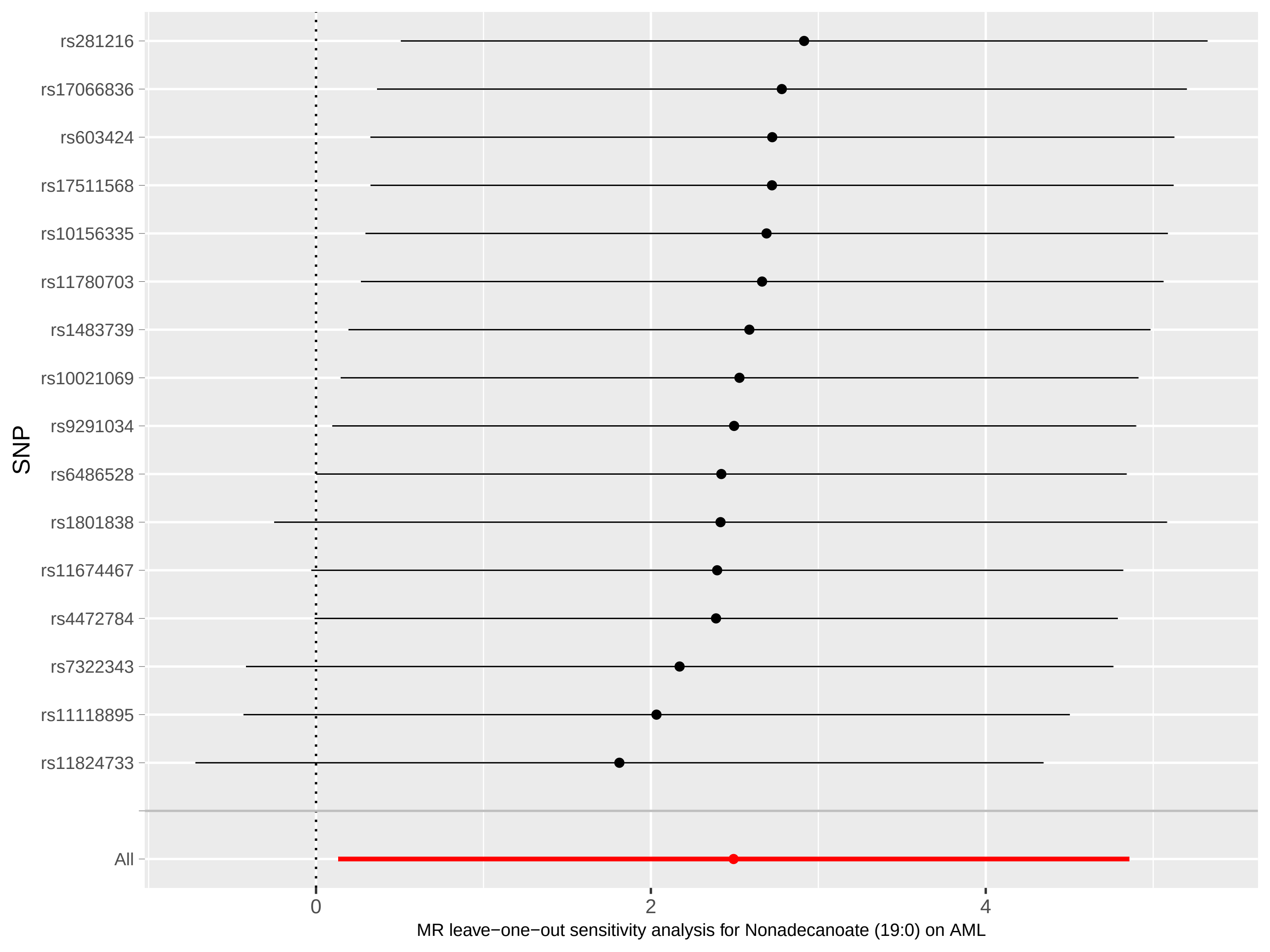
SNP
rs281216
rs17066836
rs603424
rs17511568
rs10156335
rs11780703
rs1483739
rs10021069
rs9291034
rs6486528
rs1801838
rs11674467
rs4472784
rs7322343
rs11118895
rs11824733
All
0
2
4
MR leave−one−out sensitivity analysis for Nonadecanoate (19:0) on AML

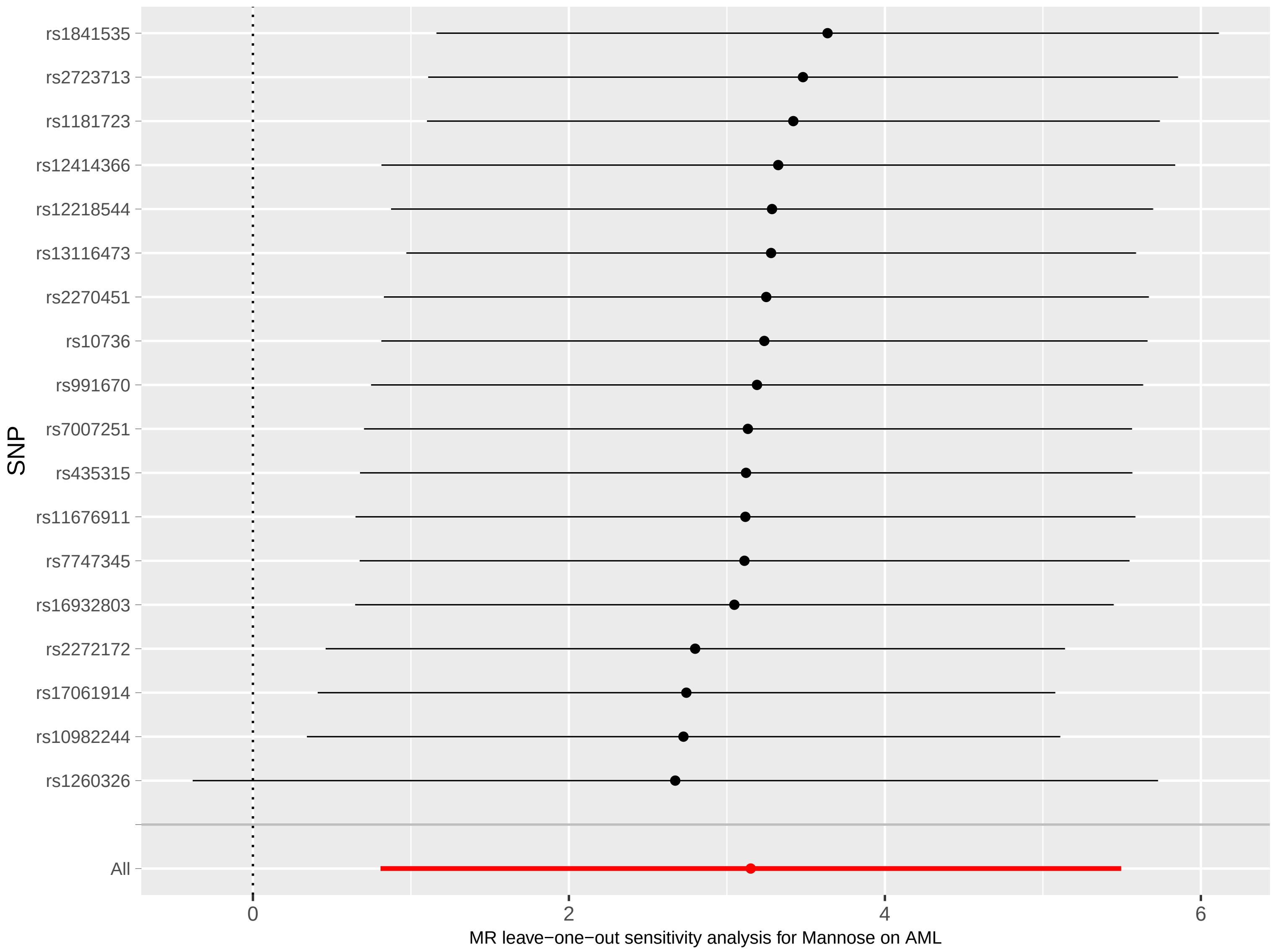

rs1841535
rs2723713
rs1181723
rs12414366
rs12218544
rs13116473
rs2270451
rs10736
rs991670
rs7007251
rs435315
rs11676911
rs7747345
rs16932803
rs2272172
rs17061914
rs10982244
rs1260326
All
SNP
0
2
4
6
MR leave−one−out sensitivity analysis for Mannose on AML

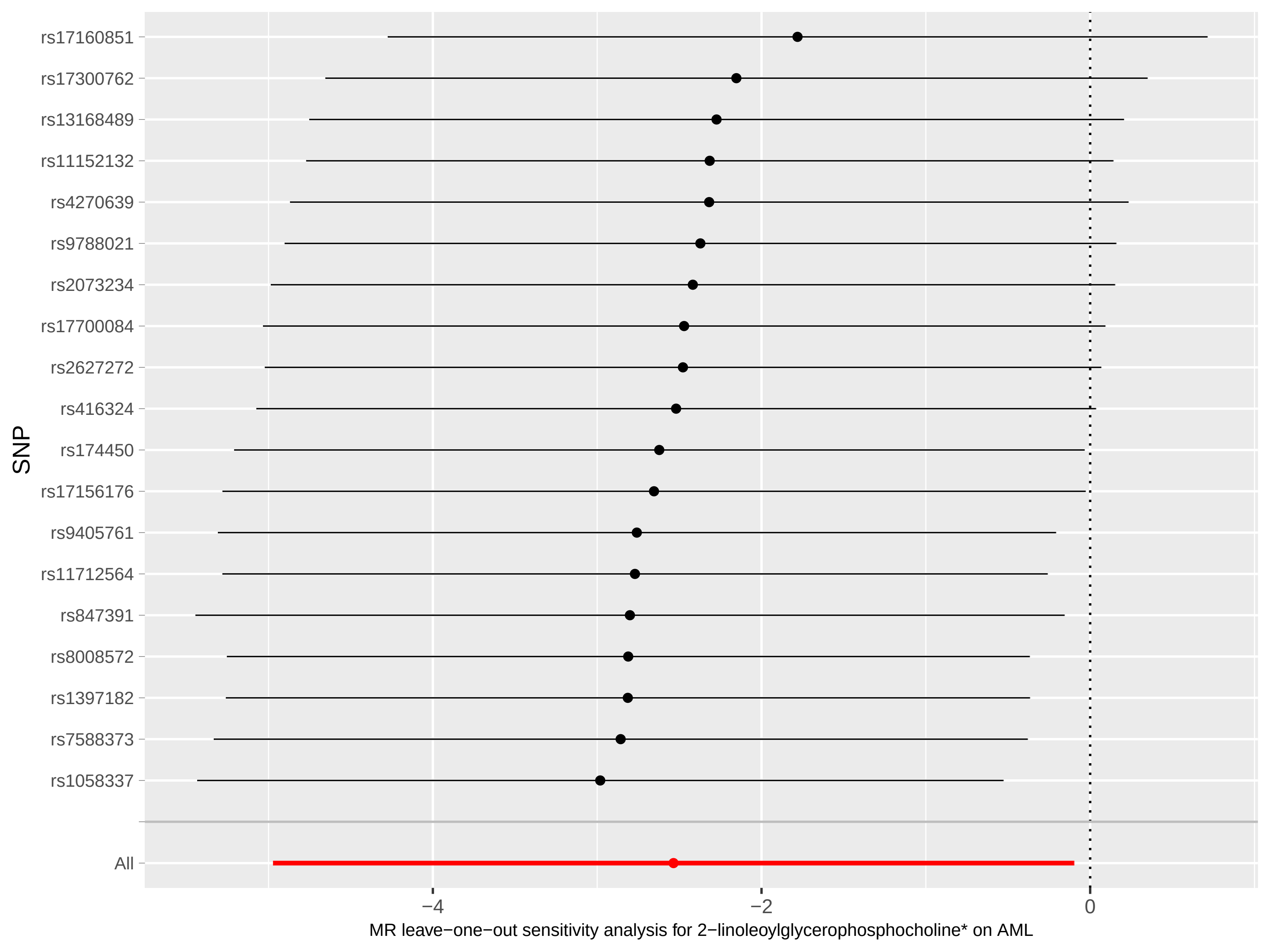
rs17160851
rs17300762
rs13168489
rs11152132
rs4270639
rs9788021
rs2073234
rs17700084
rs2627272
rs416324
rs174450
rs17156176
rs9405761
rs11712564
rs847391
rs8008572
rs1397182
rs7588373
rs1058337
All
SNP
−4
−2
0
MR leave−one−out sensitivity analysis for 2−linoleoylglycerophosphocholine* on AML

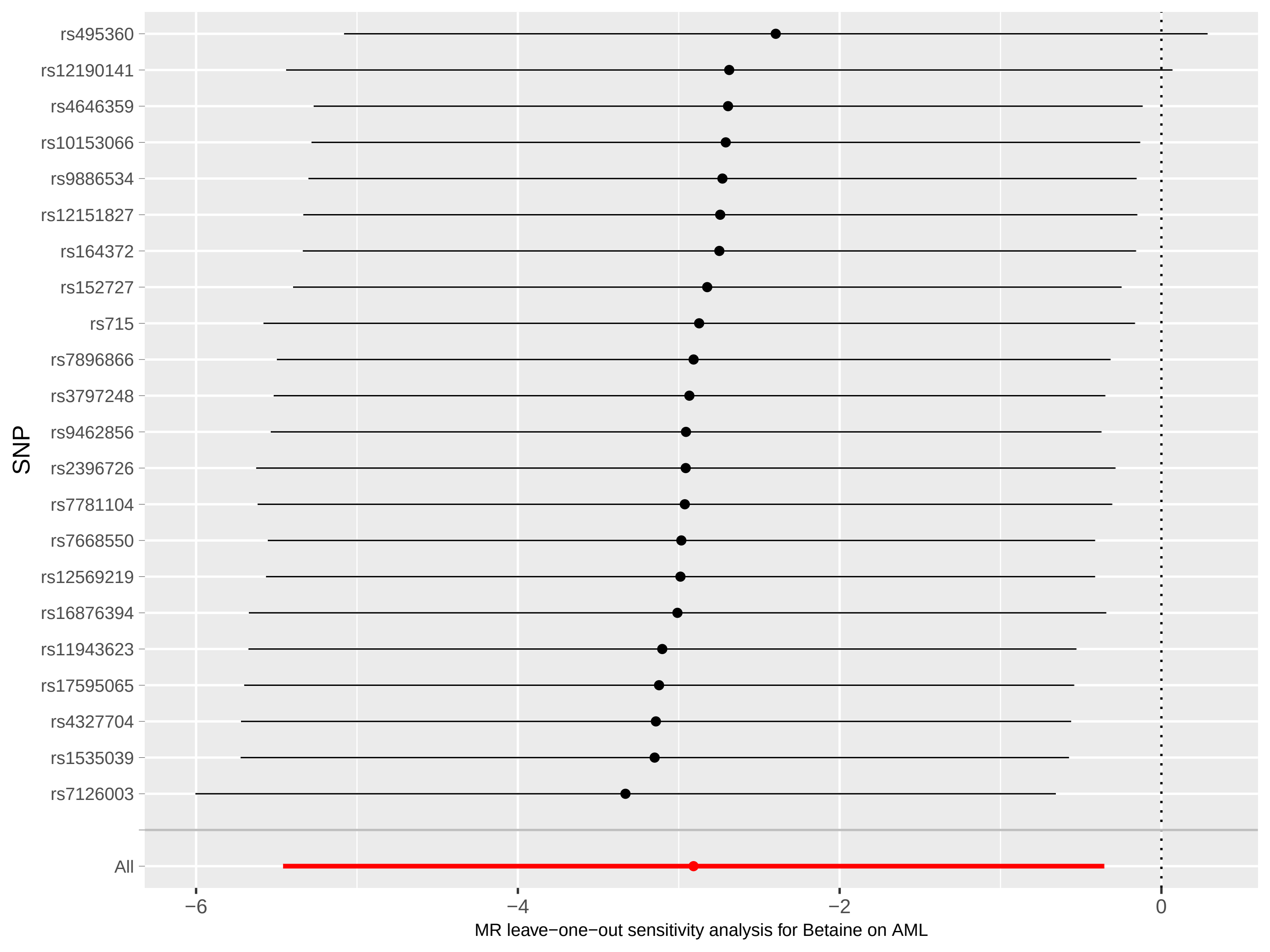

rs495360
rs12190141
rs4646359
rs10153066
rs9886534
rs12151827
rs164372
rs152727
rs715
rs7896866
rs3797248
rs9462856
rs2396726
rs7781104
rs7668550
rs12569219
rs16876394
rs11943623
rs17595065
rs4327704
rs1535039
rs7126003
All
SNP
−6
−4
−2
0
MR leave−one−out sensitivity analysis for Betaine on AML

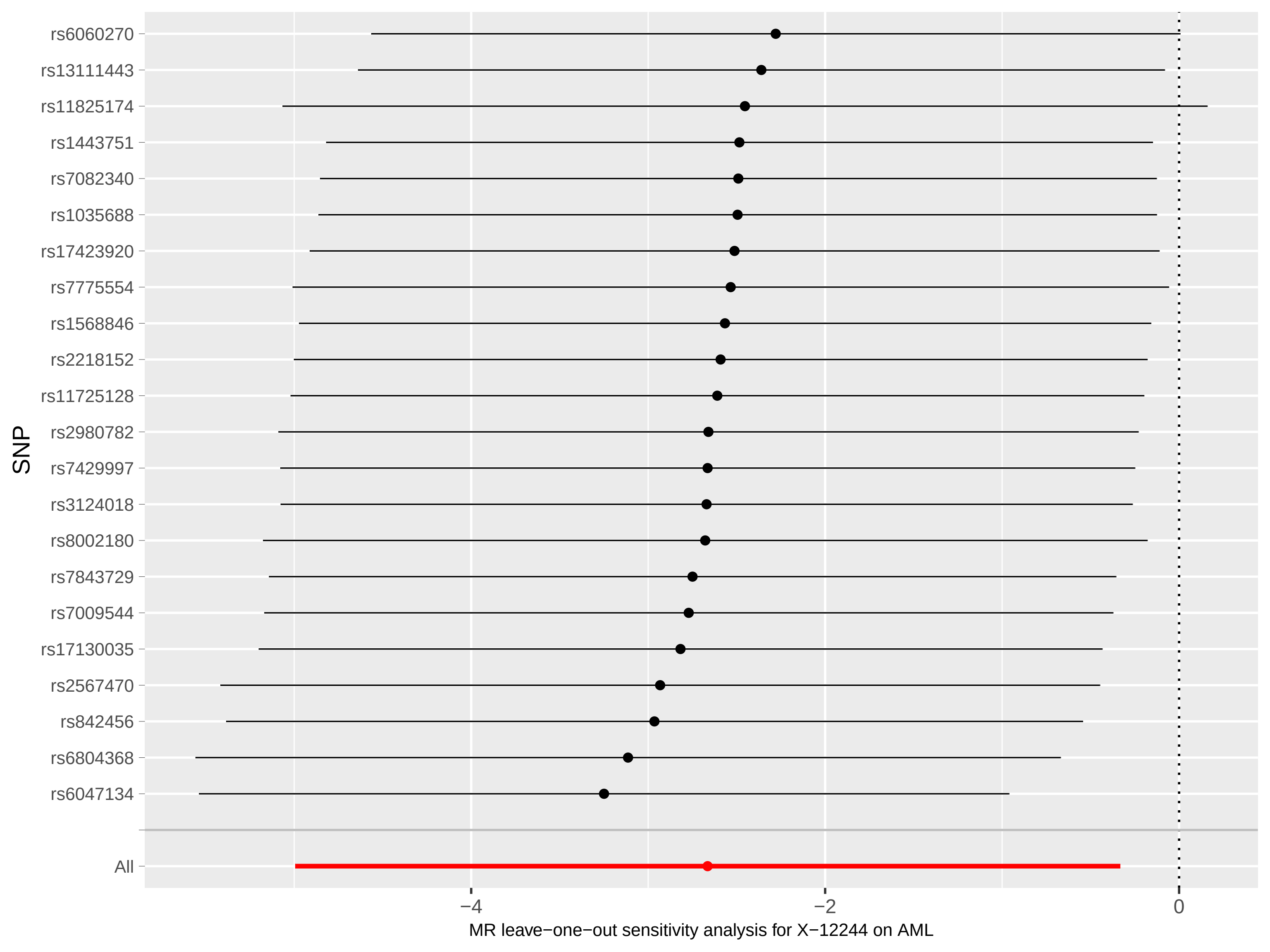

rs6060270
rs13111443
rs11825174
rs1443751
rs7082340
rs1035688
rs17423920
rs7775554
rs1568846
rs2218152
rs11725128
rs2980782
rs7429997
rs3124018
rs8002180
rs7843729
rs7009544
rs17130035
rs2567470
rs842456
rs6804368
rs6047134
All
SNP
−4
−2
0
MR leave−one−out sensitivity analysis for X−12244 on AML

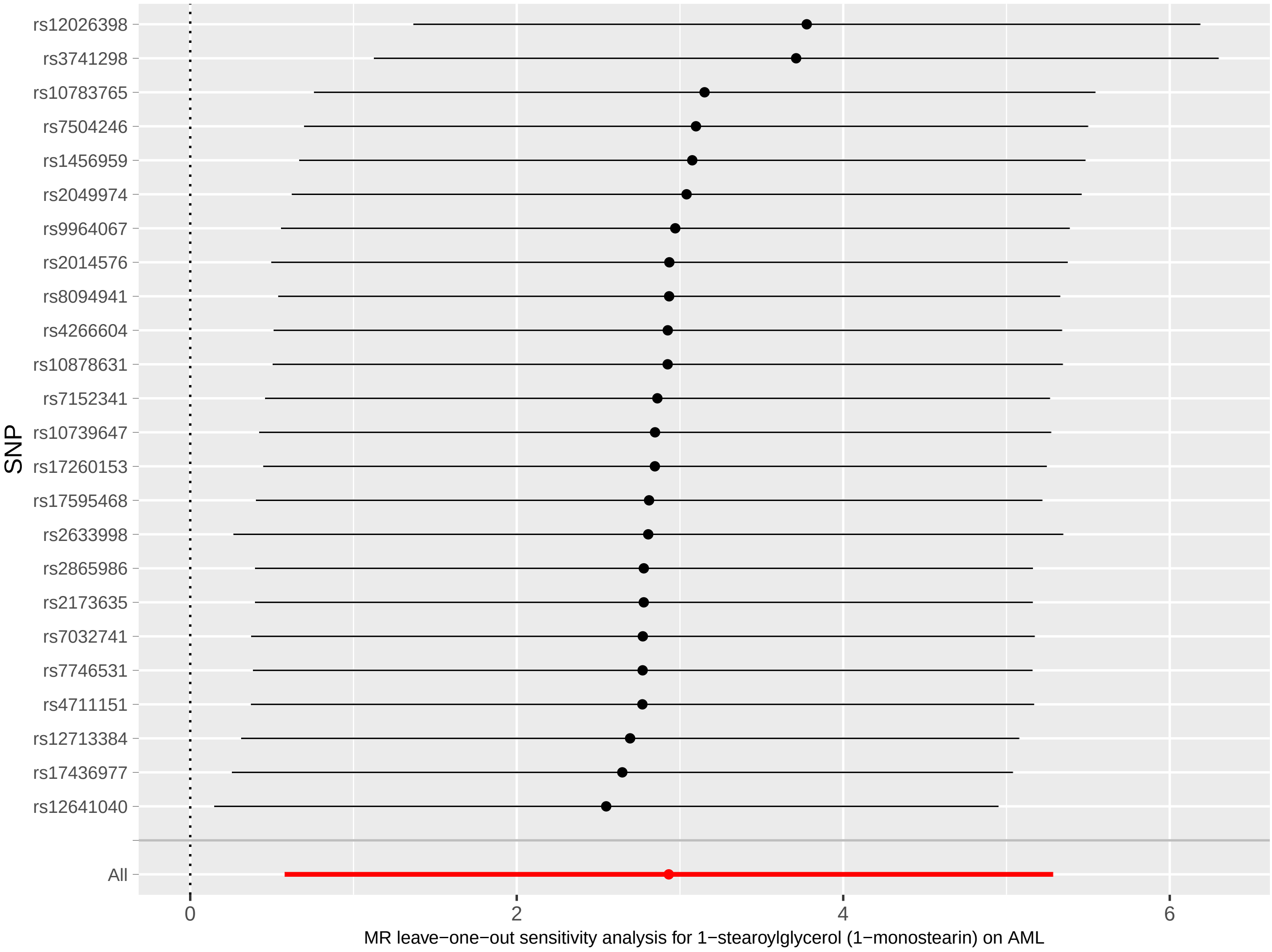

SNP
rs12026398
rs3741298
rs10783765
rs7504246
rs1456959
rs2049974
rs9964067
rs2014576
rs8094941
rs4266604
rs10878631
rs7152341
rs10739647
rs17260153
rs17595468
rs2633998
rs2865986
rs2173635
rs7032741
rs7746531
rs4711151
rs12713384
rs17436977
rs12641040
All
0
2
4
6
MR leave−one−out sensitivity analysis for 1−stearoylglycerol (1−monostearin) on AML

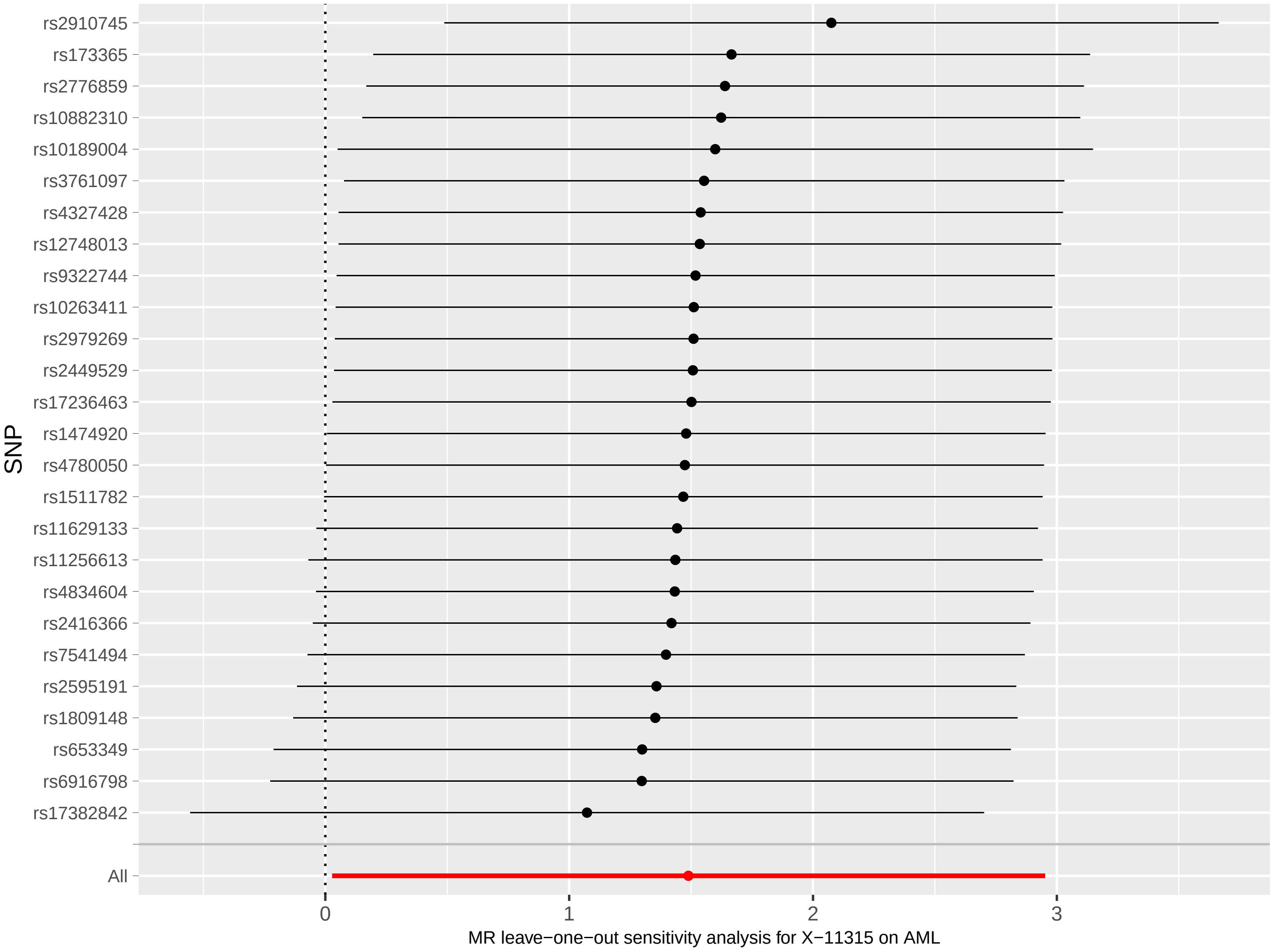

rs2910745
rs173365
rs2776859
rs10882310
rs10189004
rs3761097
rs4327428
rs12748013
rs9322744
rs10263411
rs2979269
rs2449529
rs17236463
rs1474920
rs4780050
rs1511782
rs11629133
rs11256613
rs4834604
rs2416366
rs7541494
rs2595191
rs1809148
rs653349
rs6916798
rs17382842
All
SNP
0
1
2
3
MR leave−one−out sensitivity analysis for X−11315 on AML

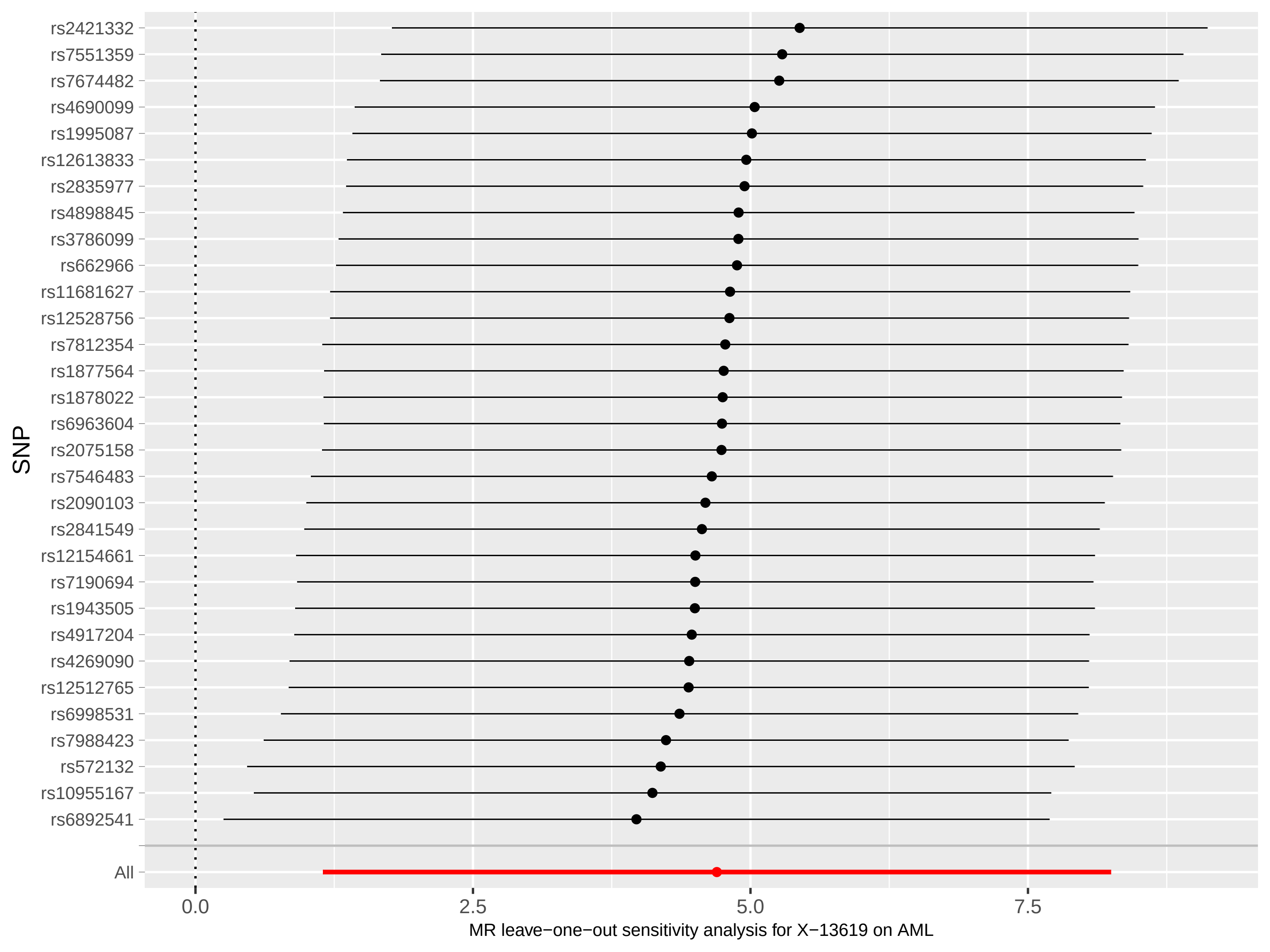

SNP
rs2421332
rs7551359
rs7674482
rs4690099
rs1995087
rs12613833
rs2835977
rs4898845
rs3786099
rs662966
rs11681627
rs12528756
rs7812354
rs1877564
rs1878022
rs6963604
rs2075158
rs7546483
rs2090103
rs2841549
rs12154661
rs7190694
rs1943505
rs4917204
rs4269090
rs12512765
rs6998531
rs7988423
rs572132
rs10955167
rs6892541
All
0.0
2.5
5.0
7.5
MR leave−one−out sensitivity analysis for X−13619 on AML

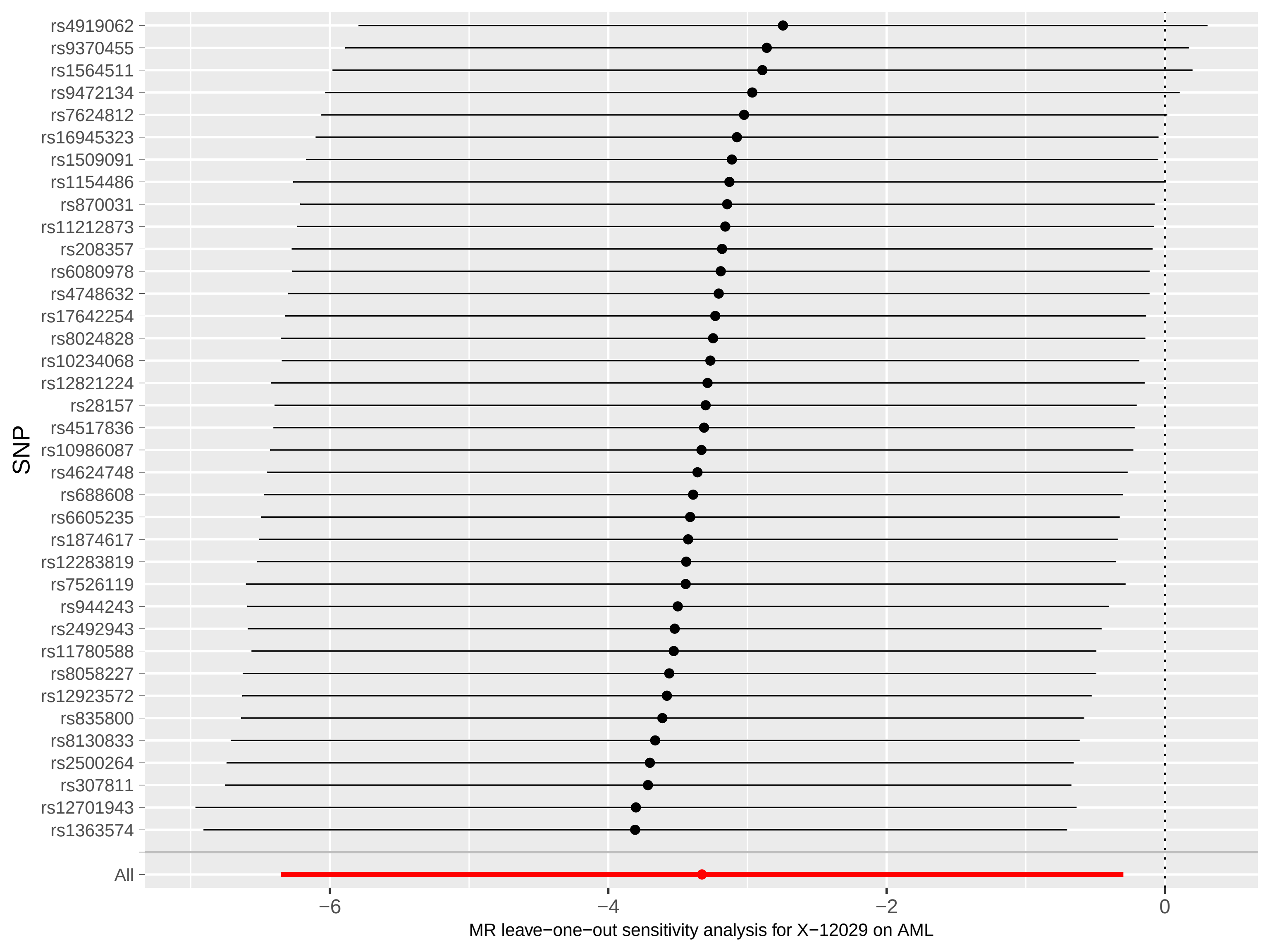
rs4919062
rs9370455
rs1564511
rs9472134
rs7624812
rs16945323
rs1509091
rs1154486
rs870031
rs11212873
rs208357
rs6080978
rs4748632
rs17642254
rs8024828
rs10234068
rs12821224
rs28157
rs4517836
rs10986087
rs4624748
rs688608
rs6605235
rs1874617
rs12283819
rs7526119
rs944243
rs2492943
rs11780588
rs8058227
rs12923572
rs835800
rs8130833
rs2500264
rs307811
rs12701943
rs1363574
All
SNP
−6
−4
−2
0
MR leave−one−out sensitivity analysis for X−12029 on AML

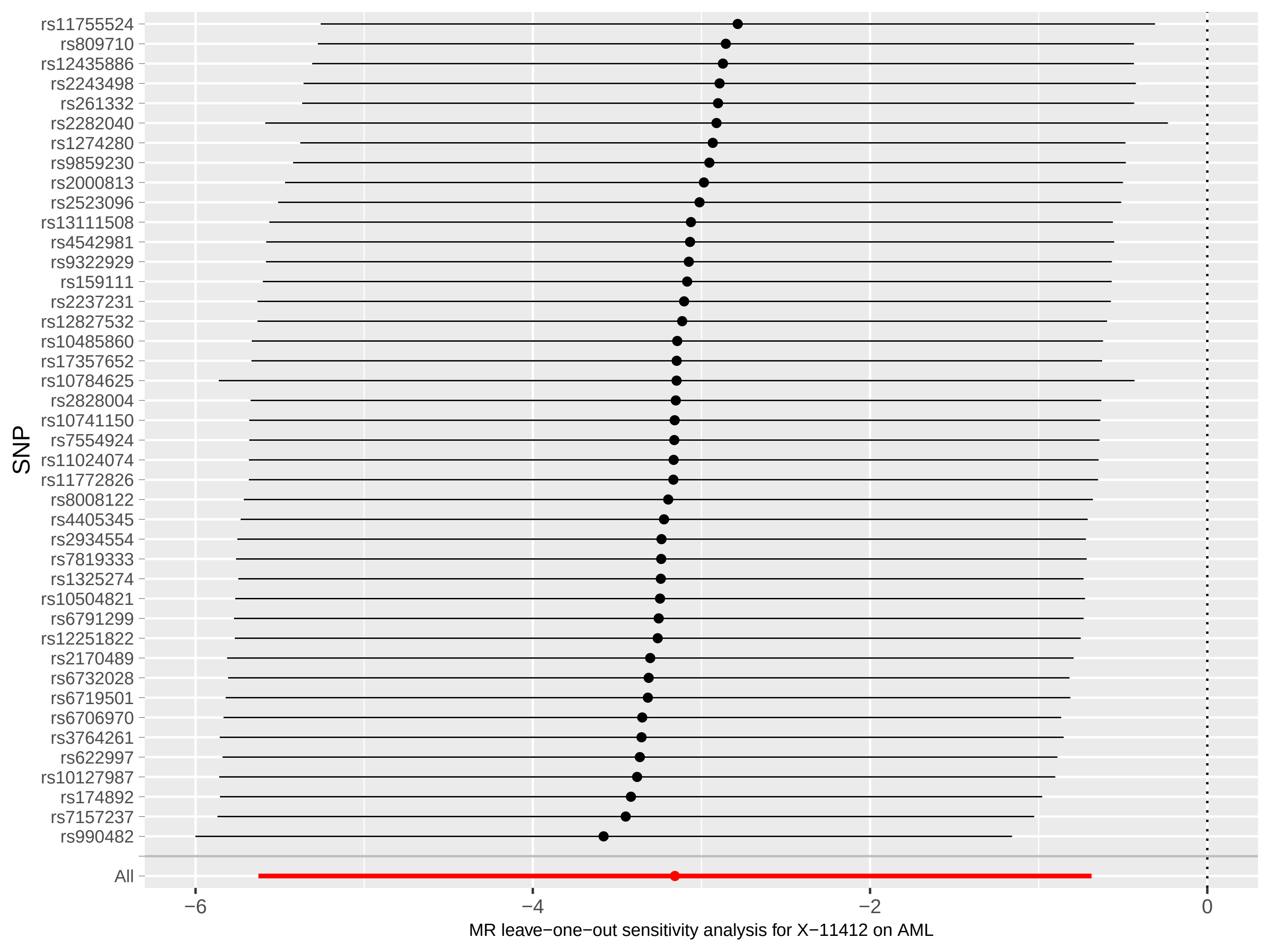

rs11755524
rs809710
rs12435886
rs2243498
rs261332
rs2282040
rs1274280
rs9859230
rs2000813
rs2523096
rs13111508
rs4542981
rs9322929
rs159111
rs2237231
rs12827532
rs10485860
rs17357652
rs10784625
rs2828004
rs10741150
rs7554924
rs11024074
rs11772826
rs8008122
rs4405345
rs2934554
rs7819333
rs1325274
rs10504821
rs6791299
rs12251822
rs2170489
rs6732028
rs6719501
rs6706970
rs3764261
rs622997
rs10127987
rs174892
rs7157237
rs990482
All
SNP
−6
−4
−2
0
MR leave−one−out sensitivity analysis for X−11412 on AML
