# Supplementary material for: Bidirectional Mendelian Randomization and Multi-Omics Uncover Causal Serum Metabolites and Neuro-Related Mechanistic Pathways in Acute Myeloid Leukemia
Source: Int J Mol Sci. 2025 Nov 22;26(23):11307. doi: 10.3390/ijms262311307 (PMC12692008; doi:10.3390/ijms262311307)
Supplement: Supplementary file 1 [file ijms-26-11307-s001.zip › Table S1.pdf]

Table S1. Reverse MR analysis between AML and 23 serum metabolites.

Metabolite	nSNP	IVW		MR Egger		Weighted median		Weighted mode		Simple mode	
		b	pval	b	pval	b	pval	b	pval	b	pval
1-linoleoylglycerophosphocholine	15	-0.0021	0.7605	-0.0562	0.2760	-0.0050	0.3917	-0.0098	0.4155	-0.0099	0.4018
1-stearoylglycerol (1-monostearin)	24	0.0050	0.4931	0.0257	0.7244	-0.0004	0.9504	-0.0014	0.8718	-0.0019	0.8377
2-linoleoylglycerophosphocholine*	19	0.0016	0.8542	-0.0624	0.3165	0.0018	0.8168	0.0032	0.7911	-0.0009	0.9420
2-stearoylglycerophosphocholine*	13	0.0011	0.8663	-0.0046	0.9253	0.0021	0.7933	0.0022	0.8203	0.0022	0.8293
3-carboxy-4-methyl-5-propyl-2-furanpropanoate (CMPF)	13	-0.0063	0.6677	-0.1137	0.4067	-0.0117	0.5266	-0.0236	0.4718	-0.0229	0.4485
7-methylguanine	11	-0.0013	0.8277	0.0146	0.7471	0.0000	0.9992	0.0012	0.8948	0.0011	0.9095
betaine	22	0.0056	0.1910	0.0042	0.8940	0.0058	0.2590	0.0059	0.4056	0.0059	0.4244
gamma-glutamylvaline	15	-0.0080	0.0708	-0.0018	0.9563	-0.0084	0.1203	-0.0090	0.2937	-0.0087	0.3163
histidine	7	-0.0039	0.2442	0.0057	0.8594	-0.0052	0.0954	-0.0063	0.2236	-0.0067	0.2329
mannose	18	-0.0032	0.4598	-0.0263	0.4799	-0.0033	0.5336	-0.0038	0.5989	-0.0051	0.5088
nonadecanoate (19:0)	16	0.0036	0.4349	0.0058	0.8797	0.0029	0.6018	0.0022	0.7788	0.0027	0.7172
serotonin (5HT)	15	0.0010	0.8863	0.0299	0.5813	-0.0011	0.8922	-0.0025	0.8188	-0.0022	0.8374
stachydrine	7	0.0177	0.4519	0.0703	0.6995	0.0111	0.6902	0.0101	0.7749	0.0102	0.7837
X-04494	14	-0.0053	0.3542	-0.0275	0.5597	-0.0068	0.3369	-0.0086	0.3749	-0.0084	0.3737
X-06267	12	0.0032	0.6209	-0.0493	0.3475	-0.0003	0.9689	-0.0055	0.6550	-0.0055	0.6207
X-10346	14	-0.0070	0.6835	0.0797	0.5692	-0.0065	0.7473	-0.0167	0.5974	0.0003	0.9918
X-11315	26	0.0030	0.6124	0.0099	0.8212	0.0017	0.8134	0.0002	0.9829	-0.0000	0.9927
X-11412	42	0.0018	0.6385	0.0046	0.8754	0.0016	0.7286	0.0015	0.7978	0.0016	0.8059
X-11849	13	-0.0279	0.1456	-0.1210	0.4726	-0.0349	0.1415	-0.0423	0.2620	-0.0421	0.2780
X-12029	37	-0.0053	0.1042	-0.0247	0.4128	-0.0047	0.2217	-0.0034	0.5765	-0.0057	0.3851
X-12244	22	-0.0018	0.7973	-0.0397	0.5279	0.0030	0.6153	0.0054	0.5660	0.0047	0.6368
X-13069	16	-0.0076	0.3260	-0.0314	0.6116	-0.0039	0.6654	-0.0038	0.7422	-0.0038	0.7657
X-13619	31	-0.0011	0.7838	-0.0147	0.6996	0.0012	0.7660	0.0019	0.6964	0.0022	0.6699
