# Supplementary material for: Bidirectional Mendelian Randomization and Multi-Omics Uncover Causal Serum Metabolites and Neuro-Related Mechanistic Pathways in Acute Myeloid Leukemia
Source: Int J Mol Sci. 2025 Nov 22;26(23):11307. doi: 10.3390/ijms262311307 (PMC12692008; doi:10.3390/ijms262311307)
Supplement: Supplementary file 1 [file ijms-26-11307-s001.zip › Table S2.pdf]

Table S2. Metabolic pathway associated with AML.

Pathway	Total	Expected	Hits	Raw p	FDR	Impact
**Histidine metabolism**	16	0.0402	1	0.0396	1	0.2213
**Fructose and mannose metabolism**	20	0.0503	1	0.0494	1	0.0000
**beta-Alanine metabolism**	21	0.0528	1	0.0518	1	0.0000
**One carbon pool by folate**	26	0.0653	1	0.0638	1	0.0819
**Galactose metabolism**	27	0.0678	1	0.0662	1	0.0000
**Glycine, serine and threonine metabolism**	33	0.0829	1	0.0805	1	0.0515
**Glycerophospholipid metabolism**	36	0.0905	1	0.0875	1	0.0174
