# Supplementary material for: Bidirectional Mendelian Randomization and Multi-Omics Uncover Causal Serum Metabolites and Neuro-Related Mechanistic Pathways in Acute Myeloid Leukemia
Source: Int J Mol Sci. 2025 Nov 22;26(23):11307. doi: 10.3390/ijms262311307 (PMC12692008; doi:10.3390/ijms262311307)
Supplement: Supplementary file 1 [file ijms-26-11307-s001.zip › Table S3.pdf]

Table S3. Functional roles of key genes associated with metabolites.

Metabolite	Associated Gene	gene role in AML
1-linoleoylglycerophosphocholine	ST8SIA5, POU6F2, PPARGC1A, CLSTN2, RP11-322E11.5	positive regulation of synapse assembly, positive regulation of cell junction assembly, regulation of synapse assembly, AMPK signaling pathway
1-stearoylglycerol (1-monostearin)	FABP2, ATP2B2, EVC2, IL1RAP, Y_RNA, LINC01122, AC074212.5	positive regulation of synapse assembly, regulation of postsynaptic density assembly, regulation of postsynaptic specialization assembly, regulation of postsynaptic density organization, regulation of excitatory synapse assembly, postsynaptic density assembly, positive regulation of cell junction assembly, regulation of synapse assembly, IgSF CAM signaling, Th17 cell differentiation, Adrenergic signaling in cardiomyocytes
2-linoleoylglycerophosphocholine	AHRR, PPP2R2C, ST8SIA5, RIPK2, SPATA17, snoU13, GS1-57L11.1	AMPK signaling pathway, Glycosphingolipid biosynthesis - ganglio series, Adrenergic signaling in cardiomyocytes
2-stearoylglycerophosphocholine	EBI3, RASGRF2, AF064858.8	Th17 cell differentiation
3-carboxy-4-methyl-5-propyl-2-furanpropanoate (CMPF)	CPA6, LRRC8C, RP11-302M6.4, CYP2C58P, RP11-400G3.3, SLC38A1, RP11-527H14.4, RP11-527H14.2, RP11-444A22.1, AC007131.2	amino acid transmembrane transport, amino acid transport, carboxylic acid transmembrane transport, organic acid transmembrane transportglycine transport, GABAergic synapse, Glutamatergic synapse
7-methylguanine	PTPRD	positive regulation of synapse assembly, regulation of postsynaptic density assembly, regulation of postsynaptic specialization assembly, regulation of postsynaptic density organization, regulation of excitatory synapse assembly, postsynaptic density assembly, positive regulation of cell junction assembly, regulation of synapse assembly, IgSF CAM signaling
betaine	SLC6A13, SLC6A12, NKX2-3, FOXP2, ZMAT4, snoU13, RP11-109J4.1	amino acid neurotransmitter reuptake, gamma-aminobutyric acid transport, Neuroactive ligand signaling, Synaptic vesicle cycle, GABAergic synapse
gamma-glutamylvaline	GRM7, LINC00494	Neuroactive ligand signaling
histidine	FPGT-TNNI3K, TNNI3K, RP11-439H8.4, RP11-446N19.1, RP11-66E20.2, LINC00670, CPS1, SLC2A9, RP11-38H17.1, RP11-91P17.1, ADCY8	cellular response to glucagon stimulus, response to glucagon, response to ketone, cellular response to peptide hormone stimulus, response to metal ion, cAMP biosynthetic process, carbohydrate mediated signaling, cellular response to peptide, urea cycle, midgut development, Long-term potentiation, Biosynthesis of amino acids, Nitrogen metabolism, Ovarian steroidogenesis, Regulation of lipolysis in adipocytes, Cortisol synthesis and secretion, Thyroid hormone synthesis, Arginine biosynthesis, Alanine, aspartate and glutamate metabolism, Longevity regulating pathway - multiple species
mannose	GCKR, CPNE4, Y_RNA, LINC00703, RP11-162A23.5,	negative regulation of kinase activity, response to hexose, cellular response to salt, cellular response to metal ion, regulation of epithelial cell proliferation involved in prostate gland development, negative regulation of phosphorylation

nonadecanoate (19:0)	AC012391.1, GPR19, CDKN1B, LINC00593, TLE3 UNC5C	IgSF CAM signaling
serotonin (5HT)	NFIB, TTN, AC097467.2, RP11-478L17.1, RP11-110F24.1, KIAA0040, RP5-1114G22.2, RBBP8, CCNE1, CTD-2057D4.2	DNA replication, nuclear division, mitotic cell cycle phase transition, organelle fission, regulation of stem cell proliferation, stem cell proliferation, DNA-templated DNA replication, G1/S transition of mitotic cell cycle, mitotic nuclear division, cell cycle G1/S phase transition,
stachydrine	BCL2L14, SH3BGRL2, GRIN2B, RP11-72J9.1, LINC01079, RNY1P1, RP11-636O21.1, NRXN1, RP1-159G19.1, RP1-125N5.2, RP3-495K2.3	positive regulation of excitatory postsynaptic potential, positive regulation of synaptic transmission, glutamatergic, modulation of excitatory postsynaptic potential, regulation of synaptic transmission, glutamatergic, excitatory postsynaptic potential, synaptic transmission, glutamatergic, chemical synaptic transmission, postsynaptic
